# Predictive modelling of the effectiveness of vaccines against COVID-19 in Bogotá: Methodological innovation involving different variants and computational optimisation efficiency

**DOI:** 10.1016/j.heliyon.2024.e39725

**Published:** 2024-10-23

**Authors:** Oscar Espinosa, Lisa White, Valeria Bejarano, Ricardo Aguas, Duván Rincón, Laura Mora, Antonio Ramos, Cristian Sanabria, Jhonathan Rodríguez, Nicolás Barrera, Carlos Álvarez-Moreno, Jorge Cortés, Carlos Saavedra, Adriana Robayo, Bo Gao, Oscar Franco

**Affiliations:** a Economic Models and Quantitative Methods Research Group, Centro de Investigaciones para el Desarrollo, Universidad Nacional de Colombia, Bogotá, D.C., Colombia; b Directorate of Analytical, Economic and Actuarial Studies in Health, Instituto de Evaluación Tecnológica en Salud (IETS), Bogotá, D.C., Colombia; c Department of Biology, University of Oxford, Oxford, United Kingdom; d Nuffield Department of Medicine, University of Oxford, Oxford, United Kingdom; e Directorate of Synthesis and Technology Management, Instituto de Evaluación Tecnológica en Salud (IETS), Bogotá, D.C., Colombia; f Faculty of Medicine, Universidad Nacional de Colombia, Bogotá, D.C., Colombia; g Executive Directorate, Instituto de Evaluación Tecnológica en Salud (IETS), Bogotá, D.C., Colombia; h University Medical Center Utrecht, Utrecht University, Utrecht, Netherlands; i Harvard T.H. Chan School of Public Health, Harvard University, Cambridge, USA

**Keywords:** Real-world evidence, Effectiveness, Vaccines, COVID-19, Public health, Predictive modelling, Mathematical epidemiology

## Abstract

The uncertainty associated with the future of viruses such as SARS-CoV-2 poses a challenge to public health officials because of its implications for welfare, economics and population health. In this document, we develop an age-stratified epidemiological-mathematical model to predict various health outcomes, considering the effectiveness of COVID-19 vaccines. The analytical model proposed and developed for this research is based on the approach constructed by the COVID-19 International Modelling Consortium. Following this approach, this paper innovates at the frontier of knowledge by including the various variants of SARS-CoV-2 in the Consortium model. Furthermore, for the first time in international literature, a complete compilation of the formal mathematical development of this entire quantitative model is presented. Our model accurately fits the observed historical data of new infections, cumulative mortality, symptomatic infections, hospitalisations, and Intensive Care Units admissions, capturing the waves of contagion that have occurred in Bogotá, Colombia. In turn, the prognosis obtained indicates a considerable decrease in the incidence and lethality caused by SARS-CoV-2 under current conditions, thus evidencing the effectiveness of vaccines against infection, hospitalisation, and death. This model enables the evaluation of different scenarios in response to changes in the dynamics of this infectious disease, providing information to policymakers for real-world evidence-based decision-making.

## Highlights

• Epidemiological-mathematical model considering the effectiveness of vaccines.
• Innovation by including various variants of SARS-CoV-2 in the CoMo Consortium model.
• Predictive analytics incorporating pharmaceutical and non-pharmaceutical measures.
• Complete compilation of the mathematical development of the CoMo is presented.

## 1 Introduction

Preventing the spread, containment, and mitigation of the SARS-CoV-2 virus poses a significant public health challenge due to its devastating impact on economic and social determinants. The speed of transmission is a key characteristic of the infection, with over 595 million people infected and 6.4 million associated deaths recorded worldwide as of August 2022 [1].

The virus was first documented at the end of December 2019 when the Wuhan City Health Commission (China) reported cases of unexplained pneumonia in local hospitals [2]. Two months later, the epidemic had spread globally, with confirmed cases in 46 countries [3]. Consequently, on 11 March 2020, the World Health Organisation declared a pandemic [4].

In Colombia, the first reported case occurred in early March 2020 in the city of Bogotá. The virus rapidly spread throughout the country, leading the national government to declare a health emergency on 12 March of that year [5]. Bogotá, along with other major cities, declared a yellow alert for the spread of SARS-CoV-2 shortly before the presidential decree [6].

On 20 March 2020, a mock quarantine was implemented in Bogotá, marking the beginning of a series of non-pharmacological interventions at both national and district levels. These measures included quarantine, isolation of the elderly, sanitation measures, personal hygiene, and physical distancing, among others [7]. The aim was to control the spread of the virus and maintain the operational capacity of the healthcare system while awaiting effective treatment or vaccines.

After two and a half years of the pandemic, by August 2022, Bogotá had accumulated 1,841,472 confirmed cases and 29,653 deaths according to the Instituto Nacional de Salud (INS) [8]. The city experienced five waves of contagion, with the third wave, reported between March and June 2021 [7], being the most severe in terms of incidence and lethality. This wave resulted in approximately 648,711 confirmed cases and 10,104 deaths, representing 35.2 % of the total incidence and 34.1 % of deaths. Hospital care during this period showed an average occupancy rate of 75.16 % in general hospital beds and 81.13 % in Intensive Care Units (ICUs), higher than the average observed from the beginning of the pandemic to August 2022 (65.4 % in general hospitalisation and 61.2 % in ICU), despite the increase in the number of available beds in the city [9,10].

As the pandemic progressed, the scientific community worked on developing effective vaccines and treatments to mitigate the virus. By the end of 2020, around 50 companies were conducting various studies [11], and the first effective vaccines had been developed. At that time, Bogotá had a seroprevalence of 30 % and 512,032 confirmed cases [8,12].

In 2021, the Instituto Nacional de Vigilancia de Medicamentos y Alimentos (INVIMA) granted emergency use authorisation to vaccines from Pfizer-BioNTech [13], AstraZeneca [14], Janssen [15], Sinovac [16], and Moderna [17]. These vaccines showed varying efficacy results in clinical trials, with Pfizer-BioNTech and Moderna demonstrating the highest performance after the second dose, achieving efficacy rates against symptomatic disease exceeding 90 % [18]. In February 2021, the country's Ministry of Health and Social Protection reported the acquisition of approximately 61.5 million doses from the portfolio of these five vaccines authorised by INVIMA, through direct agreements with producers and the COVAX mechanism [19].

In Bogotá, the vaccination campaign began on 18 February 2021, following the prioritisation criteria established by the national government in the National Vaccination Plan. The initial batch of vaccines allocated to the capital comprised 12,582 doses from Pfizer-BioNTech [20], administered to frontline health workers [21]. Subsequently, after more than a year and a half of immunisations, by 2 August 2022, 16,047,191 doses had been administered in the city, including complete, partial, and booster doses. This vaccination effort has led to a decrease in the mortality rate from the virus -after the peak reached in mid-2021- as well as a reduction in the case fatality rate, which averaged 0.6 % in August 2022 compared to 1.9 % in 2020 [22]. The burden on hospitals also decreased due to a lower incidence of severe disease.

Despite advances in mitigating health outcomes related to infection, several questions remain about the future of the virus and its societal impact. Changes in SARS-CoV-2 transmission due to new variants, vaccine acceptance, variations in vaccine effectiveness, the implementation or removal of non-pharmacological measures, and shifts in individual behaviours necessitate the development of models to estimate various scenarios and provide evidence for health decision-making.

This research presents an age-structured compartmental epidemiological-mathematical model to forecast the number of deaths and hospitalisations in Bogotá due to SARS-CoV-2. The model evaluates virus dynamics in the context of vaccine introduction, variant emergence, and adjustments to non-pharmacological measures, among other factors. The goal is to provide evidence-based information for policymakers.

The analytical model proposed and developed in this research is based on the approach established by the COVID-19 International Modelling Consortium (CoMo Consortium). In alignment with this approach, this article contributes to the frontier of knowledge by incorporating various SARS-CoV-2 variants into the CoMo Consortium model. Additionally, for the first time in the international literature, a complete compilation of the formal mathematical development of the entire CoMo Consortium model is presented (see full details of the system of equations in Supplement 1).

The paper consists of four sections, in addition to the introduction: ii) literature review; iii) description of the analytical model and its calibration; iv) results of the mathematical epidemiological approach; and v) discussion.

## 2 Literature review

In recent years, numerous studies have assessed the effectiveness of COVID-19 vaccines by predicting health outcomes, with the most common metrics being the number of infections, hospitalisations, and deaths. To this end, compartmental models have been predominantly employed. These models facilitate the modelling of infectious diseases by segmenting the population of interest into different compartments, operating under the assumption that individuals within the same compartment share similar characteristics.

The compartmental models utilised include Susceptible-Infected-Recovered [23], Susceptible-Exposed-Infected-Recovered [11], Susceptible-Exposed-Infected-Recovered-Dead-Vaccinated [24], and Susceptible-Infected-Quarantine-Hospitalised-Recovered-Extinct [25], among others. In addition, agent-based models [26], Bayesian networks [[27], [28], [29]], disturbance analysis [30], neural networks [31], Markov models [32], and fractal models [33] have also been extensively used to simulate vaccine effects.

The most recurrent parameters in various analytical models include recovery rates, transmission rates, mortality rates, hospitalisation rates, lethality, reinfection rates, vaccination coverage, the percentage of the population in each model compartment, the contact matrix, infection period, and the effectiveness of the vaccine(s).

Given the variations in socio-demographic profiles and public health interventions adopted by governments, different values for these parameters are observed. In some cases, these values are derived from real-world data,^1^ while in others, they are assumed. At the geographical level, models have been implemented for countries with diverse characteristics, including England [34,35], United States [36], Germany [37], China [38,39], Australia [40], and India [41]. For the Latin America region, studies have been conducted on Argentina [42], Brazil [43], México [44,45], and Colombia [46,47].

Several studies include non-pharmacological interventions that were implemented before and during the vaccination process. Among the interventions considered by the authors are the use of surgical masks, isolation, quarantines, mobility restrictions, teleworking, contact tracing, school closures, hand hygiene, and others. [48]. Furthermore, considering the differential risk based on certain characteristics, some authors differentiate by sex, age group, and migrant population.

Regarding the virus, some models include a single variant [49] or multiple variants [50]. Similarly, various studies account for either one dose [51] or multiple doses [49] of the vaccine. Vaccine effectiveness is often considered a relevant variable in the model, which may be general (i.e. not disaggregated by commercial brand [25]) or disaggregated by brand [52]. Additionally, in some cases, effectiveness is further differentiated based on its objective: susceptibility, infection, and mortality.

## 3 Materials and methods

### 3.1 Model structure

The forecasting of SARS-CoV-2 infections, hospitalisations, and fatalities in the city of Bogotá is conducted using an extended dynamic Susceptible-Exposed-Infected-Recovered-Susceptible model, based on the model developed by the CoMo Consortium. This analytical model has served as the foundation for studies conducted by Diarra et al. [53], Aguas et al. [54], Borges et al. [55], and others.

**Fig. 1:**
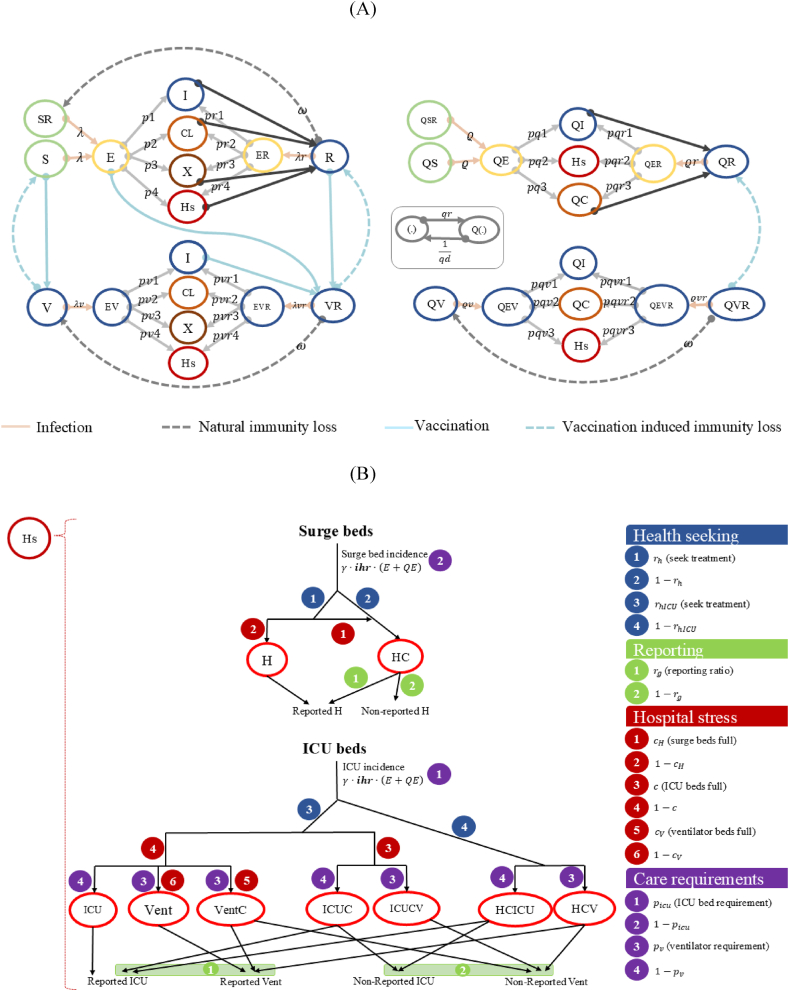
Graph of the mathematical epidemiological model of SARS-CoV-2 transmission in Bogotá: (A) general model, (B) hospital sub-model. *Note.* Both diagrams illustrate the structure of the model for the transmission of SARS-CoV-2, including scenarios with vaccination and non-pharmacological interventions. The variables in the compartments are listed in Supplement 1 (subsection A). For simplicity, “Hs” represents all individuals requiring hospital treatment after infection (individuals who have or do not have access to the health system, depending on availability). The dynamics of “Hs” are explained by the diagram (Fig. 1B), which details surge beds and ICU beds, the parameters involved, and their reportability by the model. The correspondent abbreviations are: S: susceptible, SR: susceptible with prior exposure, E: infected and incubating, I: infectious and asymptomatic, CL: infectious and with mild symptoms, X: mildly symptomatic infected in self-isolation, ER: exposed recovered, R: recovered, V: vaccinated, EV: exposed vaccinated, EVR: recovered vaccinated exposed, VR: vaccinated recovered, when a Q is added at the beginning it means that it is in quarantine (QC: mildly symptomatic population in quarantine), H: hospitalised, HC: not hospitalised due to lack of capacity, ICU: hospitalised in intensive care unit, Vent: hospitalised in the ICU and on a ventilator, VentC: hospitalised in the ICU that requires a ventilator but are not assigned one, ICUC: hospitalised population requiring ICU but only receiving a surge bed, ICUCV: hospitalised population requiring ICU and ventilator but only receiving a surge bed, HCICU: requiring ICU but not hospitalised due to lack of capacity and HCV: requiring a ventilator but not hospitalised due to lack of capacity.

In this approach, the population is stratified into five-year age groups, and the infected compartment considers various characteristics such as the severity of symptoms, the stage of infection, vaccination or isolation status, the need for medical care, and the capacity of the healthcare system to meet demands for hospitalisation services, ICU beds, and mechanical ventilation. The progression of individuals through the natural history of infection is presented in Fig. 1(A and B), and the system of differential equations for the model is provided in Supplement 1. One of the innovations in our quantitative approach is the modelling of different variants, which is incorporated through the numerical functions $\sigma_{Rmod}\left(t\right)$ for breakthrough infection probability, dm(t) for lethality, and pm(t) for transmissibility within the system of ordinary differential equations (see subsection C of Supplement 1). The health outcomes predicted by the model include the number of new infections, symptomatic infections, hospitalisations, ICU admissions, and fatalities.

The transmission of the virus is modelled through contacts between individuals in four different settings: i) work; ii) home; iii) school; and iv) other places (including public transportation, meetings, or other activities that involve social interaction). In this regard, the contact rate between individuals of different age groups is based on the contact matrices developed by Prem et al. [56], using data from the urban areas in Colombia as a proxy for Bogotá.

Both pharmacological and non-pharmacological interventions are incorporated into the model. On one hand, susceptible individuals can be vaccinated, acquiring a degree of immunity against the virus for a specified duration at established effectiveness levels. On the other hand, the model considers scenarios involving non-pharmacological interventions within the city, such as physical distancing, closure of educational institutions, quarantines, travel bans, and protection of the elderly, among others. Each of these interventions is detailed in Supplement 1.

### 3.2 Parameterisation and adjustment

The calibration and adjustment of the model are based on data from the first reported case of COVID-19 in Bogotá up until 4 August 2022. The parameters used for this adjustment are sourced from the INS, Bogotá Open Data, SaluData, the National Administrative Department of Statistics, and specialised literature on SARS-CoV-2. The initial values of the parameters are presented in Table A of Supplement 1, while the parameter space and their final values are detailed in Supplement 2.

The optimisation process minimises the Mean Absolute Error (MAE) function for forecasts of new cases, cumulative deaths, hospitalisations, ICU admissions, and symptomatic cases compared to the observed data. This optimisation employs the Gosolnp method, which performs the minimisation from Solnp, an algorithm based on the augmented Lagrange method. A more detailed description of the calibration method and computational optimisation can be found in subsection G of Supplement 1.

The parameters are grouped into six categories (see Supplement 1). First, parameters related to the epidemiology and severity of the virus in the city, including incidence, mortality, and hospitalisation rates. Second, values related to the study population, such as the number of births. Third, parameters covering the time horizon of the model and the percentage of symptomatic and asymptomatic patients reported. Fourth, a category that includes parameters related to the behaviour of the infectious disease in individuals, such as the incubation period and the probability of developing clinical symptoms. Fifth, parameters concerning the hospitalisation of patients who develop COVID-19. Finally, parameters related to pharmacological and non-pharmacological interventions in the city, including adherence to interventions and vaccine effectiveness.

## 4 Results

**Fig. 2:**
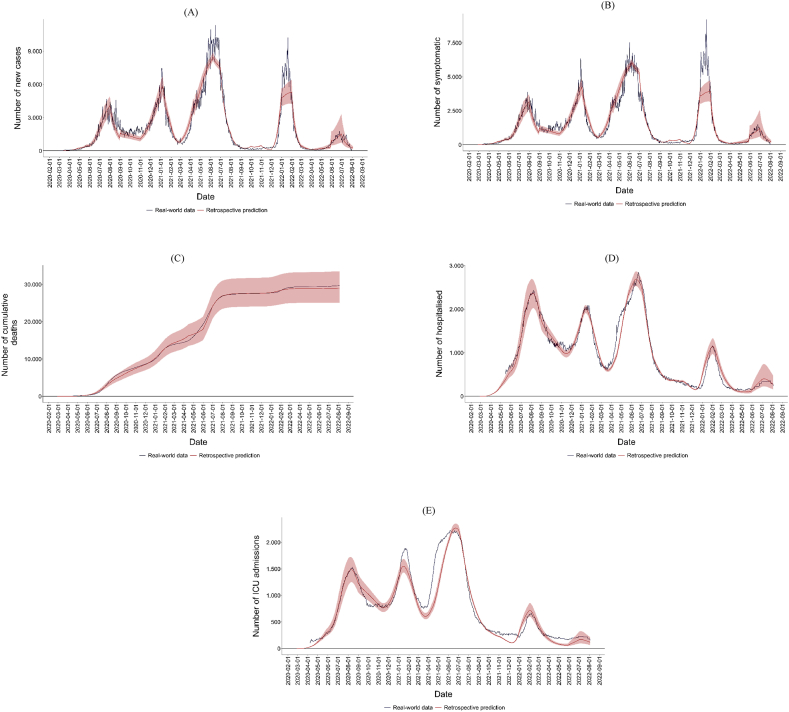
Retrospective predictions for health outcomes of SARS-CoV-2 in Bogotá. (A) Number of new cases, (B) Number of symptomatic patients, (C) Number of cumulative deaths, (D) Number of hospitalised patients, (E) Number of patients admitted to ICU.

The selection of the best model is based on standardised goodness-of-fit measures using a min-max transformation of the Mean Square Error (MSE), the MAE, and Theil's U statistic. After several iterations and calibrations, the selected model achieved an MSE of 0.02, an MAE of 0.16 and a Theil's U of 0.73. Fig. 2(A–E) presents the model fits to the time series of various health outcomes, demonstrating good performance according to real-world data.

**Fig. 3:**
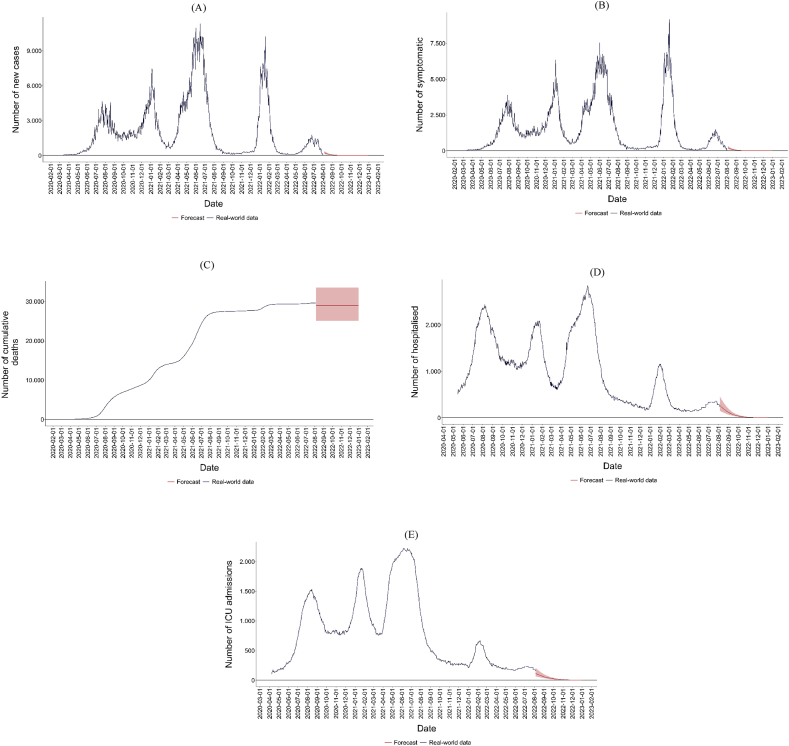
Predicted health outcomes of SARS-CoV-2 in Bogotá. (A) Number of new cases, (B) Number of symptomatic patients, (C) Number of cumulative deaths, (D) Number of hospitalised patients, (E) Number of patients admitted to ICU. *Note.* The interval shaded in red refers to the interquartile range of the forecast.

Fig. 3(A–E) presents the forecast for different health outcomes from 5 August to 31 December 2022, indicating a general decline. This decrease is attributed to various factors and measures implemented previously. It also reflects the immunity acquired by much of the community, either naturally or through vaccination, and the presence of less aggressive variants currently circulating in the city.

The projected incidence of SARS-CoV-2 is lower compared to earlier stages of the pandemic, as is the mortality associated with the virus. The model reflects a significant decrease in the number of new and symptomatic cases, which stabilised after the peak experienced by the city in mid-2022. With reduced virus transmission in the population, the demand for hospitalisation services logically decreases, as evidenced by the sharp decline in the number of patients requiring general hospitalisation and intensive care. Finally, due to the reduced severity of symptomatic infections, mortality associated with COVID-19 is more effectively controlled.

### 4.1 Sensitivity analysis

Sensitivity analysis examines how variations in certain model parameters affect the results for the different health outcomes. In the present study, four macro-scenarios are simulated with variations in the efficacy parameter (and, therefore, in vaccine effectiveness) $\left(vac_{eff}\right)$, vaccine efficacy in previously infected persons $\left(vac_{effr}\right)$, transmissibility (pm(t)), post-vaccination infection ($\sigma_{Rmod}\left(t\right))$, protection (coverage) by the use of surgical masks $\left(m_{cov}\left(t\right)\right)$, and physical distancing $\left(d_{cov}\left(t\right)\right)$. Each simulated scenario is described in Table 1.

**Table 1:** Sensitivity analysis of parameters in a model for SARS-CoV-2 in the city of Bogotá.

Scenarios		$vac_{eff}$	$vac_{effr}$	pm(t)	$\sigma_{Rmod}\left(t\right)$	$m_{cov}\left(t\right)$	$d_{cov}\left(t\right)$
Base		Calibrated	Calibrated	0.2 (base)	10 % (base)	10 % (base)	0 % (base)
Low efficacy (and effectiveness) of the vaccine, relaxation of non-pharmacological measures	1A	50	60	0.8	10 % (base)	10 % (base)	0 % (base)
	1B	55	60	0.7	15 %	15 %	5 %
	1C	60	65	0.8	10 % (base)	10 % (base)	0 % (base)
	1D	60	65	0.7	15 %	15 %	5 %
Low efficacy (and effectiveness) of the vaccine, moderate non-pharmacological measures	2A	50	60	1.2	25 %	55 %	30 %
	2B	55	65	1.3	30 %	60 %	35 %
	2C	50	65	1.2	25 %	55 %	30 %
	2D	55	60	1.3	30 %	60 %	35 %
High efficacy (and effectiveness) of the vaccine, relaxation of non-pharmacological measures	3A	80	90	0.8	10 % (base)	10 % (base)	0 % (base)
	3B	85	95	0.7	15 %	15 %	5 %
	3C	80	90	0.6	10 % (base)	10 % (base)	0 % (base)
	3D	85	95	0.7	15 %	15 %	5 %
High efficacy (and effectiveness) of the vaccine, strict non-pharmacological measures	4A	80	90	1.35	40 %	80 %	50 %
	4B	85	90	1.4	50 %	80 %	60 %
	4C	90	95	1.35	40 %	80 %	50 %
	4D	95	98	1.4	50 %	80 %	65 %

The forecast figures for each health outcome across the different scenarios are presented in Supplement 3, with the corresponding values detailed in Supplement 4. In the first scenario, which involves decreased vaccine efficacy, increased transmissibility, and relaxation of non-pharmacological measures, the results suggest that the fifth wave of the virus would extend until November 2022 or even result in a new, smaller wave by the end of 2023 due to the transmissibility of variants. As anticipated, this scenario indicates an increase in patients requiring general hospitalisation and ICU care, as well as a rise in COVID-19-related deaths. Conversely, the most favourable health outcomes are achieved when there is high vaccine effectiveness combined with strict non-pharmacological measures imposed on the population.

Notably, modifying vaccine efficacy (effectiveness) has a greater impact on health outcomes than changes in non-pharmacological measures. However, the optimal health outcome for the population is achieved by combining pharmacological and non-pharmacological interventions, even when considering transmissible variants. These results are consistent across different scenarios, whether simulating one, two, three, or four waves, demonstrating the robustness of the mathematical model. Nonetheless, the onset of each wave was significantly influenced by the timing, magnitude, and adherence to non-pharmaceutical interventions, such as the substantial relaxation of measures at the beginning of 2021.

## 5 Discussion

Our novel mathematical approach, which utilises real-world evidence of epidemiological behaviour in Bogotá, suggests a low probability of a sixth wave, except in scenarios where the circulation of new variants significantly reduces vaccine effectiveness and non-pharmacological measures are relaxed.

Modelling the SARS-CoV-2 pandemic worldwide poses a significant challenge for researchers due to its novelty and the tools available to understand its dynamics. Although the literature encompasses a wide array of approaches, including agent-based modelling, various variants of the compartmental model first proposed by Kermack et al. [57], and machine learning models, among others, selecting the appropriate analytical approach remains complex due to the specificities of each region or scenario. Balancing the need to incorporate as many real-world elements as possible while maintaining simplicity, parsimony, and computational efficiency in the analysis is an ongoing challenge.

Consequently, the approach proposed in this study reflects the characteristics of the virus in Bogotá while incorporating the various pharmacological and non-pharmacological measures implemented by the authorities since the beginning of 2020. By using an integrative model and conducting various sensitivity analyses with real-world data and parameters extracted from scientific literature, this study exemplifies the implications for incidence, lethality, and hospital burden of the virus across different epidemiological scenarios and interventions.

According to the findings of this research, the period of large waves of contagion and the associated pressure on the healthcare system appears to have subsided. If the evolution of SARS-CoV-2 remains relatively constant and there are no changes in the vaccine's effectiveness, transmissibility, and severity of the virus, new infections and hospitalisations will be within a manageable range for the authorities, minimising mortality in cases that typically present complications. This stability results from a complex interplay of vaccination coverage in the city, natural immunity from infection, and hybrid immunity from infection and vaccination [58]. However, if the effectiveness of vaccines and non-pharmacological measures declines, and more transmissible or virulent variants are introduced, a sixth wave with significant numbers of COVID-19 cases, hospitalisations, and deaths is likely to occur.

It should be noted that, unfortunately, immunity to infection declines over time. One way to maintain vaccine effectiveness over a long period is to administer booster doses, especially in the at-risk population. However, the frequency and number of doses required to sustain this immunity is unclear. Similarly, it is uncertain whether the decline in immunity affects mortality to the same extent as the risk of infection, and whether any specific population is at greater risk due to a decrease in “anti-infectious” immunity. Taking into account the higher risk groups (over 70 or 80 years of age with chronic diseases and comorbidities) and individuals with a poor response to vaccination, there could be a population group of approximately 1 % of adults at high risk of hospitalisation and complications [59].

In this context, it is worthwhile for health decision-makers to utilise tools such as the proposed predictive model, alongside other inputs, and to constantly monitor indicators related to the epidemic evolution of the SARS-CoV-2 virus. This approach enables discrete action on warning signs and helps to minimise potential new impacts on the well-being of the district's population. It is crucial to maintain surveillance of at-risk populations (usually related to high cost, such as dialysis patients, transplant recipients, and individuals with haematological tumours) and to ensure the availability of medicines and other strategies for the early infection management and the prevention of future complications.

The results of our analysis should be interpreted with caution due to the technical limitations of the mathematical epidemiological model [60,61]. First, we considered the effectiveness of vaccines aggregated using a dose-weighted average in Bogotá; however, there are differences in effectiveness against infection, symptomatic disease, and severe disease that have been reported among the elderly population in our setting [62]. Additionally, our model does not account for comorbidities that individuals in the city may have, nor their sex, race, occupation, or socioeconomic background. Therefore, vaccination stages cannot be captured accurately. However, this shortcoming is mitigated by proper calibration of the parameters, resulting in a good model fit and an expected forecast given the stage of the pandemic. Another limitation is the lack of city-specific information on some parameters that characterise the dynamics of the virus. In these cases, the best available evidence from the international literature is utilised.

These limitations present an opportunity for improvement in future work. We believe that disaggregating vaccine effectiveness can provide valuable insights into the differential evolution of individuals when exposed to or infected by the SARS-CoV-2 virus. Additionally, incorporating characteristics such as occupation, comorbidities, and socioeconomic background can add value to welfare analyses and aid in understanding the impact of infection on different population groups, beyond the health outcomes assessed in this study.

Finally, several conclusions are drawn from the findings obtained through the CoMo model, calibrated with real-world data for Bogotá. Firstly, the model in its central scenario does not predict another wave of SARS-CoV-2 in the city, indicating a downward stability in incidence, hospitalisation, and mortality. Secondly, for the first time in the international literature, our analytical modelling successfully incorporates the possibility of variants within the CoMo Consortium structure. This rigorous mathematical derivation represents an important innovation in the study, providing a valuable tool for future research. Thirdly, the increased transmissibility of variants with a greater capacity to evade immunity induced by current vaccines, combined with the relaxation of non-pharmacological protection measures, could lead the city to a new wave of infections. In such scenario, the response by the authorities and adherence to non-pharmacological measures would be crucial. Finally, due to its fit, versatility, and attention to detail, this quantitative study can serve as a basis for evidence-based decision-making regarding the SARS-CoV-2 pandemic or can be modified to study other infectious diseases of interest.

## CRediT authorship contribution statement

**Oscar Espinosa:** Writing – review & editing, Writing – original draft, Visualization, Validation, Supervision, Software, Resources, Project administration, Methodology, Investigation, Funding acquisition, Formal analysis, Data curation, Conceptualization. **Lisa White:** Writing – review & editing, Writing – original draft, Visualization, Validation, Software, Methodology, Investigation, Formal analysis. **Valeria Bejarano:** Writing – review & editing, Writing – original draft, Visualization, Validation, Software, Methodology, Investigation, Formal analysis, Data curation. **Ricardo Aguas:** Writing – review & editing, Writing – original draft, Visualization, Validation, Software, Methodology, Investigation, Formal analysis. **Duván Rincón:** Writing – review & editing, Writing – original draft, Visualization, Validation, Software, Methodology, Investigation, Formal analysis, Data curation. **Laura Mora:** Writing – review & editing, Writing – original draft, Visualization, Validation, Methodology, Investigation, Data curation. **Antonio Ramos:** Writing – review & editing, Writing – original draft, Visualization, Validation, Software, Methodology, Investigation, Formal analysis, Data curation. **Cristian Sanabria:** Writing – review & editing, Writing – original draft, Visualization, Validation, Software, Methodology, Investigation, Formal analysis, Data curation. **Jhonathan Rodríguez:** Writing – review & editing, Writing – original draft, Visualization, Validation, Software, Methodology, Investigation, Formal analysis, Data curation. **Nicolás Barrera:** Writing – review & editing, Writing – original draft, Visualization, Validation, Software, Methodology, Investigation, Formal analysis, Data curation. **Carlos Álvarez-Moreno:** Writing – review & editing, Writing – original draft, Validation. **Jorge Cortés:** Writing – review & editing, Writing – original draft, Validation. **Carlos Saavedra:** Writing – review & editing, Writing – original draft, Validation. **Adriana Robayo:** Writing – review & editing, Writing – original draft, Validation, Resources, Funding acquisition. **Bo Gao:** Writing – original draft, Software. **Oscar Franco:** Writing – review & editing, Writing – original draft, Validation.

## Data availability

Data and programming code will be made available on request to the corresponding author.

## Ethical approval statement

Not applicable since data from a secondary source was used.

## Funding source

Pan-American Health Organization (10.13039/100011893PAHO) funded this study through the Contract CON22-00000261 with the Instituto de Evaluación Tecnológica en Salud (IETS), with the support of Koica and the District Health Secretariat of Bogotá.

## Declaration of competing interest

The authors declare that they have no known competing financial interests or personal relationships that could have appeared to influence the work reported in this paper.

## Footnotes

Appendix A Supplementary data to this article can be found online at https://doi.org/10.1016/j.heliyon.2024.e39725.

## Footnotes

1 Colombia has one of the most complete health information systems in the world for COVID-19 statistics. It has data disaggregated by sex, simple age, geographical area, among other characteristics, and is open access for anyone who wishes to analyse it (https://www.ins.gov.co/Noticias/Paginas/Coronavirus.aspx).
