# Supplementary material for: Predictive modelling of the effectiveness of vaccines against COVID-19 in Bogotá: Methodological innovation involving different variants and computational optimisation efficiency
Source: Heliyon. 2024 Oct 23;10(21):e39725. doi: 10.1016/j.heliyon.2024.e39725 (PMC11570482; doi:10.1016/j.heliyon.2024.e39725)
Supplement: Multimedia component 1 [file mmc1.docx]

Supplement 1. Mathematical development of the epidemiological model

1. Table of parameters

| Parameter | Code name | Description | Initial value/type | Source |
| --- | --- | --- | --- | --- |
| $startdate$ | date_range_simul_start | Date range of simulation – Start. | 27/02/2020 | Instituto Nacional de Salud (2020a) |
| $stopdate$ | date_range_simul_end | Date range of simulation – End. | 04/08/2022 | Authors' criteria |
| $init$ | init | Number of exposed people at start date. | 1.6928 | Prem et al. (2017) |
| $pre$ | pre | Proportion of population with partial immunity at the start date. | 0% | - |
| $p$ | p | Probability of infection given contact. | 0.115 | Guzmán et al. (2022) |
| $r$ | report | Percentage of all asymptomatic infections that are reported. | 20% | Yanes-Lane et al. (2020) |
| $r_{c}$ | reportc | Percentage of all symptomatic infections that are reported. | 14% | Laajaj et al. (2021) |
| $r_{g}$ | reporth_g | Percentage of denied hospitalisations that are reported. | 41% | Czeisler et al. (2022) |
| $r_{h}$ | reporth | Percentage of non-severe hospitalisations that are appropriately treated. | 90% | CoMo Consortium (2022) |
| $r_{hICU}$ | reporth_ICU | Percentage of severe hospitalisations that are appropriately treated. | 90% | CoMo Consortium (2022) |
| $r_{v}$ | report_v | Percentage of all asymptomatic infections in previously vaccinated people that are reported. | 16% | North et al. (2022) |
| $r_{vr}$ | report_vr | Percentage of all asymptomatic infections in previously vaccinated and exposed people that are reported. | 16% | North et al. (2022) |
| $r_{r}$ | report_r | Percentage of all asymptomatic infections in previously infected people that are reported. | 18.9% | Ren et al. (2022) |
| $r_{cv}$ | report_cv | Percentage of all symptomatic infections in previously vaccinated people that are reported. | 30.1% | Ministerio de Salud y Protección Social (2021) |
| $r_{cvr}$ | report_cvr | Percentage of all symptomatic infections in previously vaccinated and exposed people that are reported. | 30.1% | Ministerio de Salud y Protección Social (2021) |
| $r_{cr}$ | report_cr | Percentage of all symptomatic infections in previously infected people that are reported. | 5.9% | Sheehan et al. (2021) |
| $r_{ndI}$ | report_natdeathI | Percentage of all people dying outside the hospital with asymptomatic infections reported as covid-deaths. | 27% | El Tiempo (2020) |
| $r_{ndCL}$ | report_natdeathCL | Percentage of all people dying outside the hospital with symptomatic infections reported as covid-deaths. | 27% | El Tiempo (2020) |
| $r_{dHC}$ | report_death_HC | Percentage of all people dying outside the hospital with severe infections reported as covid-deaths. | 27% | El Tiempo (2020) |
| - | iterations | Iterations (1 to 10,000). | 300 | Authors' criteria |
| - | noise | Noise (0.01 to 0.2). | 0.1 | Authors' criteria |
| - | confidence | Confidence (5 to 25). | 5% | Authors' criteria |
| - | sample_size | Average sample size for seroprevalence. | 42,164 | Laajaj et al. (2021) |
|  | | | | |
| Country parameters | | | | |
| $hs$ | household_size | Mean household size. | 2.758 | Departamento Administrativo Nacional de Estadística (2020b, 2021) |
| $imp$ | mean_imports | Mean number of infectious migrants per day. | 2.143 | Departamento Administrativo Nacional de Estadística (2020a) |
| $\boldsymbol{W}_{\boldsymbol{h}}$ | contact_home | Country-specific age-dependent contact matrix describing the number of potentially infectious contacts at home per person per day. | Matrix  $21 \times21$ | Prem et al. (2017) |
| $\boldsymbol{W}_{\boldsymbol{o}}$ | contact_other | Country-specific age-dependent contact matrix describing the number of potentially infectious societal contacts per person per day. | Matrix  $21 \times21$ | Prem et al. (2017) |
| $\boldsymbol{W}_{\boldsymbol{s}}$ | contact_school | Country-specific age-dependent contact matrix describing the number of potentially infectious contacts at school per person per day. | Matrix  $21 \times21$ | Prem et al. (2017) |
| $\boldsymbol{W}_{\boldsymbol{w}}$ | contact_work | Country-specific age-dependent contact matrix describing the number of potentially infectious contacts at work per person per day. | Matrix  $21 \times21$ | Prem et al. (2017) |
| $\boldsymbol{\mu}$ | mort_col | Age-dependent mortality rate. | Vector size number of times | Departamento Administrativo Nacional de Estadística (2022a, 2022b, 2022c) |
| $\boldsymbol{B}$ | popbirth_col2 | Population of births dependent on age. | Vector size $21$ | Departamento Administrativo Nacional de Estadística (2022b, 2022e, 2022f) |
| $\boldsymbol{N}$ | popstruc_col2 | Total population dependent on age. | Vector size $21$ | Departamento Administrativo Nacional de Estadística (2021) |
| $\boldsymbol{ihr}$ | ihr_col2 | Infection hospitalisation rate. | Vector size $21$ | Instituto Nacional de Salud (2020c) |
| $\boldsymbol{ifr}$ | ifr_col2 | Infection fatality rate. | Vector size $21$ | Instituto Nacional de Salud (2020c) |
|  | | | | |
| Virus parameters | | | | |
| $\rho$ | rho | Relative infectiousness of incubation phase. | 6.4% | World Health Organization (2020) |
| $\frac{1}{\gamma}$ | gamma | Average incubation period (1 to 7 days). | 5.680 | Khalili et al. (2020) |
| $\frac{1}{\nu}$ | nui | Average duration of symptomatic infection period (1 to 7 days). | 7 | He et al. (2020) |
| $\phi$ | phi | Month of peak infectivity of the virus (1, 2, …, 12). | 7 | SaluData (2022) |
| $\zeta$ | amp | Annual variation in infectivity of the virus. | 42.1% | Laajaj et al. (2021) |
| $\frac{1}{\omega}$ | omega | Average duration of immunity (0.5 to 150 years). | 0.583 | Center for Disease Control and Prevention (2021) |
| $p_{c}$ | pclin | Probability upon infection of developing clinical symptoms. | 58% | Larsen et al. (2021) |
| $p_{icu}$ | prob_icu | Probability upon hospitalisation of requiring Intensive Care Units (ICU) admission. | 25% | CoMo Consortium (2022) |
| $p_{v}$ | prob_vent | Probability upon admission to the ICU of requiring a ventilator. | 70% | Rodriguez et al. (2022) |
| $p_{ho2}$ | propo2 | Proportion of hospitalised patients needing O2. | 83.450% | Rodriguez et al. (2022) |
|  | | | | |
| Hospitalisation parameters | | | | |
| $b_{a}$ | beds_available | Maximum number of hospital surge beds. | 2,937 | SaluData (2022b) |
| $b_{icua}$ | icu_beds_available | Maximum number of ICU beds without ventilators. | 2,628 | SaluData (2022c) |
| $v_{a}$ | ventilators_available | Maximum number of ICU beds with ventilators. | 2,262 | Datos Abiertos Bogotá (2022) |
| $\rho_{s}$ | rhos | Relative percentage of regular daily contacts when hospitalised. | 15% | Moldokmatova et al. (2020) |
| $ihr_{s}$ | ihr_scaling | Scaling factor for infection hospitalisation rate (0.1 to 5) | 1 | Clinical expert criteria |
| $p_{dh}$ | pdeath_h | Probability of dying when hospitalised (not req. O2). | 15% | CoMo Consortium (2022) |
| $p_{dho}$ | pdeath_ho | Probability of dying when hospitalised if req. O2. | 20% | CoMo Consortium (2022) |
| $p_{dhc}$ | pdeath_hc | Probability of dying when denied hospitalisation (not req. O2). | 20% | Moldokmatova et al. (2020) |
| $p_{dhco}$ | pdeath_hco | Probability of dying when denied hospitalisation if req. O2. | 40% | Moldokmatova et al. (2020) |
| $p_{dicu}$ | pdeath_icu | Probability of dying when admitted to ICU (not req. O2). | 30% | Instituto Nacional de Salud (2020, 2021, 2022) |
| $p_{dicuo}$ | pdeath_icuo | Probability of dying when admitted to ICU if req. O2. | 40% | Instituto Nacional de Salud (2020, 2021, 2022) |
| $p_{dicuc}$ | pdeath_icuc | Probability of dying when admission to ICU denied (not req. O2). | 70% | Moldokmatova et al. (2020) |
| $p_{dicuco}$ | pdeath_icuco | Probability of dying when admission to ICU denied if req. O2. | 75% | Moldokmatova et al. (2020) |
| $p_{dv}$ | pdeath_vent | Probability of dying when ventilated. | 67% | Rodriguez et al. (2022) |
| $p_{dvc}$ | pdeath_ventc | Probability of dying when ventilator denied. | 95% | Moldokmatova et al. (2020) |
| $p_{dvhc}$ | pdeath_vent_hc | Probability of dying when ventilator required and not going to hospital. | 90% | Moldokmatova et al. (2020) |
| $p_{dicuhc}$ | pdeath_icu_hc | Probability of dying when ICU required (not O2) and not going to hospital. | 90% | Moldokmatova et al. (2020) |
| $p_{dicuhco}$ | pdeath_icu_hco | Probability of dying when ICU required (req. O2) and not going to hospital. | 90% | Moldokmatova et al. (2020) |
| $\frac{1}{\nu_{s}}$ | nus | Duration of hospitalised infection (1 to 30 days). | 8 | Rodriguez et al. (2022) |
| $\frac{1}{\nu_{icu}}$ | nu_icu | Duration of ICU infection (1 to 30 days). | 15 | Rodriguez et al. (2022) |
| $\frac{1}{\nu_{v}}$ | nu_vent | Duration of ventilated infection (1 to 30 days). | 11 | Rodriguez et al. (2022) |
| $\frac{1}{\nu_{icuc}}$ | nu_icuc | Duration of infection in the ICU that are not adequately treated (1 to 30 days). | Numeric | Gao (2021);  Celhay (2021) |
| $\frac{1}{\nu_{vc}}$ | nu_ventc | Duration of infection with ventilator that is not adequately treated (1 to 30 days). | Numeric | Gao (2021);  Celhay (2021) |
| $\frac{1}{\nu_{sc}}$ | nusc | Duration of hospitalised infection not adequately treated (1 to 30 days). | Numeric | Gao (2021);  Celhay (2021) |
|  | | | | |
| Vaccination parameters | | | | |
| $p_{cv}$ | pclin_v | Probability upon infection of developing clinical symptoms if previously vaccinated. | 23.7% | Ayoubkhani et al. (2022) |
| $p_{cvr}$ | pclin_vr | Probability upon infection of developing clinical symptoms if previously vaccinated and exposed. | 23.7% | Ayoubkhani et al. (2022) |
| $p_{cr}$ | pclin_r | Probability upon infection of developing clinical symptoms if previously infected. | 58% | Larsen et al. (2021) |
| $p_{icuv}$ | prob_icu_v | Probability upon hospitalisation of requiring ICU admission if previously vaccinated. | 25% | CoMo Consortium (2022) |
| $p_{icuvr}$ | prob_icu_vr | Probability upon hospitalisation of requiring ICU admission if previously vaccinated and exposed. | 25% | CoMo Consortium (2022) |
| $p_{icur}$ | prob_icu_r | Probability upon hospitalisation of requiring ICU admission if previously infected. | 25% | CoMo Consortium (2022) |
| $p_{vv}$ | prob_v_v | Probability upon admission to the ICU of requiring a ventilator if previously vaccinated. | 70% | Rodriguez Lima et al. (2022) |
| $p_{vvr}$ | prob_v_vr | Probability upon admission to the ICU of requiring a ventilator if previously vaccinated and exposed. | 70% | Rodriguez Lima et al. (2022) |
| $p_{vr}$ | prob_v_r | Probability upon admission to the ICU of requiring a ventilator if previously infected. | 70% | Rodriguez Lima et al. (2022) |
| $\sigma_{R}$ | sigmaR | Probability of infection of people that have recovered from a previous infection. | 37.2% | Centers for Disease Control and Prevention (2022) |
| $\sigma_{EV}$ | sigmaEV | Probability of requiring hospitalisation if previously vaccinated. | 23% | CoMo Consortium (2022) |
| $\sigma_{ER}$ | sigmaER | Probability of requiring hospitalisation if previously infected. | 23% | CoMo Consortium (2022) |
| $\sigma_{EVR}$ | sigmaEVR | Probability of requiring hospitalisation if previously infected and vaccinated. | 23% | CoMo Consortium (2022) |
| $\frac{1}{sg}$ | seroneg | Days from seropositive to seronegative. | 300 | Alfego et al. (2021) |
| $vac_{c}$ | vac_campaign | Vaccination - Time to reach target coverage (1 to 52 weeks). | 39 | Alcaldía de Bogotá D.C. (2021a , 2021b) |
| $\frac{1}{vac_{d}}$ | vac_dur | Vaccination - Duration of efficacious period (years). | 0.5 | Ministerio de Salud y Protección Social (2021) |
| $\frac{1}{vac_{dr}}$ | vac_dur_r | Vaccination - Duration of efficacious period if previously infected (years). | 0.5 | Hall et al. (2022) |
| $vac_{eff}$ | vaccine_eff | Vaccination - Efficacy against infection. | 69.866% | Gardner & Kilpatrick (2021); Higdon et al. (2022); Nasreen et al. (2022); Pormohammad et al. (2022); Reza et al. (2022) |
| $vac_{effr}$ | vaccine_eff_r | Vaccination - Efficacy against infection if previously infected. | 69.866% | Gardner & Kilpatrick (2021); Higdon et al. (2022); Nasreen et al. (2022); Pormohammad et al. (2022); Reza et al. (2022) |
|  | | | | |
| Intervention parameters | | | | |
| $self_{eff}$ | selfis_eff | Self-isolation if symptomatic (adherence). I.e., estimated reduction in contact due to self-isolation if symptomatic. | 20.2% | Smith et al. (2021) |
| $s_{over}$ | screen_overdispersion | Overdispersion: (1, 2, 3, 4 or 5). | 1 | Endo et al. (2020) |
| $s_{tests}$ | screen_test_sens | Test sensitivity. | 89.479% | BIO-TEST IPS S.A.S. (2020); Clínica Universitaria Medicina Integral S.A.S (2020); Gutierrez et al., (2021); Instituto Nacional de Salud (2020a, 2020c); Laboratorio Clínico Alife Health (2020); Laboratorio de salud pública departamental del Valle (2020); Universidad el Bosque (2020) |
| $qd$ | quarantine_days | Days in isolation for average person. | 8 | Raslan (2021) |
| $\frac{1}{qe}$ | quarantine_effort | Days to implement maximum quarantine coverage (1 to 5). | 5 | Gobierno Nacional de Colombia (2020) |
| $q_{effo}$ | quarantine_eff_other | Decrease in the number of other contacts when quarantined. | 0.070% | Prem et al. (2017, 2021) |
| $q_{effh}$ | quarantine_eff_home | Increase in the number of contacts at home when quarantined. | -27% | Lei et al. (2020) |
| $d_{eff}$ | dist_eff | Social distancing (adherence). That is, reduction of contacts in the community between those who adhere to social distancing. | 96.2% | González et al. (2021) |
| $h_{eff}$ | hand_eff | Efficacy of hand washing (0-25%). | 21% | Centers for Disease Control and Prevention (2020) |
| $m_{eff}$ | mask_eff | Efficacy of the use of masks (0-35%).  Estimated reduction of contact due to mask use. | 51% | Lindsley et al. (2021) |
| $w_{eff}$ | work_eff | Work at home (efficiency). I.e., reduction of contacts at work among those who adhere to work-from-home policies. | 87% | Zalat & Bolbol (2022) |
| $w_{2h}$ | w2h | Home contacts inflation due to working from home. | 27% | Lei et al. (2020) |
| $s_{2h}$ | s2h | Home contacts inflation due to school closure. | 13% | Australian Bureau of Statistics (2022) |
| $coc_{eff}$ | cocoon_eff | Shielding the elderly (efficacy). That is, the reduction of contacts in all settings when isolation is applied in older adults. | 50% | Tran et al. (2021) |
| $a_{coc}$ | age_cocoon | Minimum age for elderly shielding (0 to 100 years). | 70 | Gobierno Nacional de Colombia (2020b) |
| $m_{tests}$ | mass_test_sens | Mass testing (sensitivity). | 89.479% | Instituto Nacional de Salud (2022c, 2022ag) |
| $id$ | isolation_days | Isolation days. | 14 | Lauer et al. (2020) |
| $d_{2}$ | dexo2 | Relative risk of dying if needing O2 and taking dexamethasone*.* | 82% | RECOVERY Collaborative Group (2021) |
| $d_{v}$ | dexv | Relative risk of dying if needing ventilation and taking dexamethasone*.* | 64% | RECOVERY Collaborative Group (2021) |
| $d_{2c}$ | dexo2c | Relative risk of dying if needing but not receiving O2 and taking dexamethasone*.* | 81% | RECOVERY Collaborative Group (2021) |
| $d_{vc}$ | dexvc | Relative risk of dying if needing but not receiving ventilation and taking dexamethasone*.* | 82% | RECOVERY Collaborative Group (2021) |
| $v_{d}$ | vent_dex | Change in ventilation requirement if given dexamethasone*.* | 79% | RECOVERY Collaborative Group (2021) |

**Some functions of parameters**

| **Notation** | **Code name** | **Description** | **Type** |
| --- | --- | --- | --- |
| **Force of infection** | | | |
| $\boldsymbol{\lambda}(t)$ | lam | Force of infection. | Vector function size $21$ |
| $\boldsymbol{\varrho}(t)$ | lamq | Force of infection, considering contacts in home isolation. | Vector function size $21$ |
|  | | | |
| **Population** | | | |
| $\boldsymbol{Ag}$ | ageing | Speed of population ageing. | Matrix  $21 \times21$ |
| $\boldsymbol{b}$ | birth | Number of births. | Vector size $21$ |
|  | | | |
| **Test rates** | | | |
| $rt_{C}(t)$ | ratetestC | The test rate depends on whether the population is greater than 1 at a specific time. Otherwise, it will be zero. | Numeric function |
| $rt_{E}(t)$ | ratetestE |  |  |
| $rt_{ER}(t)$ | ratetestER |  |  |
| $rt_{EV}(t)$ | ratetestEV |  |  |
| $rt_{EVR}(t)$ | ratetestEVR |  |  |
| $rt_{HC}(t)$ | ratetestHC |  |  |
| $rt_{HCICU}(t)$ | ratetestHCICU |  |  |
| $rt_{HCV}(t)$ | ratetestHCV |  |  |
| $rt_{I}(t)$ | ratetestI |  |  |
|  | | | |
| **Available beds** | | | |
| $c$ | crit | Proportion of hospitalised people requiring ICU beds or ventilators that do not receive one. | Numeric |
| $c_{H}$ | critH | Proportion of individuals requiring hospitalisation that do not receive a surge bed. | Numeric |
| $c_{V}$ | critV | Proportion of people hospitalised in ICU who require ventilators that do not receive one. | Numeric |
|  | | | |
| **Interventions** | | | |
| $\boldsymbol{1}_{\boldsymbol{test}}(t)$ | age_testing_vector | Age-indicator tests apply for a specific time. | Vector function size$21$ |
| $\boldsymbol{1}_{\boldsymbol{vac}}(t)$ | age_vaccine_vector | Age-indicator vaccine apply for a specific time. | Vector function size$21$ |
| $\boldsymbol{1}_{\boldsymbol{scc}}(t)$ | schoolclose2 | Age-indicator school closure apply for a specific time. | Vector function size$21$ |
| $\boldsymbol{1}_{\boldsymbol{sccp}}(t)$ | schoolclose2p | Age-indicator partial school closure apply for a specific time. | Vector function size$21$ |
| $coc(t)$ | cocoon | The function relates the time and the value corresponding to coverage of protected elderly. | Numeric function |
| $\sigma_{Rmod}(t)$ | cmod_vector | The function relates the time and the value corresponding to breakthrough infection probability. | Numeric function |
| $dm(t)$ | dmod_vector | The function relates the time and the value corresponding to the lethality of the variant. | Numeric function |
| $pm(t)$ | pmod_vector | The function relates the time and the value corresponding to the transmissibility of the variant. | Numeric function |
| $d_{x}(t)$ | dex | The function indicates the time instant at which dexamethasone was applied. | Numeric function |
| $d_{cov}(t)$ | dist_cov | The function relates the time and the value corresponding to the adherence to social distancing at the community level. | Numeric function |
| $d(t)$ | distancing | The function indicates the time instant at which social distancing was applied. | Numeric function |
| $\boldsymbol{d}_{\boldsymbol{2}\boldsymbol{h}}$ | dexo2_hist | The vector takes the value 1 or the value of parameter $d_{2}$ for all the times according to dexamethasone intervention. | Vector size number of times |
| $\boldsymbol{d}_{\boldsymbol{2}\boldsymbol{ch}}$ | dexo2c_hist | According to the dexamethasone intervention, the vector takes the value 1 or the value of parameter $d_{2c}$ for all times. | Vector size number of times |
| $\boldsymbol{d}_{\boldsymbol{vh}}$ | dexv_hist | According to the dexamethasone intervention, the vector takes the value 1 or the value of parameter $d_{2v}$ for all times. | Vector size number of times |
| $\boldsymbol{d}_{\boldsymbol{vch}}$ | dexvc_hist | According to the dexamethasone intervention, the vector takes the value 1 or the value of parameter $d_{vc}$ for all times. | Vector size number of times |
| $h_{cov}(t)$ | hand_cov | The function relates the time and the value corresponding to intervention, such as hand washing coverage. | Numeric function |
| $m_{cov}(t)$ | mask_cov | The function relates the time and value corresponding to mask-wearing adherence. | Numeric function |
| $q(t)$ | quarantine | The function indicates the time instant at which household isolation was applied. | Numeric function |
| $q_{cov}(t)$ | quarantine_cov | The function relates the time and the value corresponding to the adherence to quarantine. | Numeric function |
| $qr(t)$ | quarantine_rate | Quarantine rate. | Numeric function |
| $sc_{effp}(t)$ | school_effp | The function relates the time and the value corresponding to reducing school contacts in case of partial school closure. | Numeric function |
| $s_{cr}(t)$ | screen_contacts | The function relates the time and the value corresponding to the number of contacts (self-isolation) screen. | Numeric function |
| $s_{eff}(t)$ | screen_eff | Efficacy of self-isolation if symptomatic. | Numeric function |
| $self(t)$ | selfis | The function relates the time and the value corresponding to the proportion of symptomatic individuals who self-isolate. | Numeric function |
| ${test}_{pd}(t)$ | tests_per_day | The function relates to time and the value corresponds to the number of people tested in one day, given in thousands. | Numeric function |
| $tb_{eff}(t)$ | travelban_eff | The function relates the time and the value corresponding to the efficacy of interrupting the flow of trips out of the city and isolating cases from abroad. | Numeric function |
| $vac_{cov}(t)$ | vaccine_cov | The function relates the time and the value corresponding to the adherence to vaccination. | Numeric function |
| $vac(t)$ | vaccinate | Vaccination rate. | Numeric function |
| $w_{cov}(t)$ | work_cov | The function relates the time and the value corresponding to the adherence to work-from-home policies. | Numeric function |
| $wh(t)$ | workhome | The function indicates the time instant at which working at home was applied. | Numeric function |

*Note.* The information in the columns "*Code name*" and "*Description*" is a combination of definitions (to maintain the coherence of the text), deductions, and authorship from Aguas et al. (2020), Diarra et al. (2022), Borges et al. (2021), Franco (2021), Franco et al. (2022) and Gao (2021).

B. Compartments (states) of the model

The dimension of each compartment at the final solution of the ordinary differential equations (ODE) is: number of times$\times21$.

| **Compartment symbol** | **Description** |
| --- | --- |
| S | Susceptible population. |
| SR | Susceptible population with prior exposure. |
| E | Population of infected and incubating (exposed). |
| I | Infectious and asymptomatic population after incubation. |
| CL | Infectious and mildly symptomatic population after incubation. |
| R | Recovered population. |
| X | Mildly symptomatic infected population self-isolating at home. |
| V | Vaccinated population. |
| EV | Exposed vaccinated population. |
| ER | Exposed recovered population. |
| VR | Vaccinated recovered population. |
| EVR | Population of recovered vaccinated exposed. |
| QS | Susceptible population in quarantine. |
| QE | Infected population in quarantine incubation period. |
| QSR | Recovered susceptible population in quarantine. |
| QI | Asymptomatic infected population in quarantine. |
| QC | Mildly symptomatic population in quarantine. |
| QR | Recovered population in quarantine. |
| QV | Vaccinated population in quarantine. |
| QEV | Exposed vaccinated population in quarantine. |
| QER | Recovered population exposed in quarantine. |
| QVR | Recovered population vaccinated in quarantine. |
| QEVR | Exposed and quarantined vaccinated recovered population. |
| H | Severe infection: hospitalised infected population. |
| HC | Severe infection: population not hospitalised due to lack of capacity. |
| HCICU | Severe infection: infected population requiring ICU but not hospitalised due to lack of capacity. |
| HCV | Severe infection: infected population requiring a ventilator but not hospitalised due to lack of capacity. |
| ICU | Severe infection: infected population hospitalised in ICU. |
| ICUC | Severe infection: hospitalised population requiring ICU but only receiving a surge bed. |
| ICUCV | Severe infection: hospitalised population requiring ICU and ventilator but only receiving a surge bed. |
| Vent | Severe infection: population hospitalised in the ICU and on a ventilator. |
| VentC | Severe infection: population hospitalised in the ICU that requires a ventilator but are not assigned one. |
| C | Reported cumulative cases. |
| CM | Reported cumulative deaths. |
| CMC | Accumulated cases of death of critical patients, those who were denied hospitalisation. |
| Z | Tests carried out in quarantine. |
| Ab | Proportion of the population who would test positive by serology. |

*Note.* The descriptions are a combination of definitions (to maintain the coherence of the text), deductions and authorship of Aguas et al. (2020), Diarra et al. (2022), Borges et al. (2021), Franco (2021), Franco et al. (2022) and Gao (2021). C. System of ordinary differential equations of the CoMo Consortium model

Vectors are represented in lowercase and matrices in uppercase, both in bold type. The operations addition ($+)$, subtraction $(-)$, multiplication ($\cdot$) and division ($/$) are used for scalars, vectors and matrices in the usual way. The operators $\boldsymbol{\odot}$ and $\mathbf{⊘}$ denote element-by-element multiplication and division, respectively, between vectors or between matrices and vectors. Throughout this section, we will treat the compartments as column vectors of size 21, corresponding to age groups, so that the derivate is computed for each component (age group) at each point in time.

Examples: i) Let $\boldsymbol{a}$ and $\boldsymbol{b}$ be vectors of dimension $1 x n$. The operation $\boldsymbol{a}\boldsymbol{\odot}\boldsymbol{b}$ is the element-by-element product, resulting in a vector of dimension $1 x n$; ii) Let $\boldsymbol{U}$ be a matrix of dimension $t x 21$ and $\boldsymbol{c}$ be a column vector of dimension $21 x 1$. The operation $\boldsymbol{U}\cdot\boldsymbol{c}$ results in a matrix of dimension $t x 1$; iii) Considering the matrix $\boldsymbol{U}$ and the vector $\boldsymbol{c}$ from ii) and changing the arithmetic operator to $\boldsymbol{\odot}$, that is, $\boldsymbol{U}\boldsymbol{\odot}\boldsymbol{c}$, results in a matrix of dimension $t x 21$. The vector of ones, represented by **1** (and $\boldsymbol{J}$ for matrix full of ones), has the appropriate dimensions for the operation.

| $\frac{d\boldsymbol{S}}{dt}=$ | $\boldsymbol{Ag\cdot S+}\left( \frac{1}{qd} \right)\boldsymbol{\cdot QS+}vac_{d}\boldsymbol{\cdot V+b-\mu\odot S-}qr\left( t \right)\boldsymbol{\cdot S-S\odot\lambda}\left( t \right)$  $\boldsymbol{-}vac\left( t \right)\boldsymbol{\cdot}\boldsymbol{1}_{\boldsymbol{vac}}\left( t \right)\boldsymbol{\odot S}$. | $\boldsymbol{(} SEQ Ecuación \backslash* ARABIC 1\boldsymbol{)}$ |
| --- | --- | --- |

| $\frac{d\boldsymbol{SR}}{dt} =$ | $\boldsymbol{Ag\cdot SR+}\omega\cdot\boldsymbol{R+}\left( \frac{1}{qd} \right)\boldsymbol{\cdot QSR-\mu\odot SR-SR\odot\lambda}(t)\boldsymbol{-}qr(t)\boldsymbol{\cdot SR}$. | $\boldsymbol{(} SEQ Ecuación \backslash* ARABIC 2\boldsymbol{)}$ |
| --- | --- | --- |

| $\frac{d\boldsymbol{E}}{dt} =$ | $\boldsymbol{S}\boldsymbol{\odot}\boldsymbol{\lambda}\left( t \right)\boldsymbol{+SR}\boldsymbol{\odot}\boldsymbol{\lambda}\left( t \right)\boldsymbol{+Ag\cdot E+}\left( \frac{1}{qd} \right)\boldsymbol{\cdot QE-}vac\left( t \right)\boldsymbol{\cdot}\boldsymbol{1}_{\boldsymbol{vac}}\left( t \right)\boldsymbol{\odot}\boldsymbol{E-\mu}\boldsymbol{\odot}\boldsymbol{E}$  $\boldsymbol{-}\gamma\cdot\boldsymbol{E-}qr(t)\boldsymbol{\cdot E.}$ | $\boldsymbol{(} SEQ Ecuación \backslash* ARABIC 3\boldsymbol{)}$ |
| --- | --- | --- |

| $\frac{d\boldsymbol{I}}{dt}=$ | $\gamma\cdot\left( 1-p_{c} \right)\cdot\left( 1-s_{eff}\left( t \right) \right)\cdot\left( \boldsymbol{1-ihr} \right)\boldsymbol{\odot}\left( \boldsymbol{1-}\boldsymbol{1}_{\boldsymbol{test}}\left( t \right)\boldsymbol{\cdot}rt_{E}\left( t \right) \right)\boldsymbol{\odot E}$  $\boldsymbol{+}\gamma\cdot\left( 1-p_{cv} \right)\cdot\left( 1-s_{eff}\left( t \right) \right)\boldsymbol{\cdot}\left( \boldsymbol{1-}\sigma_{EV} \cdot\boldsymbol{ihr} \right)\boldsymbol{\odot}\left( \boldsymbol{1-}\boldsymbol{1}_{\boldsymbol{test}}\left( t \right)\boldsymbol{\cdot}rt_{EV}\left( t \right) \right)\boldsymbol{\odot EV}$  $\boldsymbol{+}\gamma\cdot\left( 1-p_{cr} \right)\cdot\left( 1-s_{eff}\left( t \right) \right)\boldsymbol{\cdot}\left( \boldsymbol{1-}\sigma_{ER} \cdot\boldsymbol{ihr} \right)\boldsymbol{\odot}\left( \boldsymbol{1-}\boldsymbol{1}_{\boldsymbol{test}}\left( t \right)\boldsymbol{\cdot}rt_{ER}\left( t \right) \right)\boldsymbol{\odot ER}$  $\boldsymbol{+}\gamma\cdot\left( 1-p_{cvr} \right)\cdot\left( 1-s_{eff}\left( t \right) \right)\boldsymbol{\cdot}\left( \boldsymbol{1-}\sigma_{EVR} \cdot\boldsymbol{ihr} \right)\boldsymbol{\odot}\left( \boldsymbol{1-}\boldsymbol{1}_{\boldsymbol{test}}\left( t \right)\boldsymbol{\cdot}rt_{EVR}\left( t \right) \right)\boldsymbol{\odot EVR}$  $\boldsymbol{+}\left( \frac{1}{qd} \right)\boldsymbol{\cdot QI-}vac\left( t \right)\boldsymbol{\cdot}\boldsymbol{1}_{\boldsymbol{vac}}\left( t \right)\boldsymbol{\odot I-}\nu\boldsymbol{\cdot I+Ag\cdot I-\mu\odot I-}qr\left( t \right)\boldsymbol{\cdot I}$  $\boldsymbol{-}rt_{I}(t)\boldsymbol{\cdot}\boldsymbol{1}_{\boldsymbol{test}}(t)\boldsymbol{\odot I.}$ | $\boldsymbol{(} SEQ Ecuación \backslash* ARABIC 4\boldsymbol{)}$ |
| --- | --- | --- |

|  | $\frac{d\boldsymbol{CL}}{dt}\boldsymbol{=}$ | $\gamma\cdot p_{c}\cdot\left( \boldsymbol{1-}\boldsymbol{1}_{\boldsymbol{test}}\left( t \right)\cdot rt_{E}\left( t \right) \right)\boldsymbol{\odot}\left( 1-self\left( t \right) \right)\cdot\left( \boldsymbol{1-ihr} \right)\boldsymbol{\odot}\left( 1-qr\left( t \right) \right)\cdot\boldsymbol{E}$  $\boldsymbol{+}\gamma\cdot p_{cv}\cdot\left( \boldsymbol{1-}\boldsymbol{1}_{\boldsymbol{test}}\left( t \right)\cdot rt_{EV}\left( t \right) \right)\boldsymbol{\odot}\left( 1-self\left( t \right) \right)\cdot\left( \boldsymbol{1-}\sigma_{EV}\cdot\boldsymbol{ihr} \right)\boldsymbol{\odot}\left( 1-qr\left( t \right) \right)\cdot\boldsymbol{EV}$  $\boldsymbol{+}\gamma\cdot p_{cr} \cdot\left( \boldsymbol{1-}\boldsymbol{1}_{\boldsymbol{test}}\left( t \right)\cdot rt_{ER}\left( t \right) \right)\boldsymbol{\odot}\left( 1-self\left( t \right) \right)\cdot\left( \boldsymbol{1-}\sigma_{ER}\cdot\boldsymbol{ihr} \right)\boldsymbol{\odot}\left( 1-qr\left( t \right) \right)\cdot\boldsymbol{ER}$  $\boldsymbol{+}\gamma\cdot p_{cvr}\cdot\left( \boldsymbol{1-}\boldsymbol{1}_{\boldsymbol{test}}\left( t \right)\cdot rt_{EVR}\left( t \right) \right)\boldsymbol{\odot}\left( 1-self\left( t \right) \right)\cdot\left( \boldsymbol{1-}\sigma_{EVR}\cdot\boldsymbol{ihr} \right)\boldsymbol{\odot}\left( 1-qr\left( t \right) \right)\cdot\boldsymbol{EVR}$  $\boldsymbol{+ Ag}\cdot\boldsymbol{CL +}\left( \frac{1}{qd} \right)\cdot\boldsymbol{QC-}\nu\cdot\boldsymbol{CL- \mu}\boldsymbol{\odot}\boldsymbol{CL -}rt_{C}(t)\cdot\boldsymbol{1}_{\boldsymbol{test}}(t)\boldsymbol{\odot}\boldsymbol{CL}$. | $\boldsymbol{(} SEQ Ecuación \backslash* ARABIC 5\boldsymbol{)}$ |
| --- | --- | --- | --- |

|  | $\frac{d\boldsymbol{R}}{dt} =$ | $\nu\cdot\boldsymbol{I +}\nu\cdot\boldsymbol{X +}\nu\cdot\boldsymbol{CL + Ag}\cdot\boldsymbol{R +} \left( \frac{1}{id} \right)\cdot\boldsymbol{Z +}\left( \frac{1}{qd} \right)\cdot\boldsymbol{QR}$  $\boldsymbol{+}\nu_{s}\cdot p_{ho2}\cdot\left( 1\boldsymbol{-}d_{2}\cdot p_{dho}\cdot dm\left( t \right) \right)\cdot\boldsymbol{ifr\odot H}$  $\boldsymbol{+}\nu_{s}\cdot\left( 1-p_{ho2} \right)\cdot\left( 1\boldsymbol{-}p_{dh}\cdot dm\left( t \right) \right)\cdot\boldsymbol{ifr\odot H}$  $\boldsymbol{+}\nu_{sc}\cdot p_{ho2}\cdot\left( 1\boldsymbol{-}p_{dhco}\cdot dm\left( t \right) \right)\cdot\boldsymbol{ifr\odot HC}$  $\boldsymbol{+}\nu_{sc}\cdot\left( 1-p_{ho2} \right)\cdot\left( 1\boldsymbol{-}p_{dhc}\cdot dm\left( t \right) \right)\cdot\boldsymbol{ifr\odot HC}$  $\boldsymbol{+}\nu_{icu}\cdot p_{ho2}\cdot\left( 1\boldsymbol{-}d_{2}\cdot p_{dicuo}\cdot dm\left( t \right) \right)\cdot\boldsymbol{ifr\odot ICU}$  $\boldsymbol{+}\nu_{icu}\cdot\left( 1-p_{ho2} \right)\cdot\left( 1\boldsymbol{-}p_{dicu}\cdot dm\left( t \right) \right)\cdot\boldsymbol{ifr\odot ICU}$  $\boldsymbol{+}\nu_{icuc}\cdot p_{ho2}\cdot\left( 1\boldsymbol{-}d_{2c}\cdot p_{dicuco}\cdot dm\left( t \right) \right)\cdot\boldsymbol{ifr\odot ICUC}$  $\boldsymbol{+}\nu_{icuc}\cdot\left( 1-p_{ho2} \right)\cdot\left( 1\boldsymbol{-}p_{dicuc}\cdot dm\left( t \right) \right)\cdot\boldsymbol{ifr\odot ICUC}$  $\boldsymbol{+}\nu_{v}\cdot\left( 1\boldsymbol{-}d_{v}\cdot p_{dv}\cdot dm\left( t \right) \right)\cdot\boldsymbol{ifr\odot Vent}$  $\boldsymbol{+}\nu_{vc}\cdot\left( 1\boldsymbol{-}d_{vc}\cdot p_{dvc}\cdot dm\left( t \right) \right)\cdot\boldsymbol{ifr\odot VentC}$  $\boldsymbol{+}\nu_{vc}\cdot\left( 1\boldsymbol{-}d_{vc}\cdot p_{dvc}\cdot dm\left( t \right) \right)\cdot\boldsymbol{ifr\odot ICUCV +}vac_{dr}\cdot\boldsymbol{VR}$  $\boldsymbol{+}\nu_{sc}\cdot p_{ho2}\cdot\left( 1\boldsymbol{-}p_{dicuhco}\cdot dm\left( t \right) \right)\cdot\boldsymbol{ifr\odot HCICU}$  $\boldsymbol{+}\nu_{sc}\cdot\left( 1-p_{ho2} \right)\cdot\left( 1\boldsymbol{-}p_{dicuhc}\cdot dm\left( t \right) \right)\cdot\boldsymbol{ifr\odot HCICU}$  $\boldsymbol{+}\nu_{vc} \cdot\boldsymbol{(}1\boldsymbol{-}p_{dvhc}\cdot dm(t)\boldsymbol{)}\cdot\boldsymbol{ifr\odot HCV}$  $\boldsymbol{-}vac\left( t \right)\cdot\boldsymbol{1}_{\boldsymbol{vac}}\left( t \right)\boldsymbol{\odot R-\lambda}\left( t \right)\cdot\sigma_{Rmod}\left( t \right)\boldsymbol{\odot R-}qr\left( t \right)\cdot\boldsymbol{R-}\omega\cdot\boldsymbol{R -\mu\odot R}.$ | $\boldsymbol{(} SEQ Ecuación \backslash* ARABIC 6\boldsymbol{)}$ |
| --- | --- | --- | --- |

|  | $\frac{d\boldsymbol{X}}{dt}\boldsymbol{=}$ | $\left( \gamma\cdot self\left( t \right)\cdot\left( \boldsymbol{1-}\boldsymbol{1}_{\boldsymbol{test}}\left( t \right)\cdot rt_{E}\left( t \right) \right)\boldsymbol{\odot}p_{c}\cdot\left( \boldsymbol{1-ihr} \right) \right)\boldsymbol{\odot E}$  $\boldsymbol{+}\left( \gamma\cdot\left( 1-p_{c} \right)\cdot\left( \boldsymbol{1-}\boldsymbol{1}_{\boldsymbol{test}}\left( t \right)\cdot rt_{E}\left( t \right) \right)\boldsymbol{\odot}s_{eff}\left( t \right)\cdot\left( \boldsymbol{1-ihr} \right) \right)\boldsymbol{\odot E}$  $\boldsymbol{+}\gamma\cdot self\left( t \right)\cdot\left( \boldsymbol{1-}\boldsymbol{1}_{\boldsymbol{test}}\left( t \right)\cdot rt_{EV}\left( t \right) \right)\boldsymbol{\odot}p_{cv}\cdot\left( \boldsymbol{1-}\sigma_{EV}\cdot\boldsymbol{ihr} \right)\boldsymbol{\odot EV}$  $\boldsymbol{+}\gamma\cdot\left( 1-p_{cv} \right)\cdot\left( \boldsymbol{1-}\boldsymbol{1}_{\boldsymbol{test}}\left( t \right)\cdot rt_{EV}\left( t \right) \right)\boldsymbol{\odot}s_{eff}\left( t \right)\cdot\left( \boldsymbol{1-}\sigma_{EV}\cdot\boldsymbol{ihr} \right)\boldsymbol{\odot EV}$  $\boldsymbol{+}\gamma\cdot self\left( t \right)\cdot\left( \boldsymbol{1-}\boldsymbol{1}_{\boldsymbol{test}}\left( t \right)\cdot rt_{EVR}\left( t \right) \right)\boldsymbol{\odot}p_{cv}\cdot\left( \boldsymbol{1-}\sigma_{EVR}\cdot\boldsymbol{ihr} \right)\boldsymbol{\odot EVR}$  $\boldsymbol{+}\gamma\cdot\left( 1-p_{cvr} \right)\cdot\left( \boldsymbol{1-}\boldsymbol{1}_{\boldsymbol{test}}\left( t \right)\cdot rt_{EVR}\left( t \right) \right)\boldsymbol{\odot}s_{eff}\left( t \right)\cdot\left( \boldsymbol{1-}\sigma_{EVR}\cdot\boldsymbol{ihr} \right)\boldsymbol{\odot EVR}$  $\boldsymbol{+}\gamma\cdot self\left( t \right)\cdot\left( \boldsymbol{1-}\boldsymbol{1}_{\boldsymbol{test}}\left( t \right)\cdot rt_{ER}\left( t \right) \right)\boldsymbol{\odot}p_{cr}\cdot\left( \boldsymbol{1-}\sigma_{ER}\cdot\boldsymbol{ihr} \right)\boldsymbol{\odot ER}$  $\boldsymbol{+}\gamma\cdot\left( 1-p_{cr} \right)\cdot\left( \boldsymbol{1-}\boldsymbol{1}_{\boldsymbol{test}}\left( t \right)\cdot rt_{ER}\left( t \right) \right)\boldsymbol{\odot}s_{eff}\left( t \right)\cdot\left( \boldsymbol{1-}\sigma_{ER}\cdot\boldsymbol{ihr} \right)\boldsymbol{\odot ER}$  $\boldsymbol{+ Ag}\cdot\boldsymbol{X -}\nu\cdot\boldsymbol{X -\mu\odot X}$. | $\boldsymbol{(} SEQ Ecuación \backslash* ARABIC 7\boldsymbol{)}$ |
| --- | --- | --- | --- |

|  | $\frac{d\boldsymbol{V}}{dt} =$ | $vac\left( t \right)\cdot\boldsymbol{1}_{\boldsymbol{vac}}\left( t \right)\boldsymbol{\odot S+Ag}\cdot\boldsymbol{V+}\omega\cdot\boldsymbol{VR-}\left( \left( 1-vac_{eff} \right)\cdot\boldsymbol{\lambda}\left( t \right) \right)\boldsymbol{\odot V-\mu\odot V}$  $\boldsymbol{-}vac_{d}\cdot\boldsymbol{V -}qr(t)\cdot\boldsymbol{V}$. | $\boldsymbol{(} SEQ Ecuación \backslash* ARABIC 8\boldsymbol{)}$ |
| --- | --- | --- | --- |

|  | $\frac{d\boldsymbol{EV}}{dt} =$ | $\left( 1-vac_{eff} \right)\cdot\boldsymbol{\lambda}(t)\boldsymbol{\odot V+Ag}\cdot\boldsymbol{EV+}\left( \frac{1}{qd} \right)\cdot\boldsymbol{QEV-}\gamma\cdot\boldsymbol{EV-\mu\odot EV}$  $\boldsymbol{-}qr\left( t \right)\cdot\boldsymbol{EV}$. | $\boldsymbol{(} SEQ Ecuación \backslash* ARABIC 9\boldsymbol{)}$ |
| --- | --- | --- | --- |

|  | $\frac{d\boldsymbol{ER}}{dt}=$ | $\boldsymbol{\lambda}\left( t \right)\cdot\sigma_{Rmod}\left( t \right)\boldsymbol{\odot R+Ag}\cdot\boldsymbol{ER+}\left( \frac{1}{qd} \right)\cdot\boldsymbol{QER-}\gamma\cdot\boldsymbol{ER-\mu\odot ER-}qr\left( t \right)\cdot\boldsymbol{ER}$. | $\boldsymbol{(} SEQ Ecuación \backslash* ARABIC 10\boldsymbol{)}$ |
| --- | --- | --- | --- |

|  | $\frac{d\boldsymbol{VR}}{dt}\boldsymbol{=}$ | $vac\left( t \right)\cdot\boldsymbol{1}_{\boldsymbol{vac}}\left( t \right)\boldsymbol{\odot E+}vac\left( t \right)\cdot\boldsymbol{1}_{\boldsymbol{vac}}\left( t \right)\boldsymbol{\odot I+}vac\left( t \right)\cdot\boldsymbol{1}_{\boldsymbol{vac}}\left( t \right)\boldsymbol{\odot R+Ag}\cdot\boldsymbol{VR}$  $+\left( \frac{1}{qd} \right)\cdot\boldsymbol{QVR-}\left( 1-vac_{effr} \right)\cdot\boldsymbol{\lambda}\left( t \right)\boldsymbol{\odot VR-}vac_{dr}\cdot\boldsymbol{VR-\mu\odot VR}$  $\boldsymbol{-}\omega\cdot\boldsymbol{VR-}qr(t)\cdot\boldsymbol{VR}$. | $\boldsymbol{(} SEQ Ecuación \backslash* ARABIC 11\boldsymbol{)}$ |
| --- | --- | --- | --- |

|  | $\frac{d\boldsymbol{EVR}}{dt} =$ | $\left( 1-vac_{effr} \right)\cdot\boldsymbol{\lambda}\left( t \right)\boldsymbol{\odot VR+}\left( \frac{1}{qd} \right)\cdot\boldsymbol{QEVR-}\gamma\cdot\boldsymbol{EVR+Ag}\cdot\boldsymbol{EVR-\mu\odot EVR}$  $\boldsymbol{-}qr\left( t \right)\cdot\boldsymbol{EVR}$. | $\boldsymbol{(} SEQ Ecuación \backslash* ARABIC 12\boldsymbol{)}$ |
| --- | --- | --- | --- |

|  | $\frac{d\boldsymbol{QS}}{dt}=$ | $qr(t)\cdot\boldsymbol{S+Ag}\cdot\boldsymbol{QS-\mu\odot QS-}\left( \frac{1}{qd} \right)\cdot\boldsymbol{QS-\varrho}(t)\boldsymbol{\odot QS}$. | $\boldsymbol{(} SEQ Ecuación \backslash* ARABIC 13\boldsymbol{)}$ |
| --- | --- | --- | --- |

|  | $\frac{d\boldsymbol{QE}}{dt}=$ | $qr\left( t \right)\cdot\boldsymbol{E+Ag}\cdot\boldsymbol{QE+\varrho}\left( t \right)\boldsymbol{\odot QS+ \varrho}(t)\boldsymbol{\odot QSR-}\gamma\cdot\boldsymbol{QE-\mu\odot QE}$  $\boldsymbol{-}\left( \frac{1}{qd} \right)\cdot\boldsymbol{QE}$. | $\boldsymbol{(} SEQ Ecuación \backslash* ARABIC 14\boldsymbol{)}$ |
| --- | --- | --- | --- |

|  | $\frac{d\boldsymbol{QSR}}{dt}=$ | $qr(t)\cdot\boldsymbol{SR+Ag}\cdot\boldsymbol{QSR-\mu\odot QSR-}\left( \frac{1}{qd} \right) \cdot\boldsymbol{QSR-\varrho}(t)\boldsymbol{\odot QSR}$. | $\boldsymbol{(} SEQ Ecuación \backslash* ARABIC 15\boldsymbol{)}$ |
| --- | --- | --- | --- |

|  | $\frac{d\boldsymbol{QI}}{dt} =$ | $qr\left( t \right)\cdot\boldsymbol{I+}\left( \gamma\cdot\left( \boldsymbol{1-ihr} \right)\cdot\left( 1-p_{c} \right) \right)\boldsymbol{\odot QE+ Ag}\cdot\boldsymbol{QI-}\nu\cdot\boldsymbol{QI}$  $\boldsymbol{-\mu\odot QI-}\left( \frac{1}{qd} \right)\cdot\boldsymbol{QI}$  $\boldsymbol{+}\gamma\cdot\left( \boldsymbol{1-}\sigma_{EV}\cdot\boldsymbol{ihr} \right)\boldsymbol{\odot}\left( 1-p_{cv} \right)\cdot\boldsymbol{QEV}$  $\boldsymbol{+}\gamma\cdot\left( \boldsymbol{1-}\sigma_{ER}\cdot\boldsymbol{ihr} \right)\boldsymbol{\odot}\left( 1-p_{r} \right)\cdot\boldsymbol{QER}$  $\boldsymbol{+}\gamma\cdot\left( \boldsymbol{1-}\sigma_{EVR}\cdot\boldsymbol{ihr} \right)\boldsymbol{\odot}\left( 1-p_{cvr} \right)\cdot\boldsymbol{QEVR}$. | $\boldsymbol{(} SEQ Ecuación \backslash* ARABIC 16\boldsymbol{)}$ |
| --- | --- | --- | --- |

|  | $\frac{d\boldsymbol{QC}}{dt} =$ | $\left( \gamma\cdot p_{c}\cdot\left( 1-self\left( t \right) \right)\cdot\left( \boldsymbol{1-}\boldsymbol{1}_{\boldsymbol{test}}\left( t \right)\cdot rt_{E}\left( t \right) \right)\boldsymbol{\odot}\left( \boldsymbol{1-ihr} \right) \right)\boldsymbol{\odot}qr\left( t \right)\cdot\boldsymbol{E}$  $\boldsymbol{+}\gamma\cdot p_{cv}\cdot\left( \boldsymbol{1-}\boldsymbol{1}_{\boldsymbol{test}}\left( t \right)\cdot rt_{EV}\left( t \right) \right)\boldsymbol{\odot}\left( 1-self\left( t \right) \right)\cdot\left( \boldsymbol{1-}\sigma_{EV}\cdot\boldsymbol{ihr} \right)\boldsymbol{\odot}qr\left( t \right)\cdot\boldsymbol{EV}$  $\boldsymbol{+}\gamma\cdot p_{cr}\cdot\left( \boldsymbol{1-}\boldsymbol{1}_{\boldsymbol{test}}\left( t \right)\cdot rt_{ER}\left( t \right) \right)\boldsymbol{\odot}\left( 1-self\left( t \right) \right)\cdot\left( \boldsymbol{1-}\sigma_{ER}\cdot\boldsymbol{ihr} \right)\boldsymbol{\odot}qr\left( t \right)\cdot\boldsymbol{ER}$  $\boldsymbol{+}\gamma\cdot p_{cvr}\cdot\left( \boldsymbol{1-}\boldsymbol{1}_{\boldsymbol{test}}\left( t \right)\cdot rt_{EVR}\left( t \right) \right)\boldsymbol{\odot}\left( 1-self\left( t \right) \right)\cdot\left( \boldsymbol{1-}\sigma_{EVR}\cdot\boldsymbol{ihr} \right)\boldsymbol{\odot}qr\left( t \right)\cdot\boldsymbol{EVR}$  $\boldsymbol{+}\gamma\cdot\left( \boldsymbol{1-ihr} \right)\boldsymbol{\odot}p_{c}\cdot\boldsymbol{QE +}\gamma\cdot\left( \boldsymbol{1-}\sigma_{EV}\cdot\boldsymbol{ihr} \right)\boldsymbol{\odot}p_{cv}\cdot\boldsymbol{QEV}$  $\boldsymbol{+}\gamma\cdot\left( \boldsymbol{1-}\sigma_{ER}\cdot\boldsymbol{ihr} \right)\boldsymbol{\odot}p_{cr}\cdot\boldsymbol{QER+}\gamma\cdot\left( \boldsymbol{1-}\sigma_{EVR}\cdot\boldsymbol{ihr} \right)\boldsymbol{\odot}p_{cvr}\cdot\boldsymbol{QEVR}$  $\boldsymbol{-}\nu\cdot\boldsymbol{QC+ Ag}\cdot\boldsymbol{QC-\mu}\boldsymbol{\odot}\boldsymbol{QC-}\left( \frac{1}{qd} \right)\cdot\boldsymbol{QC}$. | $\boldsymbol{(} SEQ Ecuación \backslash* ARABIC 17\boldsymbol{)}$ |
| --- | --- | --- | --- |

|  | $\frac{d\boldsymbol{QR}}{dt} =$ | $\nu\cdot\boldsymbol{QI+}\nu\cdot\boldsymbol{QC+Ag}\cdot\boldsymbol{QR-\mu\odot QR+}qr\left( t \right)\cdot\boldsymbol{R+}vac_{dr}\cdot\boldsymbol{QVR}$  $\boldsymbol{-}\sigma_{Rmod}(t)\cdot\boldsymbol{\varrho}(t)\boldsymbol{\odot QR-}\left( \frac{1}{qd} \right)\cdot\boldsymbol{QR}$. | $\boldsymbol{(} SEQ Ecuación \backslash* ARABIC 18\boldsymbol{)}$ |
| --- | --- | --- | --- |

|  | $\frac{d\boldsymbol{QV}}{dt} =$ | $qr\left( t \right)\cdot\boldsymbol{V+Ag}\cdot\boldsymbol{QV+}\omega\cdot\boldsymbol{QVR-\mu\odot QV-}\left( \frac{1}{qd} \right)\cdot\boldsymbol{QV}$  $\boldsymbol{-}\left( 1-vac_{eff} \right)\cdot\boldsymbol{\varrho}\left( t \right)\boldsymbol{\odot QV}$. | $\boldsymbol{(} SEQ Ecuación \backslash* ARABIC 19\boldsymbol{)}$ |
| --- | --- | --- | --- |

|  | $\frac{d\boldsymbol{QEV}}{dt} =$ | $qr\left( t \right)\cdot\boldsymbol{EV+Ag}\cdot\boldsymbol{QEV+}\left( 1-vac_{eff} \right)\cdot\boldsymbol{\varrho}(t)\boldsymbol{\odot QV-}\gamma\cdot\boldsymbol{QEV-\mu\odot QEV}$  $\boldsymbol{-}\left( \frac{1}{qd} \right)\cdot\boldsymbol{QEV}$. | $\boldsymbol{(} SEQ Ecuación \backslash* ARABIC 20\boldsymbol{)}$ |
| --- | --- | --- | --- |

|  | $\frac{d\boldsymbol{QER}}{dt} =$ | $qr\left( t \right)\cdot\boldsymbol{ER+}\sigma_{Rmod}(t)\cdot\boldsymbol{\varrho}(t)\boldsymbol{\odot QR+Ag}\cdot\boldsymbol{QER-}\gamma\cdot\boldsymbol{QER-\mu\odot QER}$  $\boldsymbol{-}\left( \frac{1}{qd} \right)\cdot\boldsymbol{QER}$. | $\boldsymbol{(} SEQ Ecuación \backslash* ARABIC 21\boldsymbol{)}$ |
| --- | --- | --- | --- |

|  | $\frac{d\boldsymbol{QVR}}{dt} =$ | $qr\left( t \right)\cdot\boldsymbol{VR+ Ag}\cdot\boldsymbol{QVR-}\left( 1-vac_{effr} \right)\cdot\boldsymbol{\lambda}\left( t \right)\boldsymbol{\odot QVR-}vac_{dr}\cdot\boldsymbol{QVR}$  $\boldsymbol{-}\omega\cdot\boldsymbol{QVR- \mu\odot QVR}$. | $\boldsymbol{(} SEQ Ecuación \backslash* ARABIC 22\boldsymbol{)}$ |
| --- | --- | --- | --- |

|  | $\frac{d\boldsymbol{QEVR}}{dt} =$ | $qr\left( t \right)\cdot\boldsymbol{EVR+Ag}\cdot\boldsymbol{QEVR+}\left( 1-vac_{effr} \right)\cdot\boldsymbol{\varrho}(t)\boldsymbol{\odot QVR-}\gamma\cdot\boldsymbol{QEVR}$  $\boldsymbol{-\mu\odot QEVR-}\left( \frac{1}{qd} \right)\cdot\boldsymbol{QEVR}$. | $\boldsymbol{(} SEQ Ecuación \backslash* ARABIC 23\boldsymbol{)}$ |
| --- | --- | --- | --- |

|  | $\frac{d\boldsymbol{H}}{dt} =$ | $\gamma\cdot\boldsymbol{ihr\odot}\left( 1-p_{icu} \right)\cdot\left( 1-c_{H} \right)\cdot r_{h}\cdot\boldsymbol{E}$  $\boldsymbol{+}\gamma\cdot\sigma_{ER}\cdot\boldsymbol{ihr\odot}\left( 1-p_{icur} \right)\cdot\left( 1-c_{H} \right)\cdot r_{h}\cdot\boldsymbol{ER}$  $\boldsymbol{+}\gamma\cdot\sigma_{EV}\cdot\boldsymbol{ihr\odot}\left( 1-p_{icuv} \right)\cdot\left( 1-c_{H} \right)\cdot r_{h}\cdot\boldsymbol{EV}$  $\boldsymbol{+}\gamma\cdot\sigma_{EVR}\cdot\boldsymbol{ihr\odot}\left( 1-p_{icuvr} \right)\cdot\left( 1-c_{H} \right)\cdot r_{h}\cdot\boldsymbol{EVR}$  $\boldsymbol{+}\gamma\cdot\boldsymbol{ihr\odot}\left( 1-p_{icu} \right)\cdot\left( 1-c_{H} \right)\cdot r_{h}\cdot\boldsymbol{QE}$  $\boldsymbol{+}\gamma\cdot\sigma_{ER}\cdot\boldsymbol{ihr\odot}\left( 1-p_{icur} \right)\cdot\left( 1-c_{H} \right)\cdot r_{h}\cdot\boldsymbol{QER}$  $\boldsymbol{+}\gamma\cdot\sigma_{EV}\cdot\boldsymbol{ihr\odot}\left( 1-p_{icuv} \right)\cdot\left( 1-c_{H} \right)\cdot r_{h}\cdot\boldsymbol{QEV}$  $\boldsymbol{+}\gamma\cdot\sigma_{EVR}\cdot\boldsymbol{ihr\odot}\left( 1-p_{icuvr} \right)\cdot\left( 1-c_{H} \right)\cdot r_{h}\cdot\boldsymbol{QEVR}$  $\boldsymbol{+ Ag}\cdot\boldsymbol{H-}\nu_{s}\cdot\boldsymbol{H-\mu\odot H.}$ | $\boldsymbol{(} SEQ Ecuación \backslash* ARABIC 24\boldsymbol{)}$ |
| --- | --- | --- | --- |

|  | $\frac{d\boldsymbol{HC}}{dt} =$ | $\gamma\cdot\boldsymbol{ihr}\boldsymbol{\odot}\left( 1-p_{icu} \right)\cdot\left( 1-r_{h} \right)\cdot\boldsymbol{E}$ $\boldsymbol{+}\gamma\cdot\boldsymbol{ihr\odot}\left( 1-p_{icu} \right)\cdot c_{H}\cdot r_{h}\cdot\boldsymbol{E}$  $\boldsymbol{+}\gamma\cdot\boldsymbol{ihr\odot}\left( 1-p_{icu} \right)\cdot\left( 1-r_{h} \right)\cdot\boldsymbol{QE}$ $\boldsymbol{+}\gamma\cdot\boldsymbol{ihr\odot}\left( 1-p_{icu} \right)\cdot c_{H}\cdot r_{h}\cdot\boldsymbol{QE}$  $\boldsymbol{+}\gamma\cdot\sigma_{EV}\cdot\boldsymbol{ihr\odot}\left( 1-p_{icuv} \right)\cdot\left( 1-r_{h} \right)\cdot\boldsymbol{EV}$  $\boldsymbol{+}\gamma\cdot\sigma_{EV}\cdot\boldsymbol{ihr\odot}\left( 1-p_{icuv} \right)\cdot c_{H}\cdot r_{h}\cdot\boldsymbol{EV}$  $\boldsymbol{+}\gamma\cdot\sigma_{EVR}\cdot\boldsymbol{ihr\odot}\left( 1-p_{icuvr} \right)\cdot\left( 1-r_{h} \right)\cdot\boldsymbol{EVR}$  $\boldsymbol{+}\gamma\cdot\sigma_{EVR}\cdot\boldsymbol{ihr\odot}\left( 1-p_{icuvr} \right)\cdot c_{H}\cdot r_{h}\cdot\boldsymbol{EVR}$  $\boldsymbol{+}\gamma\cdot\sigma_{ER}\cdot\boldsymbol{ihr\odot}\left( 1-p_{icur} \right)\cdot\left( 1-r_{h} \right)\cdot\boldsymbol{ER}$  $\boldsymbol{+}\gamma\cdot\sigma_{ER}\cdot\boldsymbol{ihr\odot}\left( 1-p_{icur} \right)\cdot c_{H}\cdot r_{h}\cdot\boldsymbol{ER}$  $\boldsymbol{+}\gamma\cdot\sigma_{EV}\cdot\boldsymbol{ihr\odot}\left( 1-p_{icuv} \right)\cdot\left( 1-r_{h} \right)\cdot\boldsymbol{QEV}$  $\boldsymbol{+}\gamma\cdot\sigma_{EV}\cdot\boldsymbol{ihr\odot}\left( 1-p_{icuv} \right)\cdot c_{H}\cdot r_{h}\cdot\boldsymbol{QEV}$  $\boldsymbol{+}\gamma\cdot\sigma_{EVR}\cdot\boldsymbol{ihr\odot}\left( 1-p_{icuvr} \right)\cdot\left( 1-r_{h} \right)\cdot\boldsymbol{QEVR}$  $\boldsymbol{+}\gamma\cdot\sigma_{EVR}\cdot\boldsymbol{ihr\odot}\left( 1-p_{icuvr} \right)\cdot c_{H}\cdot r_{h}\cdot\boldsymbol{QEVR}$  $\boldsymbol{+}\gamma\cdot\sigma_{ER}\cdot\boldsymbol{ihr\odot}\left( 1-p_{icur} \right)\cdot\left( 1-r_{h} \right)\cdot\boldsymbol{QER}$  $\boldsymbol{+}\gamma\cdot\sigma_{ER}\cdot\boldsymbol{ihr\odot}\left( 1-p_{icur} \right)\cdot c_{H}\cdot r_{h}\cdot\boldsymbol{QER}$ $\boldsymbol{+ Ag}\cdot\boldsymbol{HC}\boldsymbol{-}\nu_{sc}\cdot\boldsymbol{HC-\mu\odot HC-}rt_{HC}(t)\cdot\boldsymbol{1}_{\boldsymbol{test}}(t)\boldsymbol{\odot HC}$. | $\boldsymbol{(} SEQ Ecuación \backslash* ARABIC 25\boldsymbol{)}$ |
| --- | --- | --- | --- |

|  | $\frac{d\boldsymbol{HCICU}}{dt} =$ | $\gamma\cdot\left( 1-r_{hICU} \right)\cdot\boldsymbol{ihr\odot}p_{icu}\cdot\left( 1-p_{v} \right)\cdot\boldsymbol{E}$  $\boldsymbol{+}\gamma\cdot\left( 1-r_{hICU} \right)\cdot\sigma_{ER}\cdot\boldsymbol{ihr\odot}p_{icur}\cdot\left( 1-p_{vr} \right)\cdot\boldsymbol{ER}$  $\boldsymbol{+}\gamma\cdot\left( 1-r_{hICU} \right)\cdot\sigma_{EV}\cdot\boldsymbol{ihr\odot}p_{icuv}\cdot\left( 1-p_{vv} \right)\cdot\boldsymbol{EV}$  $\boldsymbol{+}\gamma\cdot\left( 1-r_{hICU} \right)\cdot\sigma_{EVR}\cdot\boldsymbol{ihr\odot}p_{icuvr}\cdot\left( 1-p_{vvr} \right)\cdot\boldsymbol{EVR}$  $\boldsymbol{+}\gamma\cdot\left( 1-r_{hICU} \right)\cdot\boldsymbol{ihr\odot}p_{icu}\cdot\left( 1-p_{v} \right)\cdot\boldsymbol{QE}$  $\boldsymbol{+}\gamma\cdot\left( 1-r_{hICU} \right)\cdot\sigma_{ER}\cdot\boldsymbol{ihr\odot}p_{icur}\cdot\left( 1-p_{vr} \right)\cdot\boldsymbol{QER}$  $\boldsymbol{+}\gamma\cdot\left( 1-r_{hICU} \right)\cdot\sigma_{EV}\cdot\boldsymbol{ihr\odot}p_{icuv}\cdot\left( 1-p_{vv} \right)\cdot\boldsymbol{QEV}$  $\boldsymbol{+}\gamma\cdot\left( 1-r_{hICU} \right)\cdot\sigma_{EVR}\cdot\boldsymbol{ihr\odot}p_{icuvr}\cdot\left( 1-p_{vvr} \right)\cdot\boldsymbol{QEVR}$  $\boldsymbol{+ Ag}\cdot\boldsymbol{HCICU-}\nu_{sc}\cdot\boldsymbol{HCICU-\mu\odot HCICU}$  $\boldsymbol{-}rt_{HCICU}(t)\cdot\boldsymbol{1}_{\boldsymbol{test}}(t)\boldsymbol{\odot HCICU.}$ | $\boldsymbol{(} SEQ Ecuación \backslash* ARABIC 26\boldsymbol{)}$ |
| --- | --- | --- | --- |

|  | $\frac{d\boldsymbol{HCV}}{dt} =$ | $\gamma\cdot\left( 1-r_{hICU} \right)\cdot\boldsymbol{ihr\odot}p_{icu}\cdot p_{v}\cdot\boldsymbol{E}$  $\boldsymbol{+}\gamma\cdot\left( 1-r_{hICU} \right)\cdot\sigma_{EV}\cdot\boldsymbol{ihr\odot}p_{icuv}\cdot p_{vv}\cdot\boldsymbol{EV}$  $\boldsymbol{+}\gamma\cdot\left( 1-r_{hICU} \right)\cdot\sigma_{EVR}\cdot\boldsymbol{ihr\odot}p_{icuvr}\cdot p_{vvr}\cdot\boldsymbol{EVR}$  $\boldsymbol{+}\gamma\cdot\left( 1-r_{hICU} \right)\cdot\sigma_{ER}\cdot\boldsymbol{ihr\odot}p_{icur}\cdot p_{vr}\cdot\boldsymbol{ER}$  $\boldsymbol{+}\gamma\cdot\left( 1-r_{hICU} \right)\cdot\boldsymbol{ihr\odot}p_{icu}\cdot p_{v}\cdot\boldsymbol{QE}$  $\boldsymbol{+}\gamma\cdot\left( 1-r_{hICU} \right)\cdot\sigma_{EV}\cdot\boldsymbol{ihr\odot}p_{icuv}\cdot p_{vv}\cdot\boldsymbol{QEV}$  $\boldsymbol{+}\gamma\cdot\left( 1-r_{hICU} \right)\cdot\sigma_{EVR}\cdot\boldsymbol{ihr\odot}p_{icuvr}\cdot p_{vvr}\cdot\boldsymbol{QEVR}$  $\boldsymbol{+}\gamma\cdot\left( 1-r_{hICU} \right)\cdot\sigma_{ER}\cdot\boldsymbol{ihr\odot}p_{icur}\cdot p_{vr}\cdot\boldsymbol{QER}$  $\boldsymbol{+ Ag}\cdot\boldsymbol{HCV-}\nu_{vc}\cdot\boldsymbol{HCV-\mu\odot HCV-}rt_{HCV}(t)\cdot\boldsymbol{1}_{\boldsymbol{test}}(t)\boldsymbol{\odot HCV}$. | $\boldsymbol{(} SEQ Ecuación \backslash* ARABIC 27\boldsymbol{)}$ |
| --- | --- | --- | --- |

|  | $\frac{d\boldsymbol{ICU}}{dt} =$ | $\gamma\cdot r_{hICU}\cdot\boldsymbol{ihr\odot}p_{icu}\cdot\left( 1-c \right)\cdot\left( 1-p_{v} \right)\cdot\boldsymbol{E}$  $\boldsymbol{+}\gamma\cdot r_{hICU}\cdot\boldsymbol{ihr\odot}p_{icu}\cdot\left( 1-c \right)\cdot\left( 1-p_{v} \right)\cdot\boldsymbol{QE}$  $\boldsymbol{+}\gamma\cdot r_{hICU}\cdot\sigma_{EV}\cdot\boldsymbol{ihr\odot}p_{icuv}\cdot\left( 1-c \right)\cdot\left( 1-p_{vv} \right)\cdot\boldsymbol{EV}$  $\boldsymbol{+}\gamma\cdot r_{hICU}\cdot\sigma_{EVR}\cdot\boldsymbol{ihr\odot}p_{icuvr}\cdot\left( 1-c \right)\cdot\left( 1-p_{vvr} \right)\cdot\boldsymbol{EVR}$  $\boldsymbol{+}\gamma\cdot r_{hICU}\cdot\sigma_{ER}\cdot\boldsymbol{ihr\odot}p_{icur}\cdot\left( 1-c \right)\cdot\left( 1-p_{vr} \right)\cdot\boldsymbol{ER}$  $\boldsymbol{+}\gamma\cdot r_{hICU}\cdot\sigma_{EV}\cdot\boldsymbol{ihr\odot}p_{icuv}\cdot\left( 1-c \right)\cdot\left( 1-p_{vv} \right)\cdot\boldsymbol{QEV}$  $\boldsymbol{+}\gamma\cdot r_{hICU}\cdot\sigma_{EVR}\cdot\boldsymbol{ihr\odot}p_{icuvr}\cdot\left( 1-c \right)\cdot\left( 1-p_{vvr} \right)\cdot\boldsymbol{QEVR}$  $\boldsymbol{+}\gamma\cdot r_{hICU}\cdot\sigma_{ER}\cdot\boldsymbol{ihr\odot}p_{icur}\cdot\left( 1-c \right)\cdot\left( 1-p_{vr} \right)\cdot\boldsymbol{QER}$  $\boldsymbol{+ Ag}\cdot\boldsymbol{ICU+}\left( 1-c \right)\cdot\boldsymbol{ICUC}\cdot1/2\boldsymbol{-}\nu_{icu}\cdot\boldsymbol{ICU-\mu\odot ICU}$. | $\boldsymbol{(} SEQ Ecuación \backslash* ARABIC 28\boldsymbol{)}$ |
| --- | --- | --- | --- |

|  | $\frac{d\boldsymbol{ICUC}}{dt} =$ | $\gamma\cdot r_{hICU}\cdot\boldsymbol{ihr\odot}p_{icu}\cdot c\cdot\left( 1-p_{v} \right)\cdot\boldsymbol{E}$  $\boldsymbol{+}\gamma\cdot r_{hICU}\cdot\boldsymbol{ihr\odot}p_{icu}\cdot c\cdot\left( 1-p_{v} \right)\cdot\boldsymbol{QE}$  $\boldsymbol{+}\gamma\cdot r_{hICU}\cdot\sigma_{EV}\cdot\boldsymbol{ihr\odot}p_{icuv}\cdot c\cdot\left( 1-p_{vv} \right)\cdot\boldsymbol{EV}$  $\boldsymbol{+}\gamma\cdot r_{hICU}\cdot\sigma_{EVR}\cdot\boldsymbol{ihr\odot}p_{icuvr}\cdot c\cdot\left( 1-p_{vvr} \right)\cdot\boldsymbol{EVR}$  $\boldsymbol{+}\gamma\cdot r_{hICU}\cdot\sigma_{ER}\cdot\boldsymbol{ihr\odot}p_{icur}\cdot c\cdot\left( 1-p_{vr} \right)\cdot\boldsymbol{ER}$  $\boldsymbol{+}\gamma\cdot r_{hICU}\cdot\sigma_{EV}\cdot\boldsymbol{ihr\odot}p_{icuv}\cdot c\cdot\left( 1-p_{vv} \right)\cdot\boldsymbol{QEV}$  $\boldsymbol{+}\gamma\cdot r_{hICU}\cdot\sigma_{EVR}\cdot\boldsymbol{ihr\odot}p_{icuvr}\cdot c\cdot\left( 1-p_{vvr} \right)\cdot\boldsymbol{QEVR}$  $\boldsymbol{+}\gamma\cdot r_{hICU}\cdot\sigma_{ER}\cdot\boldsymbol{ihr\odot}p_{icur}\cdot c\cdot\left( 1-p_{vr} \right)\cdot\boldsymbol{QER}$  $\boldsymbol{+ Ag}\cdot\boldsymbol{ICUC-}\nu_{icuc}\cdot\boldsymbol{ICUC}-\left( 1-c \right)\cdot\boldsymbol{ICUC}\cdot1/2\boldsymbol{-\mu\odot ICUC}$. | $\boldsymbol{(} SEQ Ecuación \backslash* ARABIC 29\boldsymbol{)}$ |
| --- | --- | --- | --- |

|  | $\frac{d\boldsymbol{ICUCV}}{dt} =$ | $\gamma\cdot r_{hICU}\cdot\boldsymbol{ihr\odot}p_{icu}\cdot p_{v}\cdot c\cdot\boldsymbol{E}$  $\boldsymbol{+}\gamma\cdot r_{hICU}\cdot\boldsymbol{ihr\odot}p_{icu}\cdot p_{v}\cdot c\cdot\boldsymbol{QE}$  $\boldsymbol{+}\gamma\cdot r_{hICU}\cdot\sigma_{EV}\cdot\boldsymbol{ihr\odot}p_{icuv}\cdot p_{vv}\cdot c\cdot\boldsymbol{EV}$  $\boldsymbol{+}\gamma\cdot r_{hICU}\cdot\sigma_{EVR}\cdot\boldsymbol{ihr\odot}p_{icuvr}\cdot p_{vvr}\cdot c\cdot\boldsymbol{EVR}$  $\boldsymbol{+}\gamma\cdot r_{hICU}\cdot\sigma_{ER}\cdot\boldsymbol{ihr\odot}p_{icur}\cdot p_{vr}\cdot c\cdot\boldsymbol{ER}$  $\boldsymbol{+}\gamma\cdot r_{hICU}\cdot\sigma_{EV}\cdot\boldsymbol{ihr\odot}p_{icuv}\cdot p_{vv}\cdot c\cdot\boldsymbol{QEV}$  $\boldsymbol{+}\gamma\cdot r_{hICU}\cdot\sigma_{EVR}\cdot\boldsymbol{ihr\odot}p_{icuvr}\cdot p_{vvr}\cdot c\cdot\boldsymbol{QEVR}$  $\boldsymbol{+}\gamma\cdot r_{hICU}\cdot\sigma_{ER}\cdot\boldsymbol{ihr\odot}p_{icur}\cdot p_{vr}\cdot c\cdot\boldsymbol{QER}$  $\boldsymbol{+ Ag}\cdot\boldsymbol{ICUCV}- \nu_{vc}\cdot\boldsymbol{ICUCV-\mu\odot ICUCV}-\left( 1-c_{V} \right)\cdot\boldsymbol{ICUCV}\cdot1/2$ | $\boldsymbol{(} SEQ Ecuación \backslash* ARABIC 30\boldsymbol{)}$ |
| --- | --- | --- | --- |

|  | $\frac{d\boldsymbol{Vent}}{dt} =$ | $\gamma\cdot r_{hICU}\cdot\boldsymbol{ihr\odot}p_{icu}\cdot\left( 1-c \right)\cdot\left( 1-c_{V} \right)\cdot p_{v}\cdot\boldsymbol{E}$  $\boldsymbol{+}\gamma\cdot r_{hICU}\cdot\sigma_{EV}\cdot\boldsymbol{ihr\odot}p_{icuv}\cdot\left( 1-c \right)\cdot\left( 1-c_{V} \right)\cdot p_{vv}\cdot\boldsymbol{EV}$  $\boldsymbol{+}\gamma\cdot r_{hICU}\cdot\sigma_{ER}\cdot\boldsymbol{ihr\odot}p_{icur}\cdot\left( 1-c \right)\cdot\left( 1-c_{V} \right)\cdot p_{vr}\cdot\boldsymbol{ER}$  $\boldsymbol{+}\gamma\cdot r_{hICU}\cdot\sigma_{EVR}\cdot\boldsymbol{ihr\odot}p_{icuvr}\cdot\left( 1-c \right)\cdot\left( 1-c_{V} \right)\cdot p_{vvr}\cdot\boldsymbol{EVR}$  $\boldsymbol{+}\gamma\cdot r_{hICU}\cdot\boldsymbol{ihr\odot}p_{icu}\cdot\left( 1-c \right)\cdot\left( 1-c_{V} \right)\cdot p_{v}\cdot\boldsymbol{QE}$  $\boldsymbol{+}\gamma\cdot r_{hICU}\cdot\sigma_{EV}\cdot\boldsymbol{ihr\odot}p_{icuv}\cdot\left( 1-c \right)\cdot\left( 1-c_{V} \right)\cdot p_{vv}\cdot\boldsymbol{QEV}$  $\boldsymbol{+}\gamma\cdot r_{hICU}\cdot\sigma_{ER}\cdot\boldsymbol{ihr\odot}p_{icur}\cdot\left( 1-c \right)\cdot\left( 1-c_{V} \right)\cdot p_{vr}\cdot\boldsymbol{QER}$  $\boldsymbol{+}\gamma\cdot r_{hICU}\cdot\sigma_{EVR}\cdot\boldsymbol{ihr\odot}p_{icuvr}\cdot\left( 1-c \right)\cdot\left( 1-c_{V} \right)\cdot p_{vvr}\cdot\boldsymbol{QEVR}$  $\boldsymbol{+}\left( 1-c_{V} \right)\cdot\boldsymbol{VentC}\cdot1/2+\left( 1-c_{V} \right)\cdot\boldsymbol{ICUCV}\cdot1/2\boldsymbol{+ Ag}\cdot\boldsymbol{Vent}$  $\boldsymbol{-}\nu_{v}\cdot\boldsymbol{Vent-\mu\odot Vent.}$ | $\boldsymbol{(} SEQ Ecuación \backslash* ARABIC 31\boldsymbol{)}$ |
| --- | --- | --- | --- |

|  | $\frac{d\boldsymbol{VentC}}{dt} =$ | $\gamma\cdot r_{hICU}\cdot\boldsymbol{ihr\odot}p_{icu}\cdot p_{v}\cdot\left( 1-c \right)\cdot c_{V}\cdot\boldsymbol{E}$  $\boldsymbol{+}\gamma\cdot r_{hICU}\cdot\sigma_{EV}\cdot\boldsymbol{ihr\odot}p_{icuv}\cdot p_{vv}\cdot\left( 1-c \right)\cdot c_{V}\cdot\boldsymbol{EV}$  $\boldsymbol{+}\gamma\cdot r_{hICU}\cdot\sigma_{EVR}\cdot\boldsymbol{ihr\odot}p_{icuvr}\cdot p_{vvr}\cdot\left( 1-c \right)\cdot c_{V}\cdot\boldsymbol{EVR}$  $\boldsymbol{+}\gamma\cdot r_{hICU}\cdot\sigma_{ER}\cdot\boldsymbol{ihr\odot}p_{icur}\cdot p_{vr}\cdot\left( 1-c \right)\cdot c_{V}\cdot\boldsymbol{ER}$  $\boldsymbol{+}\gamma\cdot r_{hICU}\cdot\boldsymbol{ihr\odot}p_{icu}\cdot p_{v}\cdot\left( 1-crit \right)\cdot c_{V}\cdot\boldsymbol{QE}$  $\boldsymbol{+}\gamma\cdot r_{hICU}\cdot\sigma_{EV}\cdot\boldsymbol{ihr\odot}p_{icuv}\cdot p_{vv}\cdot\left( 1-c \right)\cdot c_{V}\cdot\boldsymbol{QEV}$  $\boldsymbol{+}\gamma\cdot r_{hICU}\cdot\sigma_{EVR}\cdot\boldsymbol{ihr\odot}p_{icuvr}\cdot p_{vvr}\cdot\left( 1-c \right)\cdot c_{V}\cdot\boldsymbol{QEVR}$  $\boldsymbol{+}\gamma\cdot r_{hICU}\cdot\sigma_{ER}\cdot\boldsymbol{ihr\odot}p_{icur}\cdot p_{vr}\cdot\left( 1-c \right)\cdot c_{V}\cdot\boldsymbol{QER}$  $\boldsymbol{+ Ag}\cdot\boldsymbol{VentC-}\left( 1-c_{V} \right)\cdot\boldsymbol{VentC}\cdot1/2- \nu_{vc}\cdot\boldsymbol{VentC-\mu\odot VentC.}$ | $\boldsymbol{(} SEQ Ecuación \backslash* ARABIC 32\boldsymbol{)}$ |
| --- | --- | --- | --- |

|  | $\frac{d\boldsymbol{C}}{dt} =$ | $r\cdot\gamma\cdot\left( \boldsymbol{1-}\boldsymbol{1}_{\boldsymbol{test}}\left( t \right)\cdot rt_{E}\left( t \right) \right)\boldsymbol{\odot}\left( 1-p_{c} \right)\cdot\left( \boldsymbol{1}-\boldsymbol{ihr} \right)\boldsymbol{\odot}\left( \boldsymbol{E+QE} \right)$  $\boldsymbol{+}r_{c}\cdot\gamma\cdot p_{c}\cdot\left( \boldsymbol{1-}\boldsymbol{1}_{\boldsymbol{test}}\left( t \right)\cdot rt_{E}\left( t \right) \right)\boldsymbol{\odot}\left( \boldsymbol{1}-\boldsymbol{ihr} \right)\boldsymbol{\odot}\left( \boldsymbol{E+QE} \right)$  $\boldsymbol{+}\gamma\cdot\boldsymbol{ihr}\boldsymbol{\odot}\left( 1-c_{H} \right)\cdot\left( 1-p_{icu} \right)\cdot\left( \boldsymbol{E+QE} \right)$  $\boldsymbol{+}\gamma\cdot\boldsymbol{ihr}\boldsymbol{\odot}c_{H}\cdot r_{h}\cdot\left( 1-p_{icu} \right)\cdot\left( \boldsymbol{E+QE} \right)$  $\boldsymbol{+}\gamma\cdot\boldsymbol{ihr}\boldsymbol{\odot}p_{icu}\cdot\left( \boldsymbol{E+QE} \right)\boldsymbol{+}rt_{I}\left( t \right)\cdot\boldsymbol{1}_{\boldsymbol{test}}\left( t \right)\boldsymbol{\odot}\boldsymbol{I}$  $\boldsymbol{+}rt_{C}(t)\cdot\boldsymbol{1}_{\boldsymbol{test}}(t)\boldsymbol{\odot}\boldsymbol{CL+}\gamma\cdot\boldsymbol{1}_{\boldsymbol{test}}(t)\cdot rt_{E}(t)\boldsymbol{\odot}\left( \boldsymbol{1}-\boldsymbol{ihr} \right)\boldsymbol{\odot}\boldsymbol{E}$. | $\boldsymbol{(} SEQ Ecuación \backslash* ARABIC 33\boldsymbol{)}$ |
| --- | --- | --- | --- |

|  | $\frac{d\boldsymbol{CM}}{dt} =$ | $\nu_{s}\cdot p_{ho2}\cdot d_{2}\cdot p_{dho}\cdot dm\left( t \right)\cdot\boldsymbol{ifr\odot H}$  $\boldsymbol{+}\nu_{s}\cdot\left( 1-p_{ho2} \right)\cdot p_{dh}\cdot dm\left( t \right)\cdot\boldsymbol{ifr\odot H}$  $\boldsymbol{+}\nu_{sc}\cdot r_{dHC}\cdot p_{ho2}\cdot p_{dhco}\cdot dm\left( t \right)\cdot\boldsymbol{ifr\odot HC}$  $\boldsymbol{+}\nu_{sc}\cdot r_{dHC}\cdot\left( 1-p_{ho2} \right)\cdot p_{dhc}\cdot dm\left( t \right)\cdot\boldsymbol{ifr\odot HC}$  $\boldsymbol{+}\nu_{icu}\cdot p_{ho2}\cdot d_{2}\cdot p_{dicuo}\cdot dm\left( t \right)\cdot\boldsymbol{ifr\odot ICU}$  $\boldsymbol{+}\nu_{icu}\cdot\left( 1-p_{ho2} \right)\cdot p_{dicu}\cdot dm\left( t \right)\cdot\boldsymbol{ifr\odot ICU}$  $\boldsymbol{+}\nu_{icuc}\cdot p_{ho2}\cdot d_{2c}\cdot p_{dicuco}\cdot dm\left( t \right)\cdot\boldsymbol{ifr\odot ICUC}$  $\boldsymbol{+}\nu_{icuc}\cdot\left( 1-p_{ho2} \right)\cdot p_{dicuc}\cdot dm\left( t \right)\cdot\boldsymbol{ifr\odot ICUC}$  $\boldsymbol{+}\nu_{vc}\cdot d_{vc}\cdot p_{dvc}\cdot dm\left( t \right)\cdot\boldsymbol{ifr\odot ICUCV}$  $\boldsymbol{+}\nu_{v}\cdot d_{v}\cdot p_{dv}\cdot dm\left( t \right)\cdot\boldsymbol{ifr\odot Vent}$  $\boldsymbol{+}\nu_{vc}\cdot d_{vc}\cdot p_{dvc}\cdot dm\left( t \right)\cdot\boldsymbol{ifr\odot VentC}$  $\boldsymbol{+}\nu_{vc}\cdot r_{dHC}\cdot p_{dvhc}\cdot dm\left( t \right)\cdot\boldsymbol{ifr\odot HCV}$  $\boldsymbol{+}\nu_{sc}\cdot r_{dHC}\cdot p_{ho2}\cdot p_{dicuhco}\cdot dm\left( t \right)\cdot\boldsymbol{ifr\odot HCICU}$  $\boldsymbol{+}\nu_{sc}\cdot r_{dHC}\cdot\left( 1-p_{ho2} \right)\cdot p_{dicuhc}\cdot dm\left( t \right)\cdot\boldsymbol{ifr\odot HCICU}$  $\boldsymbol{+ \mu\odot H+\mu\odot ICU+\mu\odot ICUC+\mu\odot ICUCV+\mu\odot Vent+\mu\odot VentC}$  $\boldsymbol{+ \mu\odot Z+}r_{dHC}\cdot\boldsymbol{\mu\odot HC+}r_{dHC}\cdot\boldsymbol{\mu\odot HCICU+}r_{dHC}\cdot\boldsymbol{\mu\odot HCV}$  $\boldsymbol{+}r_{ndI}\cdot\boldsymbol{\mu\odot I+}r_{ndI}\cdot\boldsymbol{\mu\odot QI+}r_{ndI}\cdot\boldsymbol{\mu\odot E+}r_{ndI}\cdot\boldsymbol{\mu\odot QE+}r_{ndI}\cdot\boldsymbol{\mu\odot EV}$  $\boldsymbol{+}r_{ndI}\cdot\boldsymbol{\mu\odot EVR+}r_{ndI}\cdot\boldsymbol{\mu\odot ER}+r_{ndI}\cdot\boldsymbol{\mu\odot QEV+}r_{ndI}\cdot\boldsymbol{\mu\odot QEVR}$  $\boldsymbol{+}r_{ndI}\cdot\boldsymbol{\mu\odot QER+}r_{ndCL}\cdot\boldsymbol{\mu\odot CL+}r_{ndCL}\cdot\boldsymbol{\mu\odot QC+}r_{ndCL}\cdot\boldsymbol{\mu\odot X}$. | $\boldsymbol{(} SEQ Ecuación \backslash* ARABIC 34\boldsymbol{)}$ |
| --- | --- | --- | --- |

|  | $\frac{d\boldsymbol{CMC}}{dt} =$ | $\nu_{sc}\cdot p_{ho2}\cdot p_{dhco}\cdot dm\left( t \right)\cdot\boldsymbol{ifr}\boldsymbol{\odot}\boldsymbol{HC}$  $\boldsymbol{+}\nu_{sc}\cdot\left( 1-p_{ho2} \right)\cdot p_{dhc}\cdot dm\left( t \right)\cdot\boldsymbol{ifr}\boldsymbol{\odot}\boldsymbol{HC}$  $\boldsymbol{+}\nu_{icuc}\cdot p_{ho2}\cdot d_{2c}\cdot p_{dicuco}\cdot dm\left( t \right)\cdot\boldsymbol{ifr}\boldsymbol{\odot}\boldsymbol{ICUC}$  $\boldsymbol{+}\nu_{icuc}\cdot\left( 1-p_{ho2} \right)\cdot p_{dicuc}\cdot dm\left( t \right)\cdot\boldsymbol{ifr}\boldsymbol{\odot}\boldsymbol{ICUC}$  $\boldsymbol{+}\nu_{vc}\cdot d_{vc}\cdot p_{dvc}\cdot dm\left( t \right)\cdot\boldsymbol{ifr}\boldsymbol{\odot}\boldsymbol{ICUCV}$  $\boldsymbol{+}\nu_{vc}\cdot d_{vc}\cdot p_{dvc}\cdot dm\left( t \right)\cdot\boldsymbol{ifr}\boldsymbol{\odot}\boldsymbol{VentC}$  $\boldsymbol{+ \mu}\boldsymbol{\odot}\boldsymbol{HC+\mu}\boldsymbol{\odot}\boldsymbol{ICUC+\mu}\boldsymbol{\odot}\boldsymbol{VentC+\mu}\boldsymbol{\odot}\boldsymbol{ICUCV}$. | $\boldsymbol{(} SEQ Ecuación \backslash* ARABIC 35\boldsymbol{)}$ |
| --- | --- | --- | --- |

|  | $\frac{d\boldsymbol{Z}}{dt} =$ | $\gamma\cdot rt_{E}\left( t \right)\cdot\boldsymbol{1}_{\boldsymbol{test}}\left( t \right)\boldsymbol{\odot}\left( \boldsymbol{1-ihr} \right)\boldsymbol{\odot E+}rt_{I}\left( t \right)\cdot\boldsymbol{1}_{\boldsymbol{test}}\left( t \right)\boldsymbol{\odot I}$  $\boldsymbol{+}\gamma\cdot\left( \boldsymbol{1-ihr} \right)\boldsymbol{\odot}rt_{EV}\left( t \right)\cdot\boldsymbol{1}_{\boldsymbol{test}}\left( t \right)\boldsymbol{\odot EV}$  $\boldsymbol{+}\gamma\cdot\left( \boldsymbol{1-ihr} \right)\boldsymbol{\odot}rt_{ER}\left( t \right)\cdot\boldsymbol{1}_{\boldsymbol{test}}\left( t \right)\boldsymbol{\odot ER}$  $\boldsymbol{+}\gamma\cdot\left( \boldsymbol{1-ihr} \right)\boldsymbol{\odot}rt_{EVR}\left( t \right)\cdot\boldsymbol{1}_{\boldsymbol{test}}\left( t \right)\boldsymbol{\odot EVR}$  $\boldsymbol{+}rt_{C}\left( t \right)\cdot\boldsymbol{1}_{\boldsymbol{test}}\left( t \right)\boldsymbol{\odot CL+}rt_{HC}\left( t \right)\cdot\boldsymbol{1}_{\boldsymbol{test}}\left( t \right)\boldsymbol{\odot HC}$ $\boldsymbol{+}rt_{HCICU}\left( t \right)\cdot\boldsymbol{1}_{\boldsymbol{test}}\left( t \right)\boldsymbol{\odot HCICU+}rt_{HCV}(t)\cdot\boldsymbol{1}_{\boldsymbol{test}}(t)\boldsymbol{\odot HCV}$  $\boldsymbol{-}\left( \frac{1}{id} \right)\cdot\boldsymbol{Z-\mu\odot Z}$. | $\boldsymbol{(} SEQ Ecuación \backslash* ARABIC 36\boldsymbol{)}$ |
| --- | --- | --- | --- |

|  | $\frac{d\boldsymbol{Ab}}{dt} =$ | $\nu\cdot\boldsymbol{I+}\nu\cdot\boldsymbol{X+}\nu\cdot\boldsymbol{CL+}\nu_{s}\cdot p_{ho2}\cdot\left( 1\boldsymbol{-}d_{2}\cdot p_{dho}\cdot dm\left( t \right) \right)\cdot\boldsymbol{ifr\odot H}$  $\boldsymbol{+}\nu_{s}\cdot\left( 1-p_{ho2} \right)\cdot\left( 1\boldsymbol{-}p_{dh}\cdot dm\left( t \right) \right)\cdot\boldsymbol{ifr\odot H}$  $\boldsymbol{+}\nu_{sc}\cdot p_{ho2}\cdot\left( 1\boldsymbol{-}p_{dhco}\cdot dm\left( t \right) \right)\cdot\boldsymbol{ifr\odot HC}$  $\boldsymbol{+}\nu_{sc}\cdot\left( 1-p_{ho2} \right)\cdot\left( 1\boldsymbol{-}p_{dhc}\cdot dm\left( t \right) \right)\cdot\boldsymbol{ifr\odot HC}$  $\boldsymbol{+}\nu_{sc}\cdot p_{ho2}\cdot\left( 1\boldsymbol{-}p_{dicuhco}\cdot dm\left( t \right) \right)\cdot\boldsymbol{ifr\odot HCICU}$  $\boldsymbol{+}\nu_{sc}\cdot\left( 1-p_{ho2} \right)\cdot\left( 1\boldsymbol{-}p_{dicuhc}\cdot dm\left( t \right) \right)\cdot\boldsymbol{ifr\odot HCICU}$  $\boldsymbol{+}\nu_{vc}\cdot\left( 1\boldsymbol{-}p_{dvhc}\cdot dm\left( t \right) \right)\cdot\boldsymbol{ifr\odot HCV}$  $\boldsymbol{+}\nu_{icu}\cdot p_{ho2}\cdot\left( 1\boldsymbol{-}d_{2}\cdot p_{dicuo}\cdot dm\left( t \right) \right)\cdot\boldsymbol{ifr\odot ICU}$  $\boldsymbol{+}\nu_{icu}\cdot\left( 1-p_{ho2} \right)\cdot\left( 1\boldsymbol{-}p_{dicu}\cdot dm\left( t \right) \right)\cdot\boldsymbol{ifr\odot ICU}$  $\boldsymbol{+}\nu_{icuc}\cdot p_{ho2}\cdot\left( 1\boldsymbol{-}d_{2c}\cdot p_{dicuco}\cdot dm\left( t \right) \right)\cdot\boldsymbol{ifr\odot ICUC}$  $\boldsymbol{+}\nu_{icuc}\cdot\left( 1-p_{ho2} \right)\cdot\left( 1\boldsymbol{-}p_{dicuc}\cdot dm\left( t \right) \right)\cdot\boldsymbol{ifr\odot ICUC}$  $\boldsymbol{+}\nu_{vc}\cdot\left( 1\boldsymbol{-}d_{vc}\cdot p_{dvc}\cdot dm(t) \right)\cdot\boldsymbol{ifr\odot ICUCV}$  $\boldsymbol{+}\nu_{v}\cdot\left( 1\boldsymbol{-}d_{v}\cdot p_{dv}\cdot dm\left( t \right) \right)\cdot\boldsymbol{ifr\odot Vent}$  $\boldsymbol{+}\nu_{vc}\cdot\left( 1\boldsymbol{-}d_{vc}\cdot p_{dvc}\cdot dm\left( t \right) \right)\cdot\boldsymbol{ifr\odot VentC}$  $\boldsymbol{+ Ag}\cdot\boldsymbol{Ab-}sg\cdot\boldsymbol{Ab-\mu\odot Ab.}$ | $\boldsymbol{(} SEQ Ecuación \backslash* ARABIC 37\boldsymbol{)}$ |
| --- | --- | --- | --- |

D. Main method (C++ and R) for each non-explicit parameter

Throughout this section, we will treat the compartments as column vectors of size 21, corresponding to age groups. Thus, when a sum operator appears, it represents the sum of all age groups to obtain the total population in each compartment.

Ageing matrix

Set $\alpha=1/(5\cdot365.25)$:

|  | $\boldsymbol{Ag=}$ | $\left( \begin{matrix} \left( \begin{matrix} -\alpha& 0 \\ \alpha& -\alpha\end{matrix} \right) & & \cdots\begin{matrix} & \begin{matrix} & & 0 \end{matrix} \end{matrix} \\ & \ddots& \begin{matrix} & \begin{matrix} & & \end{matrix} \end{matrix} \\ \begin{matrix} \vdots\\ \begin{matrix} \\ \\ 0 \end{matrix} \end{matrix} & \begin{matrix} \\ \begin{matrix} \\ \\ \end{matrix} \end{matrix} & \begin{matrix} \begin{matrix} \left( \begin{matrix} -\alpha& 0 \\ \alpha& -\alpha\end{matrix} \right) & \begin{matrix} \vdots& & \end{matrix} \end{matrix} \\ \begin{matrix} \begin{matrix} \\ \ldots\\ \end{matrix} & \begin{matrix} \begin{matrix} \\ \\ \end{matrix} & \begin{matrix} \ddots\\ \\ \end{matrix} & \begin{matrix} \\ \left( \begin{matrix} -\alpha& 0 \\ \alpha& -\alpha\end{matrix} \right) \\ \end{matrix} \end{matrix} \end{matrix} \end{matrix} \end{matrix} \right).$ | $\boldsymbol{(} SEQ Ecuación \backslash* ARABIC 38\boldsymbol{)}$ |
| --- | --- | --- | --- |

Number of births

|  | $\boldsymbol{b}_{1}\boldsymbol{=}$ | $\sum\left( \boldsymbol{B\odot K} \right)\boldsymbol{.}$ | $\boldsymbol{(} SEQ Ecuación \backslash* ARABIC 39\boldsymbol{)}$ |
| --- | --- | --- | --- |

This means that the number is entered in the first component of the vector $\boldsymbol{b}$, which corresponds to the population aged 0-4 years, while the remaining 20 values are zero.

Time-varying intervention intensity functions

*Note*. All variables below are defined for each time $t$. The corresponding name in bold letters refers to the vector for all the times.

Let $X_{CTE}$ be the set of times $t$ at which the intervention is applied, specifically shielding the elderly, and $v_{t}^{\left( CTE \right)}$ denote the coverage of protected elderly individuals:

|  | $coc(t)=\left\{ \begin{matrix} v_{t}^{\left( CTE \right)} & if & t\in X_{CTE} \\ & & \\ 0 & if & t\notin X_{CTE} \end{matrix} \right..$ | $\boldsymbol{(} SEQ Ecuación \backslash* ARABIC 40\boldsymbol{)}$ |
| --- | --- | --- |

Let $X_{SI}$ be the set of times $t$ at which the intervention is applied, self-isolation if symptomatic, and $v_{t}^{\left( SI \right)}$ the proportion of symptomatic individuals who self-isolate:

|  | $self(t)=\left\{ \begin{matrix} v_{t}^{\left( SI \right)} & if & t\in X_{SI} \\ & & \\ 0 & if & t\notin X_{SI} \end{matrix} \right..$ | $( SEQ Ecuación \backslash* ARABIC 41)$ |
| --- | --- | --- |

Let $X_{SCR}$ be the set of times $t$ at which the intervention is applied, (self-isolation) screening, and $v_{t}^{\left( SCR \right)}$ the number of contacts screen:

|  | $s_{cr}(t)=\left\{ \begin{matrix} v_{t}^{\left( SCR \right)} & if & t\in X_{SCR} \\ & & \\ 0 & if & t\notin X_{SCR} \end{matrix} \right..$ | $\boldsymbol{(} SEQ Ecuación \backslash* ARABIC 42\boldsymbol{)}$ |
| --- | --- | --- |

Let $X_{SC}$ be the set of times $t$ at which the intervention is applied, school closures, and $v_{t}^{\left( SC \right)}$ the reduction of contacts in school upon school closure:

|  | $sc_{eff}(t)=\left\{ \begin{matrix} v_{t}^{\left( SC \right)} & if & t\in X_{SC} \\ & & \\ 0 & if & t\notin X_{SC} \end{matrix} \right..$ | $\boldsymbol{(} SEQ Ecuación \backslash* ARABIC 43\boldsymbol{)}$ |
| --- | --- | --- |

Let $X_{SCP}$ be the set of times $t$ at which the intervention is applied, partial school closures, and $v_{t}^{\left( SCP \right)}$ the reduction of school contacts in case of partial school closure:

|  | $sc_{effp}(t)=\left\{ \begin{matrix} v_{t}^{\left( SCP \right)} & if & t\in X_{SCP} \\ & & \\ 0 & if & t\notin X_{SCP} \end{matrix} \right..$ | $\boldsymbol{(} SEQ Ecuación \backslash* ARABIC 44\boldsymbol{)}$ |
| --- | --- | --- |

Let $X_{SD}$ be the set of times $t$ at which the intervention is applied, social distancing, and $v_{t}^{\left( SD \right)}$ the adherence to social distancing in community level:

|  | $d_{cov}(t)=\left\{ \begin{matrix} v_{t}^{\left( SD \right)} & if & t\in X_{SD} \\ & & \\ 0 & if & t\notin X_{SD} \end{matrix} \right.,$ | $\boldsymbol{(} SEQ Ecuación \backslash* ARABIC 45\boldsymbol{)}$ |
| --- | --- | --- |
|  | $d(t)=\left\{ \begin{matrix} 1 & if & t\in X_{SD} \\ & & \\ 0 & if & t\notin X_{SD} \end{matrix} \right..$ |  |

Let $X_{HW}$ be the set of times $t$ at which the intervention is applied, handwashing, and $v_{t}^{\left( HW \right)}$ the adherence to handwashing:

|  | $h_{cov}(t)=\left\{ \begin{matrix} v_{t}^{\left( HW \right)} & if & t\in X_{HW} \\ & & \\ 0 & if & t\notin X_{HW} \end{matrix} \right..$ | $\boldsymbol{(} SEQ Ecuación \backslash* ARABIC 46\boldsymbol{)}$ |
| --- | --- | --- |

Let $X_{MSK}$ be the set of times $t$ at which the intervention is applied, mask wearing, and $v_{t}^{\left( MSK \right)}$ the adherence to mask wearing:

|  | $m_{cov}(t)=\left\{ \begin{matrix} v_{t}^{\left( MSK \right)} & if & t\in X_{MSK} \\ & & \\ 0 & if & t\notin X_{MSK} \end{matrix} \right..$ | $\boldsymbol{(} SEQ Ecuación \backslash* ARABIC 47\boldsymbol{)}$ |
| --- | --- | --- |

Let $X_{WAH}$ be the set of times $t$ at which the intervention is applied, working at home, and $v_{t}^{\left( WAH \right)}$ the adherence to work from home policies:

|  | $w_{cov}(t)=\left\{ \begin{matrix} v_{t}^{\left( WAH \right)} & if & t\in X_{WAH} \\ & & \\ 0 & if & t\notin X_{WAH} \end{matrix} \right.,$ | $\boldsymbol{(} SEQ Ecuación \backslash* ARABIC 48\boldsymbol{)}$ |
| --- | --- | --- |
|  | $wh(t)=\left\{ \begin{matrix} 1 & if & t\in X_{WAH} \\ & & \\ 0 & if & t\notin X_{WAH} \end{matrix} \right..$ |  |

Let $X_{TB}$ be the set of times $t$ at which the intervention is applied, international travel ban, and $v_{t}^{\left( TB \right)}$ the efficacy of interrupting the flow of trips out of the country and isolating cases from abroad:

|  | $tb_{eff}(t)=\left\{ \begin{matrix} v_{t}^{\left( TB \right)} & if & t\in X_{TB} \\ & & \\ 0 & if & t\notin X_{TB} \end{matrix} \right..$ | $\boldsymbol{(} SEQ Ecuación \backslash* ARABIC 49\boldsymbol{)}$ |
| --- | --- | --- |

Let $X_{VAC}$ be the set of times $t$ at which the intervention is applied, vaccination, and $v_{t}^{\left( VAC \right)}$ the adherence to vaccination:

|  | $vac_{cov}(t)=\left\{ \begin{matrix} v_{t}^{\left( VAC \right)} & if & t\in X_{VAC} \\ & & \\ 0 & if & t\notin X_{VAC} \end{matrix} \right..$ | $\boldsymbol{(} SEQ Ecuación \backslash* ARABIC 50\boldsymbol{)}$ |
| --- | --- | --- |

Let $X_{Q}$ be the set of times $t$ at which the intervention is applied, (self-isolation) household isolation, and $v_{t}^{\left( Q \right)}$ the adherence to quarantine:

|  | $q_{cov}\left( t \right)=\left\{ \begin{matrix} v_{t}^{\left( Q \right)} & if & t\in X_{Q} \\ & & \\ 0 & if & t\notin X_{Q} \end{matrix} \right.,$ | $\boldsymbol{(} SEQ Ecuación \backslash* ARABIC 51\boldsymbol{)}$ |
| --- | --- | --- |
|  | $q(t)=\left\{ \begin{matrix} 1 & if & t\in X_{Q} \\ & & \\ 0 & if & t\notin X_{Q} \end{matrix} \right..$ |  |

Let $X_{MT}$ be the set of times $t$ at which the intervention is applied, mass testing, and $v_{t}^{\left( MT \right)}$ the amount of people tested in one day, given in thousand:

|  | ${test}_{pd} (t)=\left\{ \begin{matrix} v_{MT} & if & t\in X_{MT} \\ & & \\ 0 & if & t\notin X_{MT} \end{matrix} \right..$ | $\boldsymbol{(} SEQ Ecuación \backslash* ARABIC 52\boldsymbol{)}$ |
| --- | --- | --- |

Let $X_{DMOD}$ be the set of times *t* at which the variant(s) is present and $v_{t}^{\left( DMOD \right)}$ the value corresponding to the lethality:

|  | $dm(t)=\left\{ \begin{matrix} v_{t}^{\left( DMOD \right)} & if & t\in X_{DMOD} \\ & & \\ 0 & if & t\notin X_{DMOD} \end{matrix} \right..$ | $\boldsymbol{(} SEQ Ecuación \backslash* ARABIC 53\boldsymbol{)}$ |
| --- | --- | --- |

Let $X_{PMOD}$ be the set of times $t$ at which the variant(s) are present and $v_{t}^{\left( PMOD \right)}$ the value corresponding to transmissibility:

|  | $pm(t)=\left\{ \begin{matrix} v_{t}^{\left( PMOD \right)} & if & t\in X_{PMOD} \\ & & \\ 0 & if & t\notin X_{PMOD} \end{matrix} \right..$ | $\boldsymbol{(} SEQ Ecuación \backslash* ARABIC 54\boldsymbol{)}$ |
| --- | --- | --- |

Let $X_{CMOD}$ be the set of times $t$ at which the variant(s) are present and $v_{t}^{\left( CMOD \right)}$ the value corresponding to the breakthrough infection probability:

|  | $\sigma_{Rmod}(t)=\left\{ \begin{matrix} v_{t}^{\left( CMOD \right)} & if & t\in X_{CMOD} \\ & & \\ \sigma_{R} & if & t\notin X_{CMOD} \end{matrix} \right..$ | $\boldsymbol{(} SEQ Ecuación \backslash* ARABIC 55\boldsymbol{)}$ |
| --- | --- | --- |

Let $X_{DEX}$ be the set of times *t* at which the variant(s) are present and $1$ the corresponding value if dexamethasone is applied:

|  | $d_{x}(t)=\left\{ \begin{matrix} 1 & if & t\in X_{DEX} \\ & & \\ 0 & if & t\notin X_{DEX} \end{matrix} \right..$ | $\boldsymbol{(} SEQ Ecuación \backslash* ARABIC 56\boldsymbol{)}$ |
| --- | --- | --- |

This last indicator allows for modifications to parameters associated with the application of dexamethasone. The conditions for these modifications are:

|  | If $d_{x}(t) = 1$ then $p_{v}=p_{v}\cdot v_{d}.$ | $\boldsymbol{(} SEQ Ecuación \backslash* ARABIC 57\boldsymbol{)}$ |
| --- | --- | --- |
|  | If $d_{x}(t) = 0$ then $d_{2}=d_{2c}=d_{v}=d_{vc}=1.$ |  |

Proportion of hospitalised people requiring assistive or ICU beds or ventilators

The *splinefun* function allows to interpolate the desired value by pre-setting $n(x,y)$ pairs, the computation is done based on the hyman method (Hyman, 2006):

|  | $c =$ | $\min\left( 1-splinefun_{ICU}\left( \sum\boldsymbol{ICU}+\sum\boldsymbol{Vent}+\sum\boldsymbol{VentC} \right), 1 \right),$ | $\boldsymbol{(} SEQ Ecuación \backslash* ARABIC 58\boldsymbol{)}$ |
| --- | --- | --- | --- |
|  | $c_{H}=$ | $\min\left( 1-splinefun_{H}\left( \sum\boldsymbol{H}+\sum\boldsymbol{ICUC}+\sum\boldsymbol{ICUCV} \right), 1 \right)$, |  |
|  | $c_{V} =$ | $\min\left( 1-splinefun_{Vent}\left( \sum\boldsymbol{Vent} \right), 1 \right).$ |  |

Total population per age group

|  | $\boldsymbol{P=}$ | $\boldsymbol{S+SR + E+I+R+X+Z+V+H+HC+ICU+ICUC+ICUCV+Vent+}$ | $\boldsymbol{(} SEQ Ecuación \backslash* ARABIC 59\boldsymbol{)}$ |
| --- | --- | --- | --- |
|  |  | $\boldsymbol{VentC+EV+ER+EVR+VR+HCICU+HCV+QS+QSR + QE+QI+}$ |  |
|  |  | $\boldsymbol{QR+CL+QC+QEV+QV+QER+QEVR+QVR}$. |  |

Quarantine rate

|  | $Q\boldsymbol{=}$ | $\left( \sum\boldsymbol{QS}+\sum\boldsymbol{QSR}+\sum\boldsymbol{QE}+\sum\boldsymbol{QI}+\sum\boldsymbol{QC}+\sum\boldsymbol{QR}+\sum\boldsymbol{QV}+\sum\boldsymbol{QER}+\sum\boldsymbol{QEVR}+\sum\boldsymbol{QEV}+\sum\boldsymbol{QVR} \right)/\sum\boldsymbol{P},$ |  |
| --- | --- | --- | --- |
|  | $rate_{q}(t)\boldsymbol{=}$ | $\min\left( \min\left( \sum\left( \boldsymbol{CL}+\boldsymbol{H}+\boldsymbol{ICU}+\boldsymbol{Vent}+\boldsymbol{HC}+\boldsymbol{ICUC}+\boldsymbol{ICUCV}+\boldsymbol{VentC}+\boldsymbol{HCV}+\boldsymbol{HCICU} \right)\cdot\frac{\left( hs-1 \right)}{\sum\boldsymbol{P}},1 \right)\cdot qe,q_{cov}(t)/2 \right)\boldsymbol{,}$ | $\boldsymbol{(} SEQ Ecuación \backslash* ARABIC 60\boldsymbol{)}$ |
|  | $qr(t)\boldsymbol{=}$ | ${rate}_{q}(t)\boldsymbol{/}\left( 1+\exp\left( -10\cdot\left( q_{cov}(t)/2-Q \right) \right) \right)$. |  |

Test rate

If the total population of each compartment is greater than one at a specific time, the parameter will be adjusted accordingly; otherwise, it will be zero:

|  | ${rt}_{\left( \boldsymbol{\Xi} \right)}(t)\boldsymbol{=}$ | $\frac{m_{tests}\cdot{test}_{pd}(t)}{\sum\boldsymbol{P}}, \boldsymbol{\Xi} \in\{\boldsymbol{C, E, ER, EV, EVR, HC, HCICU, HCV, I}\}\boldsymbol{.}$ | $\boldsymbol{(} SEQ Ecuación \backslash* ARABIC 61\boldsymbol{)}$ |
| --- | --- | --- | --- |

Rates for self-isolation, homework, vaccination, handwashing, social distancing and mask wearing for the defined period

|  | $s_{eff}(t)\boldsymbol{=}$ | $\min\left( \sum\left( r\cdot\boldsymbol{I}+r_{c}\cdot\boldsymbol{CL}+\boldsymbol{H}+\boldsymbol{ICU}+\boldsymbol{Vent}+r_{h}\cdot\left( \boldsymbol{HC}+\boldsymbol{ICUC}+\boldsymbol{ICUCV}+\boldsymbol{VentC}+\boldsymbol{HCICU}+\boldsymbol{HCV} \right) \right)\cdot s_{cr}(t)\cdot\left( s_{over}\cdot\boldsymbol{I⊘P} \right)\cdot s_{tests}\boldsymbol{⊘P},1 \right),$ | $\boldsymbol{(} SEQ Ecuación \backslash* ARABIC 62\boldsymbol{)}$ |
| --- | --- | --- | --- |
|  | $work(t)\boldsymbol{=}$ | $w_{eff}\cdot w_{cov}(t),$ |  |
|  | $vac(t)\boldsymbol{=}$ | $\frac{\boldsymbol{-}\log\left( 1-vac_{cov}(t) \right)}{vac_{c}},$ |  |
|  | $hand(t)\boldsymbol{=}$ | $h_{eff}\cdot h_{cov}(t),$ |  |
|  | $dist(t)\boldsymbol{=}$ | $d_{eff}\cdot d_{cov}(t),$ |  |
|  | $mask(t)\boldsymbol{=}$ | $m_{eff}\cdot m_{cov}(t).$ |  |

Force of infection

Next, we define the force of infection in the overall community, denoted by $\boldsymbol{\lambda}$, and the force of infection for quarantined individuals, denoted by $\boldsymbol{\varrho.}$ These forces are modulated by reductions of contacts due to quarantine, shielding of the elderly, handwashing, masking, and school closures. Here, the $\boldsymbol{max}$ function returns the maximum value for each element between two vectors.

|  | $\boldsymbol{schoolclose}\boldsymbol{3}(t)\boldsymbol{=}$ | $\max\left( sc_{eff}(t)\cdot\boldsymbol{1}_{\boldsymbol{scc}}(t)\boldsymbol{,}sc_{effp}(t)\cdot\boldsymbol{1}_{\boldsymbol{sccp}}(t) \right)$, | $\boldsymbol{(} SEQ Ecuación \backslash* ARABIC 63\boldsymbol{)}$ |
| --- | --- | --- | --- |
|  | $\boldsymbol{schoolclose}\boldsymbol{4}(t)\boldsymbol{=}$ | $\boldsymbol{max}((1-{sc}_{eff}(t)\boldsymbol{)}\cdot\boldsymbol{1}_{\boldsymbol{scc}}(t)\boldsymbol{,}\left( 1-sc_{effp}(t) \right)\cdot\boldsymbol{1}_{\boldsymbol{sccp}}(t)\boldsymbol{),}$ |  |
|  | $\boldsymbol{Cts}(t)\boldsymbol{=}$ | $(\boldsymbol{W}_{\boldsymbol{h}}\boldsymbol{+}d\left( t \right)\cdot\left( 1-dist\left( t \right) \right)\cdot\boldsymbol{W}_{\boldsymbol{o}}\boldsymbol{+}\left( 1-d\left( t \right) \right)\cdot\boldsymbol{W}_{\boldsymbol{o}}$  $\boldsymbol{+}\left( \boldsymbol{1-schoolclose}\boldsymbol{3}\left( t \right) \right)\boldsymbol{\odot}\boldsymbol{W}_{\boldsymbol{s}}$  $\boldsymbol{+ schoolclose}\boldsymbol{4}\left( t \right)\boldsymbol{\odot}\boldsymbol{W}_{\boldsymbol{s}}$  $\boldsymbol{+}\left( \boldsymbol{schoolclose}\boldsymbol{3}\left( t \right)\boldsymbol{\odot}\boldsymbol{W}_{\boldsymbol{h}} \right))\cdot s_{2h}\boldsymbol{+}\left( 1-wh\left( t \right) \right)\cdot\boldsymbol{W}_{\boldsymbol{w}}$  $\boldsymbol{+}wh\left( t \right)\cdot\left( 1-work\left( t \right) \right)\cdot\boldsymbol{W}_{\boldsymbol{w}}$  $\boldsymbol{+}\boldsymbol{W}_{\boldsymbol{h}}\cdot wh(t)\cdot work(t)\cdot w_{2h}$ , |  |
|  |  |  |  |
|  |  |  |  |
|  | $seas(t) =$ | $1+\zeta\cdot cos\left( 2\cdot3.14\cdot\left( t-\left( \phi\cdot365.25/12 \right) \right)/365.25 \right)$, |  |
|  | $importation(t)\boldsymbol{=}$ | $imp\cdot\left( 1-tb_{eff}(t) \right)$, |  |
|  | $\boldsymbol{HH =}$ | $\boldsymbol{H+ICU+Vent+ICUC+ICUCV+VentC}$, |  |
|  | $\boldsymbol{HHC =}$ | $\boldsymbol{HC+HCICU+HCV}$, |  |
|  | $\boldsymbol{W}_{\boldsymbol{coc}}\boldsymbol{=}$ | $\left( \begin{matrix} \boldsymbol{J}_{a_{coc}} & (1-{coc}_{eff})\boldsymbol{1}_{21-a_{coc}} \\ (1-{coc}_{eff})\boldsymbol{1'}_{21-a_{coc}} & (1-{coc}_{eff}) \end{matrix} \right),$ |  |
|  | $\boldsymbol{Contacts}(t)\boldsymbol{=}$ | $\left( 1-coc\left( t \right) \right)\cdot\boldsymbol{Cts}\left( t \right)\boldsymbol{+}coc\left( t \right)\cdot\boldsymbol{Cts}(t)\boldsymbol{\odot}\boldsymbol{W}_{\boldsymbol{coc}}$  $\boldsymbol{+}\left( coc(t)\cdot\left( \boldsymbol{1+schoolclose}\boldsymbol{3}(t)\cdot\left( 1-sc_{eff}(t) \right)\boldsymbol{+}wh(t)\cdot\left( 1-w_{eff} \right) \right)\boldsymbol{\odot}\boldsymbol{W}_{\boldsymbol{h}} \right)\boldsymbol{\odot}\left( \boldsymbol{J-}\boldsymbol{W}_{\boldsymbol{coc}} \right).$ |  |

Then,

|  | $\boldsymbol{\lambda}(t)\boldsymbol{=}$ | $\left( 1\boldsymbol{-}\max\left( hand\left( t \right)\boldsymbol{,}mask\left( t \right) \right) \right)\cdot\left( p\cdot pm\left( t \right) \right)\cdot seas\left( t \right)$  $\cdot\left( \boldsymbol{Contacts}(t)\cdot\left( \left( \rho\cdot\boldsymbol{E+}\left( \boldsymbol{I+CL+}importation(t) \right)\boldsymbol{+}\left( 1-self_{eff} \right)\cdot\left( \boldsymbol{X+HHC} \right)\boldsymbol{+}\rho_{s}\cdot\left( \boldsymbol{HH} \right) \right)\boldsymbol{⊘P} \right) \right)\boldsymbol{+}$  $\left( 1-\max\left( hand\left( t \right),mask\left( t \right) \right) \right)\cdot\left( p\cdot pm\left( t \right) \right)\cdot seas\left( t \right)\cdot\left( 1\boldsymbol{-}q\left( t \right)\cdot q_{effo} \right)$  $\cdot\left( \boldsymbol{W}_{\boldsymbol{o}}\cdot\left( \left( \rho\cdot\boldsymbol{QE+QI+QC+QEV+QEVR+QER} \right)\boldsymbol{⊘P} \right) \right),$ | $\boldsymbol{(} SEQ Ecuación \backslash* ARABIC 64\boldsymbol{)}$ |
| --- | --- | --- | --- |
|  | $\boldsymbol{\varrho}(t)\boldsymbol{=}$ | $\left( 1-\max\left( hand\left( t \right)\boldsymbol{,}mask\left( t \right) \right) \right)\cdot\left( p\cdot pm\left( t \right) \right)\cdot seas\left( t \right)\cdot\left( 1-q_{effh} \right)$  $\cdot\boldsymbol{W}_{\boldsymbol{h}}\cdot\left( \left( \left( 1-self_{eff} \right)\cdot\left( \boldsymbol{X+HHC+}\rho\cdot\boldsymbol{QE+QI+QC+QEV+QEVR+QER} \right) \right)\boldsymbol{⊘P} \right)\boldsymbol{+}$  $\left( 1\boldsymbol{-}\max\left( hand\left( t \right)\boldsymbol{,}mask\left( t \right) \right) \right)\cdot\left( p\cdot pm\left( t \right) \right)\cdot seas\left( t \right)\cdot\left( 1-q_{effo} \right)$  $\cdot\left( \boldsymbol{W}_{\boldsymbol{o}}\cdot\left( \left( \rho\cdot\boldsymbol{E+}\left( \boldsymbol{I+CL+}importation\left( t \right) \right)\boldsymbol{+}\left( 1-self_{eff} \right)\cdot\left( \boldsymbol{X+HHC+}\rho\cdot\boldsymbol{QE+QI+QC+QEV+QEVR+QER} \right)\boldsymbol{+}\rho_{s}\cdot\boldsymbol{HH} \right)\boldsymbol{⊘P} \right) \right)\boldsymbol{.}$ |  |

Initial conditions

We initialize every compartment to zero except for **S**, **E** and **R.** We seed an initial number of exposed individuals, given by *init*, in the age class *ageinit*. For Bogotá, infections are initially set in age group 4, corresponding to ages 15 to 19. Additionally, a *pre* proportion of the total population **N** is set to be naturally immune at time zero:

|  | $\boldsymbol{E}_{0,ageinit}=$ | $init,$ | $\boldsymbol{(} SEQ Ecuación \backslash* ARABIC 65\boldsymbol{)}$ |
| --- | --- | --- | --- |
|  | $\boldsymbol{R}_{0,.}=$ | $pre\cdot\boldsymbol{N,}$ |  |
|  | $\boldsymbol{S}_{0,.}=$ | $\boldsymbol{N -}\boldsymbol{E}_{0,.}\boldsymbol{-}\boldsymbol{R}_{0,.}$ |  |

E. Parameter sampling and uncertainty analysis

While the benchmark model is deterministic in nature, we have integrated stochasticity to account for uncertainty in a specific set of inputs, denoted as $p_{noise}$. For each model iteration, Gaussian noise is added to the parameters in the $p_{noise}$ vector, following $\mathcal{N}\left( 0, noise\cdot\left| p_{noise} \right| \right).$ The mean of this distribution corresponds to the default parameter value used in the deterministic model, while the standard deviation is given by parameter *noise*. The parameters included in $p_{noise}$ are $p, \rho, \omega, \gamma,\nu, ihr_{s}, \nu_{s} , \nu_{icu}, \nu_{v}, \rho_{s}, self_{eff}, d_{eff}, h_{eff}, m_{eff}, w_{eff}, w_{2h}, s_{2h}, coc_{eff}, imp, s_{over}, qe, q_{effh}$ and $q_{effo}$.

F. Epidemiological metrics

Incidence of daily cases

The daily incidence of cases is calculated as the sum of non-hospitalised cases ($\boldsymbol{incidence}$**)** and hospitalised cases ($\boldsymbol{incidenceh}$**)**. The values for these compartments are derived from the solution of the ODE system for the compartments **E**, **Q**, **QE**, **QE**, **EV**, **EVR** and **ER**, each representing 21 age groups for each time $t,$ ranging from 1 to a final time $t_{f}$ established by the study. Formally, the parameters influencing the daily case incidence are:

| $\boldsymbol{incidence =}$ | $r\cdot\gamma\cdot\left( 1-p_{c} \right)\cdot\boldsymbol{E}\cdot\left( \boldsymbol{1-ihr} \right)$  $\boldsymbol{+}r_{c}\cdot\gamma\cdot p_{c}\cdot\boldsymbol{E}\cdot\left( \boldsymbol{1-ihr} \right)$  $\boldsymbol{+}r\cdot\gamma\cdot\left( 1-p_{c} \right)\cdot\boldsymbol{QE}\cdot\left( \boldsymbol{1-ihr} \right)$  $\boldsymbol{+}r_{c}\cdot\gamma\cdot p_{c}\cdot\boldsymbol{QE}\cdot\left( \boldsymbol{1-ihr} \right)$  $\boldsymbol{+}r_{v}\cdot\gamma\cdot\left( 1-p_{cv} \right)\cdot\boldsymbol{EV}\cdot\left( \boldsymbol{1-}\sigma_{EV}\cdot\boldsymbol{ihr} \right)$  $\boldsymbol{+}r_{cv}\cdot\gamma\cdot p_{cv}\cdot\boldsymbol{EV}\cdot\left( \boldsymbol{1-}\sigma_{EV}\cdot\boldsymbol{ihr} \right)$  $\boldsymbol{+}r_{vr}\cdot\gamma\cdot\left( 1-p_{cvr} \right)\cdot\boldsymbol{EVR}\cdot\left( \boldsymbol{1-}\sigma_{EVR}\cdot\boldsymbol{ihr} \right)$  $\boldsymbol{+}r_{cvr}\cdot\gamma\cdot p_{cvr}\cdot\boldsymbol{EVR}\cdot\left( \boldsymbol{1-}\sigma_{EVR}\cdot\boldsymbol{ihr} \right)$  $\boldsymbol{+}r_{r}\cdot\gamma\cdot\left( 1-p_{cr} \right)\cdot\boldsymbol{ER}\cdot\left( \boldsymbol{1-}\sigma_{ER}\cdot\boldsymbol{ihr} \right)$  $\boldsymbol{+}r_{cr}\cdot\gamma\cdot p_{cr}\cdot\boldsymbol{ER}\cdot\left( \boldsymbol{1 -}\sigma_{ER}\cdot\boldsymbol{ihr} \right)\boldsymbol{,}$ | $\boldsymbol{(} SEQ Ecuación \backslash* ARABIC 66\boldsymbol{)}$ |
| --- | --- | --- |
| $\boldsymbol{incidenceh =}$ | $\gamma\cdot\boldsymbol{E}\cdot\boldsymbol{ihr}\cdot\left( 1-c_{H} \right)\cdot\left( 1-p_{icu} \right)\cdot r_{h}$  $\boldsymbol{+}\gamma\cdot\boldsymbol{E}\cdot\boldsymbol{ihr}\cdot\left( 1-c_{H} \right)\cdot\left( 1-p_{icu} \right)\cdot\left( 1-r_{h} \right)\cdot r_{g}$  $\boldsymbol{+}\gamma\cdot\boldsymbol{QE}\cdot\boldsymbol{ihr}\cdot\left( 1-c_{H} \right)\cdot\left( 1-p_{icu} \right)\cdot r_{h}$  $\boldsymbol{+}\gamma\cdot\boldsymbol{QE}\cdot\boldsymbol{ihr}\cdot\left( 1-c_{H} \right)\cdot\left( 1-p_{icu} \right)\cdot\left( 1-r_{h} \right)\cdot r_{g}$  $\boldsymbol{+}\gamma\cdot\sigma_{EV}\cdot\boldsymbol{EV}\cdot\boldsymbol{ihr}\cdot\left( 1-c_{H} \right)\cdot\left( 1-p_{icuv} \right)\cdot r_{h}$  $\boldsymbol{+}\gamma\cdot\sigma_{EVR}\cdot\boldsymbol{EVR}\cdot\boldsymbol{ihr}\cdot\left( 1-c_{H} \right)\cdot\left( 1-p_{icuvr} \right)\cdot r_{h}$  $\boldsymbol{+}\gamma\cdot\sigma_{ER}\cdot\boldsymbol{ER}\cdot\boldsymbol{ihr}\cdot\left( 1-c_{H} \right)\cdot\left( 1-p_{icur} \right)\cdot r_{h}$  $\boldsymbol{+}\gamma\cdot\boldsymbol{E}\cdot\boldsymbol{ihr}\cdot c_{H}\cdot r_{g}\cdot\left( 1-p_{icu} \right)$  $\boldsymbol{+}\gamma\cdot\boldsymbol{QE}\cdot\boldsymbol{ihr}\cdot c_{H}\cdot r_{g}\cdot\left( 1-p_{icu} \right)$  $\boldsymbol{+}\gamma\cdot\sigma_{EV}\cdot\boldsymbol{EV}\cdot\boldsymbol{ihr}\cdot c_{H}\cdot r_{g}\cdot\left( 1-p_{icuv} \right)$  $+ \gamma\cdot\sigma_{EVR}\cdot\boldsymbol{EVR}\cdot\boldsymbol{ihr}\cdot c_{H}\cdot r_{g}\cdot\left( 1-p_{icuvr} \right)$  $\boldsymbol{+}\gamma\cdot\sigma_{ER}\cdot\boldsymbol{ER}\cdot\boldsymbol{ihr}\cdot c_{H}\cdot r_{g}\cdot\left( 1-p_{icur} \right)$  $\boldsymbol{+}\gamma\cdot\boldsymbol{E}\cdot\boldsymbol{ihr}\cdot p_{icu}\cdot\left( 1-c \right)\cdot r_{hICU}$  $\boldsymbol{+}\gamma\cdot\boldsymbol{QE}\cdot\boldsymbol{ihr}\cdot p_{icu}\cdot\left( 1-c \right)\cdot r_{hICU}$  $+ \gamma\cdot\boldsymbol{E}\cdot\boldsymbol{ihr}\cdot p_{icu}\cdot c\cdot r_{hICU}\cdot r_{g}$  $+ \gamma\cdot\boldsymbol{QE}\cdot\boldsymbol{ihr}\cdot p_{icu}\cdot c\cdot r_{hICU}\cdot r_{g}$  $\boldsymbol{+}\gamma\cdot\sigma_{EV}\cdot\boldsymbol{EV}\cdot\boldsymbol{ihr}\cdot\left( 1-c \right)\cdot p_{icuv}\cdot r_{hICU}$  $\boldsymbol{+}\gamma\cdot\sigma_{EVR}\cdot\boldsymbol{EVR}\cdot\boldsymbol{ihr}\cdot\left( 1-c \right)\cdot p_{icuvr}\cdot r_{hICU}$  $\boldsymbol{+}\gamma\cdot\sigma_{ER}\cdot\boldsymbol{ER}\cdot\boldsymbol{ihr}\cdot\left( 1-c \right)\cdot p_{icur}\cdot r_{hICU}$  $+ \gamma\cdot\sigma_{EV}\cdot\boldsymbol{EV}\cdot\boldsymbol{ihr}\cdot c\cdot p_{icuv}\cdot r_{hICU}\cdot r_{g}$  $+ \gamma\cdot\sigma_{EVR}\cdot\boldsymbol{EVR}\cdot\boldsymbol{ihr}\cdot c\cdot p_{icuvr}\cdot r_{hICU}\cdot r_{g}$  $\boldsymbol{+}\gamma\cdot\sigma_{ER}\cdot\boldsymbol{ER}\cdot\boldsymbol{ihr}\cdot c\cdot p_{icur}\cdot r_{hICU}\cdot r_{g}$  $+ \gamma\cdot\boldsymbol{E}\cdot\boldsymbol{ihr}\cdot p_{icu}\cdot\left( 1-r_{hICU} \right)\cdot r_{g}$  $+ \gamma\cdot\boldsymbol{QE}\cdot\boldsymbol{ihr}\cdot p_{icu}\cdot\left( 1-r_{hICU} \right)\cdot r_{g}$  $\boldsymbol{+}\gamma\cdot\sigma_{EV}\cdot\boldsymbol{EV}\cdot\boldsymbol{ihr}\cdot p_{icuv}*\left( 1-r_{hICU} \right)\cdot r_{g}$  $+ \gamma\cdot\sigma_{EVR}\cdot\boldsymbol{EVR}\cdot\boldsymbol{ihr}\cdot p_{icuvr}*\left( 1-r_{hICU} \right)\cdot r_{g}$  $+ \gamma\cdot\sigma_{ER}\cdot\boldsymbol{ER}\cdot\boldsymbol{ihr}\cdot p_{icur}*\left( 1 - r_{hICU} \right)\cdot r_{g},$ |  |
|  |  |  |
| $\boldsymbol{cases=}$ | $\boldsymbol{incidence +incidenceh.}$ |  |

Incidence of daily infections

The number of daily infections is related to the compartments **E**, **QE**, and **EV** compartments and the parameter $\gamma$. Note that $\lfloor\cdot\rfloor$ denotes the floor function, and $\boldsymbol{G}_{t,j}$ represents the element of the matrix $\boldsymbol{G}$ at position $t,j$, where $t$ is the row and $j$ is the column.

| $\boldsymbol{G=}$ | $\gamma\cdot\sum_{\boldsymbol{\Xi\in}\Omega} \boldsymbol{\Xi,}\Omega\boldsymbol{= \{E, QE, EV\}}\boldsymbol{,}$ | $\boldsymbol{(} SEQ Ecuación \backslash* ARABIC 67\boldsymbol{)}$ |
| --- | --- | --- |
| $di(t)\boldsymbol{=}$ | $\sum_{j=1}^{21} \boldsymbol{G}_{t,j}, t\in\left\{ 1,\ldots,t_{f} \right\},$ |  |
| $\boldsymbol{di=}$ | $\left( \begin{matrix} di(1) \\ \begin{matrix} \vdots\\ \begin{matrix} di(t) \\ \vdots\\ di(t_{f}) \end{matrix} \end{matrix} \end{matrix} \right), t\in\left\{ 1,\ldots,t_{f} \right\},$ |  |
| $\boldsymbol{day\_infections=}$ | $\left\{ \begin{matrix} \left\lfloor\boldsymbol{di}\left. +0.5 \right\rfloor\right. & if & \forall t,di(t)\geq0 \\ & & \\ \boldsymbol{0} & if & \forall t, di(t)<0 \end{matrix}. \right.$ |  |

Effective reproductive number

The basic reproductive number at time $t$ (or effective reproductive number) $R_{t}$ is estimated as the ratio of the total number of exposed persons (across 21 age groups) at time$t$ to the total number exposed at time $t-\frac{1}{\nu}$. In the case of $R_{t}\geq7$, it is not assigned a value and is represented in the algorithm as $\mathrm{NA}$. For each $R_{t}$, there is an iteration $i$. Note that $t\in\left\{ \left\lceil\frac{1}{\nu} \right\rceil,..., n_{t} \right\}$, where $\lceil\cdot\rceil$ is the ceiling function, and $t_{f}$ is the end date of the study. The parameters $\nu$and $\gamma$ depend on whether the number of iterations is greater than one, and $\boldsymbol{E}_{t,j}$ represents the value of the compartment at time $t$ in age group $j$.

| $E_{t}\boldsymbol{=}$ | $\gamma\cdot\sum_{j=1}^{21} \boldsymbol{E}_{t,j} ,$ | $\boldsymbol{(} SEQ Ecuación \backslash* ARABIC 68\boldsymbol{)}$ |
| --- | --- | --- |
| $E_{t\boldsymbol{-}\frac{1}{\nu}}\boldsymbol{=}$ | $\gamma\cdot\sum_{j=1}^{21} \boldsymbol{E}_{t\boldsymbol{-}\frac{1}{\nu},j},$ |  |
| $R_{t}\boldsymbol{=}$ | $\left\{ \begin{matrix} \frac{E_{t}}{E_{t\boldsymbol{-}\frac{1}{\nu}}} & if & t\in\left\{ \left( \left\lceil\frac{1}{\nu} \right\rceil+ 1 \right),\ldots,t_{f} \right\} \\ 0 & if & t \left\{ \left( \left\lceil\frac{1}{\nu} \right\rceil+ 1 \right),\ldots,t_{f} \right\} \\ NA & if & \frac{E_{t}}{E_{t\boldsymbol{-}\frac{1}{\nu}}}>7 \end{matrix}\boldsymbol{.} \right.$ |  |

Hospital occupancy, capacity and cumulative deaths reported

Using equation $(67)$ and substituting $\gamma=1$, we obtain: i) surge bed occupancy when $\Omega=\{\boldsymbol{H},\boldsymbol{ICUC},\boldsymbol{ICUCV}\};$ ii) ICU bed occupancy when $\Omega=\{\boldsymbol{ICU}, \boldsymbol{VentC}\}$; iii) ventilator critical patient occupancy when $\Omega=\{\boldsymbol{Vent}\}$; iv) number of (non-ICU) beds required when $\Omega=\{\boldsymbol{H}, \boldsymbol{HC}\}$; v) ICU bed capacity needed when $\Omega=\{\boldsymbol{ICU}, \boldsymbol{ICUC}, \boldsymbol{HCICU}\}$; vi) number of ICU beds with ventilator needed when $\Omega=\left\{ \boldsymbol{Vent}, \boldsymbol{VentC}, \boldsymbol{HCV}, \boldsymbol{ICUCV} \right\}$; and vii) cumulative deaths reported when $\Omega=\{\boldsymbol{CM}\}$.

Cumulative deaths

We can compute all types of cumulative deaths (reported or reported + unreported) as follows:

|  | $\boldsymbol{mort}\left( \boldsymbol{\Xi,}j \right)\boldsymbol{=}f_{j}\cdot\boldsymbol{\Xi}\cdot\boldsymbol{z},$ | $\boldsymbol{(} SEQ Ecuación \backslash* ARABIC 69\boldsymbol{)}$ |
| --- | --- | --- |

where $\boldsymbol{\Xi}$ is a compartment solution of the ODE, $\boldsymbol{z}$ is a column vector, and $f_{j}$ indicates changes in the parameters. In this case, the result is a column vector of the size corresponding to the number of times, with the parenthesis specifying which compartment is involved and the differences in the parameters.

Thus, the cumulative deaths for a $\boldsymbol{(\Xi,}j\mathbf{)}$ compartment and parameter variation at time $t$ is:

|  | ${cm}_{t}\boldsymbol{(\Xi,}j\boldsymbol{)=}\sum_{i \leq t} \boldsymbol{mort}_{i}\boldsymbol{(\Xi,}j\boldsymbol{)} ,$ | $\boldsymbol{(} SEQ Ecuación \backslash* ARABIC 70\boldsymbol{)}$ |
| --- | --- | --- |

where $i$ denotes the $i$-th position of the vector.

The column vector of cumulative deaths up to time $t$ is:

|  | $\boldsymbol{cm(\Xi,}j\boldsymbol{)=}\left( \begin{matrix} {cm}_{1}\boldsymbol{(\Xi,}j\boldsymbol{)} \\ \begin{matrix} \vdots\\ \begin{matrix} {cm}_{t}\boldsymbol{(\Xi,}j\boldsymbol{)} \\ \vdots\\ {cm}_{t_{f}}\boldsymbol{(\Xi,}j\boldsymbol{)} \end{matrix} \end{matrix} \end{matrix} \right), t\in\left\{ 1,\ldots,t_{f} \right\}.$ | $\boldsymbol{(} SEQ Ecuación \backslash* ARABIC 71\boldsymbol{)}$ |
| --- | --- | --- |

Applying the round function to the above equation:

|  | $\boldsymbol{cd(\Xi,}j\boldsymbol{)}=\left\{ \begin{matrix} \left\lfloor\boldsymbol{cm(\Xi,}j\boldsymbol{)}\left. +0.5 \right\rfloor\right. & if & \forall t, {cm}_{t}\boldsymbol{(\Xi,}j\boldsymbol{)}\geq0 \\ & & \\ \boldsymbol{0} & if & \forall t, {cm}_{t}\boldsymbol{(\Xi,}j\boldsymbol{)}<0 \end{matrix}. \right.$ | $\boldsymbol{(} SEQ Ecuación \backslash* ARABIC 72\boldsymbol{)}$ |
| --- | --- | --- |

Cumulative deaths from COVID-19

For cumulative COVID-19 deaths the associated compartments are (see equation $(69)$):

| $\boldsymbol{mort(H,}1\boldsymbol{) =}$ | $\nu_{s}\cdot p_{ho2}\cdot p_{dho}\cdot\boldsymbol{d}_{\boldsymbol{2}\boldsymbol{h}}\boldsymbol{\odot dm\odot}\left( \boldsymbol{H}\cdot\boldsymbol{ifr} \right),$ | $\boldsymbol{(} SEQ Ecuación \backslash* ARABIC 73\boldsymbol{)}$ |
| --- | --- | --- |
| $\boldsymbol{mort(H,}2\boldsymbol{) =}$ | $\nu_{s}\cdot\left( 1 -p_{ho2} \right)\cdot p_{dh}\cdot\boldsymbol{dm\odot}\left( \boldsymbol{H}\cdot\boldsymbol{ifr} \right),$ |  |
| $\boldsymbol{mort(HC,}3\boldsymbol{) =}$ | $\nu_{sc}\cdot r_{dHC}\cdot p_{ho2}\cdot p_{dhco}\cdot\boldsymbol{dm\odot}\left( \boldsymbol{HC}\cdot\boldsymbol{ifr} \right),$ |  |
| $\boldsymbol{mort(HC,}4\boldsymbol{) =}$ | $\nu_{sc}\cdot r_{dHC}\cdot\left( 1 - p_{ho2} \right)\cdot p_{hc}\cdot\boldsymbol{dm\odot}\left( \boldsymbol{HC}\cdot\boldsymbol{ifr} \right),$ |  |
| $\boldsymbol{mort(ICU,}5\boldsymbol{) =}$ | $\nu_{icu} \cdot p_{ho2}\cdot p_{dicuo}\cdot\boldsymbol{d}_{\boldsymbol{2}\boldsymbol{h}}\boldsymbol{\odot dm\odot}\left( \boldsymbol{ICU}\cdot\boldsymbol{ifr} \right)\boldsymbol{,}$ |  |
| $\boldsymbol{mort(ICU,}6\boldsymbol{) =}$ | $\nu_{icu}\cdot\left( 1 - p_{ho2} \right)\cdot p_{dicu}\cdot\boldsymbol{dm\odot}\left( \boldsymbol{ICU}\cdot\boldsymbol{ifr} \right),$ |  |
| $\boldsymbol{mort(ICUC,}7\boldsymbol{) =}$ | $\nu_{icuc}\cdot p_{ho2}\cdot p_{dicuco}\cdot\boldsymbol{d}_{\boldsymbol{2}\boldsymbol{ch}}\boldsymbol{\odot dm\odot}\left( \boldsymbol{ICUC}\cdot\boldsymbol{ifr} \right),$ |  |
| $\boldsymbol{mort(ICUC,}8\boldsymbol{) =}$ | $\nu_{icuc}\cdot\left( 1 - p_{ho2} \right)\cdot p_{dicuc}\cdot\boldsymbol{dm\odot}\left( \boldsymbol{ICUC}\cdot\boldsymbol{ifr} \right),$ |  |
| $\boldsymbol{mort(Vent,}9\boldsymbol{) =}$ | $\nu_{v}\cdot p_{dv}\cdot\boldsymbol{dm\odot}\boldsymbol{d}_{\boldsymbol{vh}}\boldsymbol{\odot}\left( \boldsymbol{Vent}\cdot\boldsymbol{ifr} \right),$ |  |
| $\boldsymbol{mort(VentC,}10\boldsymbol{) =}$ | $\nu_{vc}\cdot p_{dvc}\cdot\boldsymbol{dm\odot}\boldsymbol{d}_{\boldsymbol{vch}}\boldsymbol{\odot}\left( \boldsymbol{VentC}\cdot\boldsymbol{ifr} \right),$ |  |
| $\boldsymbol{mort(ICUCV,}11\boldsymbol{) =}$ | $\nu_{vc}\cdot p_{dvc}\cdot\boldsymbol{dm\odot}\boldsymbol{d}_{\boldsymbol{vch}}\boldsymbol{\odot}\left( \boldsymbol{ICUCV}\cdot\boldsymbol{ifr} \right),$ |  |
| $\boldsymbol{mort(HCICU,}12\boldsymbol{) =}$ | $\nu_{sc}\cdot p_{ho2}\cdot p_{dicuhco}\cdot\boldsymbol{dm\odot}\left( \boldsymbol{HCICU}\cdot\boldsymbol{ifr} \right),$ |  |
| $\boldsymbol{mort(HCICU,}13\boldsymbol{) =}$ | $\nu_{sc}\cdot\left( 1 - p_{ho2} \right)\cdot p_{dicuhc}\cdot\boldsymbol{dm\odot}\left( \boldsymbol{HCICU}\cdot\boldsymbol{ifr} \right),$ |  |
| $\boldsymbol{mort(HCV,}14\boldsymbol{) =}$ | $\nu_{vc}\cdot p_{dvh}\cdot\boldsymbol{dm\odot}\left( \boldsymbol{HCV}\cdot\boldsymbol{ifr} \right)\boldsymbol{.}$ |  |

And applying equations $(70)$, $(71)$ and $(72)$ to the set of compartments $\Omega$and 14 variations in the parameters, we obtain:

| $\boldsymbol{d\_from\_covid=}$ | $\sum_{(\boldsymbol{\Xi}\in\Omega,j=1,\ldots,14)} \boldsymbol{cd(\Xi,}j\boldsymbol{)},$ $\Omega=\{\boldsymbol{H}, \boldsymbol{HC}, \boldsymbol{ICU}, \boldsymbol{ICUC}, \boldsymbol{Vent}, \boldsymbol{VentC}, \boldsymbol{ICUCV}, \boldsymbol{HCICU}, \boldsymbol{HCV\}}.$ | $\boldsymbol{(} SEQ Ecuación \backslash* ARABIC 74\boldsymbol{)}$ |
| --- | --- | --- |

Cumulative number of deaths from unexposed natural causes

Applying equations $(69)$, $(70)$, $(71)$ and $(72)$ we obtain the cumulative number of unexposed natural death, for $\boldsymbol{z} =\boldsymbol{\mu}$ and $f_{j}=1 \forall j, j\in\{1,...,5\}$:

| $\boldsymbol{mort}\left( \boldsymbol{\Xi}\boldsymbol{,j} \right)\boldsymbol{=}$ | $\boldsymbol{\Xi}\cdot\boldsymbol{\mu,}\boldsymbol{\Xi}\boldsymbol{\in}\Omega=\left\{ \boldsymbol{S},\boldsymbol{V},\boldsymbol{QS, QSR, SR} \right\},$ | $\boldsymbol{(} SEQ Ecuación \backslash* ARABIC 75\boldsymbol{)}$ |
| --- | --- | --- |
| $\boldsymbol{nd\_unexposed=}$ | $\sum_{(\boldsymbol{\Xi}\in\Omega,j=1, \ldots, 5)} \boldsymbol{cd(\Xi,}j\boldsymbol{)}, \Omega=\{\boldsymbol{S},\boldsymbol{V},\boldsymbol{QS, QSR, SR}\}.$ |  |

Cumulative number of exposed natural deaths

Finally, the cumulative number of exposed natural deaths is calculated using equations $(69)$, $(70)$, $(71)$ and $(72)$ where $\Omega=${**E**, **I**, **CL**, **X**, **R**, **ER**, **EV**, **EVR**, **QE**, **QI**, **QC**, **QR**, **QEV**, **QER**, **QEVR**, **QVR**, **H**, **HC**, **ICU**, **ICUC, ICUCV**, **Vent**, **VentC**, **HCICU**, **HCV**}.

| $\sum_{(\boldsymbol{\Xi}\in\Omega,j=1, \ldots, 25)} \boldsymbol{mort(\Xi,}j\boldsymbol{)}\boldsymbol{=}$ | $r_{ndI}\cdot\boldsymbol{E}\cdot\boldsymbol{\mu+}r_{ndI}\cdot\boldsymbol{I}\cdot\boldsymbol{\mu+}r_{ndCL}\cdot\boldsymbol{CL}\cdot\boldsymbol{\mu+}r_{ndCL}\cdot\boldsymbol{X}\cdot\boldsymbol{\mu}$  $\boldsymbol{+ R}\cdot\boldsymbol{\mu+}r_{ndI}\cdot\boldsymbol{ER}\cdot\boldsymbol{\mu+}r_{ndI}\cdot\boldsymbol{EV}\cdot\boldsymbol{\mu+}r_{ndI}\cdot\boldsymbol{EVR}\cdot\boldsymbol{\mu}$  $\boldsymbol{+}r_{ndI}\cdot\boldsymbol{QE}\cdot\boldsymbol{\mu+}r_{ndI}\cdot\boldsymbol{QI}\cdot\boldsymbol{\mu+}r_{ndCL}\cdot\boldsymbol{QC}\cdot\boldsymbol{\mu+QR}\cdot\boldsymbol{\mu}$  $\boldsymbol{+}r_{ndI}\cdot\boldsymbol{QEV}\cdot\boldsymbol{\mu+}r_{ndI}\cdot\boldsymbol{QER}\cdot\boldsymbol{\mu+}r_{ndI}\cdot\boldsymbol{QEVR}\cdot\boldsymbol{\mu}$  $\boldsymbol{+ QVR}\cdot\boldsymbol{\mu+H}\cdot\boldsymbol{\mu+}r_{dHC}\cdot\boldsymbol{HC}\cdot\boldsymbol{\mu+ICU}\cdot\boldsymbol{\mu}$  $\boldsymbol{+ ICUC}\cdot\boldsymbol{\mu+ICUCV}\cdot\boldsymbol{\mu+Vent}\cdot\boldsymbol{\mu+VentC}\cdot\boldsymbol{\mu}$  $\boldsymbol{+}r_{dHC}\cdot\boldsymbol{HCICU}\cdot\boldsymbol{\mu+}r_{dHC}\cdot\boldsymbol{HCV}\cdot\boldsymbol{\mu.}$ | $\boldsymbol{(} SEQ Ecuación \backslash* ARABIC 76\boldsymbol{)}$ |
| --- | --- | --- |

We can separate each vector of $\boldsymbol{mort(\Xi,}j\boldsymbol{)}$ to do the calculations and obtain:

| $\boldsymbol{nd\_exposed=}$ | $\sum_{\left( \boldsymbol{\Xi}\in\Omega,j=1,\ldots,25 \right)} \boldsymbol{cd}\left( \boldsymbol{\Xi,}j \right),$ $\Omega=\{\boldsymbol{E, I, CL, X, R, ER, EV, EVR, QE, QI, QC, QR,QEV, QER,}$  $\boldsymbol{QEVR, QVR, H, HC, ICU, ICUC, ICUCV, Vent, VentC, HCICU, HCV}\}.$ | $\boldsymbol{(} SEQ Ecuación \backslash* ARABIC 77\boldsymbol{)}$ |
| --- | --- | --- |

Cumulative deaths treated in hospital

Taking the information of $\boldsymbol{mort}(\boldsymbol{H},1)$ and $\boldsymbol{mort}(\boldsymbol{H},2)$ in equation $(73)$, and using equations $(70)$, $(71)$ and $(72)$, the cumulative number of deaths attended in the hospital is estimated as:

| $\boldsymbol{d\_th=}$ | $\sum_{j=1,2} \boldsymbol{cd}\left( \boldsymbol{H,}j \right).$ | $\boldsymbol{(} SEQ Ecuación \backslash* ARABIC 78\boldsymbol{)}$ |
| --- | --- | --- |

Cumulative deaths treated in the ICU

Taking the information from $\boldsymbol{mort}(\boldsymbol{ICU},5)$ and $\boldsymbol{mort}(\boldsymbol{ICU},6)$ in equation $(73)$, and using equations $(70)$, $(71)$ and $(72)$, the cumulative number of deaths treated in the ICU is estimated as:

| $\boldsymbol{d\_ticu=}$ | $\sum_{j=5,6} \boldsymbol{cd(ICU,}j\boldsymbol{)}.$ | $\boldsymbol{(} SEQ Ecuación \backslash* ARABIC 79\boldsymbol{)}$ |
| --- | --- | --- |

Cumulative ventilator-treated deaths

The number of ventilator-treated deaths is calculated by taking the information from equation $(73)$, $\boldsymbol{mort}\left( \boldsymbol{Vent},9 \right)$, and applying the equations $(70)$, $(71)$ and $(72)$:

| $\boldsymbol{d\_tv=}$ | $\boldsymbol{cd}\left( \boldsymbol{Vent,}9 \right)\boldsymbol{.}$ | $\boldsymbol{(} SEQ Ecuación \backslash* ARABIC 80\boldsymbol{)}$ |
| --- | --- | --- |

Cumulative untreated hospital deaths

The cumulative number of untreated deaths in the hospital is determined using equation $\left( 73 \right)$, changing the last three lines to:

| $\boldsymbol{mort(HCICU,}12\boldsymbol{) =}$ | $\nu_{sc}\cdot r_{dHC}\cdot p_{ho2}\cdot p_{dicuhco}\cdot\boldsymbol{(HCICU}\cdot\boldsymbol{ifr)}$, | $\boldsymbol{(} SEQ Ecuación \backslash* ARABIC 81\boldsymbol{)}$ |
| --- | --- | --- |
| $\boldsymbol{mort(HCICU,}13\boldsymbol{) =}$ | $\nu_{sc}\cdot r_{dHC}\cdot(1 - p_{ho2})\cdot p_{dicuhc}\cdot\boldsymbol{(HCICU}\cdot\boldsymbol{ifr)}$, |  |
| $\boldsymbol{mort(HCV,}14\boldsymbol{) =}$ | $\nu_{vc}\cdot r_{dHC}\cdot p_{dvh}\cdot\boldsymbol{(HCV}\cdot\boldsymbol{ifr)}$, |  |
| $\boldsymbol{d\_uth=}$ | $\sum_{(\boldsymbol{\Xi}\in\Omega, j=\{3, 4, 12, 13, 14\})} \boldsymbol{cd(\Xi,}j\boldsymbol{)}, \Omega= \left\{ \boldsymbol{HC,HCICU,HCV} \right\}.$ |  |

Cumulative untreated ICU deaths

The number of untreated deaths in ICU is calculated by taking the information from equation $(73)$ and applying equations $(70)$, $(71)$ and $(72)$:

| $\boldsymbol{d\_uticu =}$ | $\sum_{j=7,8} \boldsymbol{cd}\left( \boldsymbol{ICUC,}j \right).$ | $\boldsymbol{(} SEQ Ecuación \backslash* ARABIC 82\boldsymbol{)}$ |
| --- | --- | --- |

Death without ventilation treatment

Using the equation $(73)$:

| $\boldsymbol{d\_utv =}$ | $\boldsymbol{cd}\left( \boldsymbol{VentC,}10 \right)\boldsymbol{+cd}\left( \boldsymbol{ICUCV,}11 \right)\boldsymbol{.}$ | $\boldsymbol{(} SEQ Ecuación \backslash* ARABIC 83\boldsymbol{)}$ |
| --- | --- | --- |

Deaths from different causes attributable to COVID-19

Death from causes related to equations $(78)$, $(79)$, $(80)$, $(81)$, $(82)$ and $(83)$:

| $\boldsymbol{ad =d\_th+ d\_ticu+ d\_tv+ d\_uth+d\_uticu + d\_utv.}$ | $\boldsymbol{(} SEQ Ecuación \backslash* ARABIC 84\boldsymbol{)}$ |
| --- | --- |

Total accumulated deaths

Using equations $(75)$, $(77)$ and $(84)$:

| $\boldsymbol{total\_deaths =ad+ nd\_unexposed+ nd\_exposed.}$ | $\boldsymbol{(} SEQ Ecuación \backslash* ARABIC 85\boldsymbol{)}$ |
| --- | --- |

Indicator of the number of cases that die from COVID-19 daily

Remember that $t_{f}$ is the final time of the study, and this provides an estimation of the mortality lag.

| $\boldsymbol{ml}(day)=\left\{ \begin{matrix} \boldsymbol{CM}_{day,\cdot}\boldsymbol{⊘}\boldsymbol{C}_{day,\cdot} & if & t_{f}\geq day \\ & & \\ 0 & if & \boldsymbol{CM}_{day,\cdot}\boldsymbol{⊘}\boldsymbol{C}_{day,\cdot}\to+\infty\end{matrix} \right.,$ | $\boldsymbol{(} SEQ Ecuación \backslash* ARABIC 86\boldsymbol{)}$ |
| --- | --- |

where $\boldsymbol{\Xi}_{day,\cdot}$ denotes the time at $day$ for all the age groups in the compartment $\boldsymbol{\Xi}$, therefore we obtain a vector for all the age groups on a specific day.

**G. Calibration of parameters in the model**

Parameter calibration in the ODE is necessary when incorporating observed data into the CoMo model, as the initial values provided by the literature or real-world evidence must be adjusted. For this purpose, several constrained optimisation methods were implemented. The objective was to minimise the mean absolute error (MAE) between the daily cases $(66)$, cumulative deaths $(67)$ (with $\gamma=1,\Omega=\{\boldsymbol{CM}\}$), symptomatic, ICU and hospitalised observed cases, and those predicted by the model (all of them with a min-max transformation due to the different scales), in some cases considering a smoothing on the cases and symptomatic by a normal kernel.

For the calibration, we considered the entire sample from the first reported case on 27 February 2020 until 4 August 2022. A brief description of the methods used can be found in Table 1, noting that derivative-free methods have a higher calibration (lower error) than gradient-based algorithms. Here it is also important to mention that the time required to choose the method was considered, as some methods would not reach their best optimum due to the iterations performed. The chosen method had to reach the minimum value among all of them with the same number of iterations and with a computational time trade-off.

The final method chosen was *gosolnp* with *solnp*, the former generates uniform values between the lower and upper bound to find an optimal starting point and then performs the minimisation with the *solnp* algorithm based on the augmented Lagrange method. In the first stage, calibration was performed on all parameters using 5,000 iterations achieving an error value of approximately 2.6708. With these new values, the method was reset using only the *solnp* algorithm.

| **Library** | **Function** | **Method** | **Description** | **Final MAE, approximated time (hours)** |
| --- | --- | --- | --- | --- |
| Rsolnp | gosolnp | solnp | Random initialization and multiple restarts of the solnp solver, which is a nonlinear optimisation using augmented Lagrange method (Ye, 1988). | 2.67 (53.79) |
| Jaya | jaya | jaya | Gradient-free optimisation algorithm. A population-based method which repeatedly modifies a population of individual solutions (Rao, 2016). | 2.70 (92.95) |
| nloptr | bobyqa | bobyqa | Performs derivative-free bound-constrained optimisation using an iteratively constructed quadratic approximation for the objective function (Powell, 2009). | 2.74 (28.44) |
| Rvmmin | Rvmmin | rvmmin | The algorithm is based on Nash (2018), an approximation to the inverse Hessian is used to generate a search direction and a simple backtracking line search is used until an acceptable point is found. | 2.77 (150.60) |
| Rcgmin | Rcgmin | rcgmin | Implementation of a nonlinear conjugate gradient algorithm with  the Dai & Yuan (2001) update and restart. | 2.85 (308.66) |
| calibrar | calibrate | AHR-ES | Performs the optimisation of a function using evolutionary strategies (Oliveros-Ramos & Shin, 2016). | 3.28 (28.28) |
| nloptr | cobyla | cobyla | Algorithm for derivative-free optimisation with nonlinear inequality and equality constraints (Powell, 1994). | 3.38 (28.37) |
| hydroPSO | hydroPSO | spso2011 | State-of-the-art version of the particle swarm optimisation. At each iteration particles are attracted to its own best-known ‘personal’ and to the best-known position in its ‘local’ neighbourhood (Clerc, 2012). | 3.39 (95.10) |
| OOR | StoSOO | stosoo | Implementation of optimistic optimisation methods for global optimisation of deterministic or stochastic functions (Valko et al., 2013). | 5.08 (28.35) |
| FME | modFit | marq | Modification of the Levenberg-Marquardt algorithm for solving nonlinear least-squares problems (Moré, 1978). | Error in finding the final value  (2.45) |
|  |  | pseudo | Pseudo-random search optimisation algorithm of Price (1977), a random-based fitting technique. | Error in finding the final value  (31.74) |

**Table 1.** Description of the optimisation methods considered.

*Note*. The methods were computed with 5,000 iterations each and considering the smoothing version in the cases.

Sensitivity studies were performed for each parameter, confirming differences in cumulative cases and deaths compared to the base case. For some parameters, the cumulative cases and deaths curves were not significantly affected and were therefore considered fixed. Initial non-sensitive parameters were taken from established literature sources or clinical experience, and initial values for sensitive parameters were set according to the previously found values. The parameters that were calibrated are shown in Table 2. After defining the parameters, the process involved optimisation within observed values across six defined windows of time. Six k-folds were defined according to monthly reported data from February to July 2022, to continue testing predictability outside the window against real data, ensuring good forecast performance. The final MAE value was 0.1592.

| Parameter | Code name | Description |
| --- | --- | --- |
| $p$ | p | Probability of infection given contact. |
| $r$ | report | Percentage of all asymptomatic infections that are reported. |
| $r_{c}$ | reportc | Percentage of all symptomatic infections that are reported. |
| $r_{g}$ | reporth_g | Percentage of denied hospitalisations that are reported. |
| $r_{hICU}$ | reporth_ICU | Percentage of severe hospitalisations that are appropriately treated. |
| $r_{r}$ | report_r | Percentage of all asymptomatic infections in previously infected people that are reported. |
| $r_{cr}$ | report_cr | Percentage of all symptomatic infections in previously infected people that are reported. |
| $r_{ndI}$ | report_natdeathI | Percentage of all people dying outside the hospital with asymptomatic infections reported as covid-deaths. |
| $r_{dHC}$ | report_death_HC | Percentage of all people dying outside the hospital with severe infections reported as covid-deaths. |
|  | | |
| Virus parameters | | |
| $\rho$ | rho | Relative infectiousness of incubation phase. |
| $\frac{1}{\gamma}$ | gamma | Average incubation period (1 to 7 days). |
| $\frac{1}{\nu}$ | nui | Average duration of symptomatic infection period (1 to 7 days). |
| $\phi$ | phi | Month of peak infectivity of the virus (1, 2, …, 12). |
| $\zeta$ | amp | Annual variation in infectivity of the virus. |
| $\frac{1}{\omega}$ | omega | Average duration of immunity (0.5 to 150 years). |
| $p_{c}$ | pclin | Probability upon infection of developing clinical symptoms. |
| $p_{icu}$ | prob_icu | Probability upon hospitalisation of requiring Intensive Care Units (ICU) admission. |
| $p_{v}$ | prob_vent | Probability upon admission to the ICU of requiring a ventilator. |
| $p_{ho2}$ | propo2 | Proportion of hospitalised patients needing O2. |
|  | | |
| Hospitalisation parameters | | |
| $ihr_{s}$ | ihr_scaling | Scaling factor for infection hospitalisation rate (0.1 to 5) |
| $p_{dho}$ | pdeath_ho | Probability of dying when hospitalised if req. O2. |
| $p_{dhc}$ | pdeath_hc | Probability of dying when denied hospitalisation (not req. O2). |
| $p_{dhco}$ | pdeath_hco | Probability of dying when denied hospitalisation if req. O2. |
| $p_{dicuo}$ | pdeath_icuo | Probability of dying when admitted to ICU if req. O2. |
| $p_{dicuco}$ | pdeath_icuco | Probability of dying when admission to ICU denied if req. O2. |
| $p_{dv}$ | pdeath_vent | Probability of dying when ventilated. |
| $p_{dvc}$ | pdeath_ventc | Probability of dying when ventilator denied. |
| $\frac{1}{\nu_{s}}$ | nus | Duration of hospitalised infection (1 to 30 days). |
| $\frac{1}{\nu_{icu}}$ | nu_icu | Duration of ICU infection (1 to 30 days). |
| $\frac{1}{\nu_{v}}$ | nu_vent | Duration of ventilated infection (1 to 30 days). |
|  | | |
| Vaccination parameters | | |
| $p_{cr}$ | pclin_r | Probability upon infection of developing clinical symptoms if previously infected. |
| $\sigma_{R}$ | sigmaR | Probability of infection of people that have recovered from a previous infection. |
| $\sigma_{ER}$ | sigmaER | Probability of requiring hospitalisation if previously infected. |
| $vac_{c}$ | vac_campaign | Vaccination - Time to reach target coverage (1 to 52 weeks). |
| $\frac{1}{vac_{dr}}$ | vac_dur_r | Vaccination - Duration of efficacious period if previously infected (years). |
|  | | |
| Intervention parameters | | |
| $self_{eff}$ | selfis_eff | Self-isolation if symptomatic (adherence). I.e., estimated reduction in contact due to self-isolation if symptomatic. |
| $d_{eff}$ | dist_eff | Social distancing (adherence). That is, reduction of contacts in the community between those who adhere to social distancing. |
| $m_{eff}$ | mask_eff | Efficacy of the use of masks (0-35%).  Estimated reduction of contact due to mask use. |
| $w_{eff}$ | work_eff | Work at home (efficiency). I.e., reduction of contacts at work among those who adhere to work-from-home policies. |
| $w_{2h}$ | w2h | Home contacts inflation due to working from home. |
| $d_{2c}$ | dexo2c | Relative risk of dying if needing but not receiving O2 and taking dexamethasone*.* |
| $d_{vc}$ | dexvc | Relative risk of dying if needing but not receiving ventilation and taking dexamethasone. |
|  | | |
| **Interventions** | | |
| $\boldsymbol{1}_{\boldsymbol{scc}}(t)$ | schoolclose2 | Age-indicator school closure apply for a specific time. |
| $\sigma_{Rmod}(t)$ | cmod_vector | The function relates the time and the value corresponding to breakthrough infection probability. |
| $dm(t)$ | dmod_vector | The function relates the time and the value corresponding to the lethality of the variant. |
| $pm(t)$ | pmod_vector | The function relates the time and the value corresponding to the transmissibility of the variant. |
| $m_{cov}(t)$ | mask_cov | The function relates the time and the value corresponding to the adherence to mask wearing. |
| $d_{cov}(t)$ | dist_cov | The function relates the time and the value corresponding to the adherence to social distancing in community level. |
| $w_{cov}(t)$ | work_cov | The function relates the time and the value corresponding to the adherence to work from home policies. |

**Table 2.** Parameters calibrated.

References

Aguas, R., White, L., Hupert, N., Shretta, R., Pan-Ngum, W., Celhay, O., Moldokmatova, A., Arifi, F., Mirzazadeh, A., Sharifi, H., Adib, K., Sahak, M. N., Franco, C., Coutinho, R., Ariana, P., Hancock, P., Kraenkel, R. A., Saralamba, S., Luangasanatip, N., … Obiesie, S. (2020). Modelling the COVID-19 pandemic in context: an international participatory approach. *BMJ Global Health*, *5*(12), e003126. https://doi.org/10.1136/BMJGH-2020-003126

Alcaldía de Bogotá D.C. (2021a). *Ellos fueron los primeros vacunados contra el COVID-19 en Bogotá*.

Alcaldía de Bogotá D.C. (2021b). *En Bogotá se han aplicado 9.643.773 dosis de vacunas contra COVID-19*.

Alfego, D., Sullivan, A., Poirier, B., Williams, J., Adcock, D., & Letovsky, S. (2021). A population-based analysis of the longevity of SARS-CoV-2 antibody seropositivity in the United States. *EClinicalMedicine*, *36*, 100902. https://doi.org/10.1016/j.eclinm.2021.100902

Australian Bureau of Statistics. (2022). *Household impacts of COVID-19 survey, April 2022*.

Ayoubkhani, D., Bermingham, C., Pouwels, K. B., Glickman, M., Nafilyan, V., Zaccardi, F., Khunti, K., Alwan, N. A., & Walker, A. S. (2022). Trajectory of long covid symptoms after covid-19 vaccination: community based cohort study. *BMJ*, *377*, e069676. https://doi.org/10.1136/BMJ-2021-069676

BIO-TEST IPS S.A.S. (2020). *Informe de verificación de la técnica SARS-CoV-2 RT-PCR de diagnóstico cualitativo basado en la técnica ONE-STEP RT multiplex en tiempo real*.

Borges, M., Souto, L., Poloni, S., Maria Bagattini, A., Franco, C., Quarti Machado da Rosa, M., Mendes Simon, L., Alves Camey, S., de Souza Kuchenbecker, R., Inácio Prado, P., Alexandre Felizola Diniz Filho, J., André Kraenkel, R., Mendes Coutinho, R., Maria Toscano, C., & Covid-, O. (2021). Modeling the impact of school reopening and contact tracing strategies on COVID-19 dynamics in different epidemiologic settings in Brazil. *MedRxiv*, 2021.10.22.21264706. https://doi.org/10.1101/2021.10.22.21264706

Celhay, O. (2021). *covidage_v19.1.3.R*.

Center for Disease Control and Prevention. (2021, October). *Science brief: SARS-CoV-2 infection-induced and vaccine-induced immunity*.

Centers for Disease Control and Prevention. (2020). *Show me the science - Why wash your hands?*

Centers for Disease Control and Prevention. (2022). *COVID data tracker*.

Clerc, M. (2012). Beyond Standard Particle Swarm Optimisation. In *Innovations and Developments of Swarm Intelligence Applications* (pp. 1–19). IGI Global. https://doi.org/10.4018/978-1-4666-1592-2.ch001

Clínica Universitaria Medicina Integral S.A.S. (2020). Informe verificación de desempeño ensayo molecular para detección de ARN del virus SARS-CoV-2 en el sistema MINI8 plus con utilización de DIRECTDETECT SARS-CoV-2 DETECTION KIT. In *Clínica universitaria medicina integral*.

CoMo Consortium. (2022). *CoMo Consortium*.

Czeisler, M., Marynak, K., Clarke, K., Salah, Z., Shakya, I., Thierry, J., Ali, N., McMillan, H., Wiley, J., Weaver, M., Czeisler, C., Rajaratnam, S., & Howard, M. (2022, September). *Delay or avoidance of medical care because of COVID-19–related concerns — United States, June 2020*. MMWR. Morbidity and Mortality Weekly Report; Centers for Disease Control MMWR Office. https://doi.org/10.15585/MMWR.MM6936A4

Dai, Y., & Yuan, Y. (2001). An Efficient Hybrid Conjugate Gradient Method for Unconstrained Optimization. *Annals of Operations Research*, *103*, 33–47. https://doi.org/10.1023/A:1012930416777

Datos Abiertos Bogotá. (2022). *Ocupación Unidades de Cuidado Intensivo (UCI) COVID-19 en Bogotá*.

Departamento Administrativo Nacional de Estadística. (2020a). *Geovisor Estadísticas de Migración Internacional*.

Departamento Administrativo Nacional de Estadística. (2020b). *Proyecciones de viviendas y hogares*.

Departamento Administrativo Nacional de Estadística. (2021). *Proyecciones de población Bogotá*.

Departamento Administrativo Nacional de Estadística. (2022a, January). *Defunciones no fetales 2022*.

Departamento Administrativo Nacional de Estadística. (2022b, February). *Nacimientos 2020*.

Departamento Administrativo Nacional de Estadística. (2022c, March). *Defunciones no fetales 2020*.

Departamento Administrativo Nacional de Estadística. (2022d, June). *Defunciones no fetales 2021*.

Departamento Administrativo Nacional de Estadística. (2022e, June). *Nacimientos 2021*.

Departamento Administrativo Nacional de Estadística. (2022f, June). *Nacimientos 2022*.

Diarra, M., Kebir, A., Talla, C., Barry, A., Faye, J., Louati, D., Opatowski, L., Diop, M., REPAIR consortium, White, L. J., Loucoubar, C., & Miled, S. Ben. (2022). Non-pharmaceutical interventions and COVID-19 vaccination strategies in Senegal: a modelling study. *BMJ Global Health*, *7*(2), 1–9. https://doi.org/10.1136/bmjgh-2021-007236

El Tiempo. (2020). *1 de cada 4 muertes por covid en Colombia se daría fuera de hospitales*.

Endo, A., Abbott, S., Kucharski, A., & Funk, S. (2020). Estimating the overdispersion in COVID-19 transmission using outbreak sizes outside China. *Wellcome Open Research*, *5*, 1–18. https://doi.org/10.12688/WELLCOMEOPENRES.15842.3

Franco, C. (2021). *Mathematical modelling of epidemiological systems: from data and equations to public health implications*.

Franco, C., Ferreira, L., Sudbrack, V., Borges, M., Poloni, S., Prado, P., White, L. J., Águas, R., Kraenkel, R., & Coutinho, R. (2022). Percolation across households in mechanistic models of non-pharmaceutical interventions in SARS-CoV-2 disease dynamics. *Epidemics*, *39*. https://doi.org/10.1016/J.EPIDEM.2022.100551

Gao, B. (2021). *comoOdeCpp V19.1.2*.

Gardner, B., & Kilpatrick, A. (2021). *Estimates of reduced vaccine effectiveness against hospitalization, infection, transmission and symptomatic disease of a new SARS-CoV-2 variant, Omicron (B.1.1.529), using neutralizing antibody titers*. https://doi.org/10.1101/2021.12.10.21267594

Gobierno Nacional de Colombia. (2020a). *Datos importantes sobre la cuarentena*.

Gobierno Nacional de Colombia. (2020b). *Medidas tomadas para el adulto mayor*.

González, C., Calleja, N., Carreño, J., Gómez, M. E., & Sánchez, C. (2021). Adherencia a las medidas preventivas del COVID-19 en la población mexicana durante la segunda fase de la pandemia. *FORHUM International Journal of Social Sciences and Humanities*, *5*, 129–142. https://doi.org/10.35766/J.FORHUM2021.03.05.8

Gutierrez, S., Salazar, R., Álvarez, L., Agudelo, C., Gonzalez, A., Cardenas, M., & Del Valle, H. (2021). *Detección de SARS-CoV-2 por RT-QPCR en muestras de saliva*.

Guzmán, Y., Vecino-Ortiz, A., Guzman-Tordecilla, N., Peñaloza-Quintero, R., Fernández-Niño, J., Rojas-Botero, M., Ruiz Gomez, F., Sullivan, S., & Trujillo, A. (2022). Cost-effectiveness of the COVID-19 test, trace and isolate program in Colombia. *The Lancet Regional Health - Americas*, *6*. https://doi.org/10.1016/J.LANA.2021.100109

Hall, V., Foulkes, S., Insalata, F., Kirwan, P., Saei, A., Atti, A., Wellington, E., Khawam, J., Munro, K., Cole, M., Tranquillini, C., Taylor-Kerr, A., Hettiarachchi, N., Calbraith, D., Sajedi, N., Milligan, I., Themistocleous, Y., Corrigan, D., Cromey, L., … Hopkins, S. (2022). Protection against SARS-CoV-2 after COVID-19 Vaccination and Previous Infection. *New England Journal of Medicine*, *386*(13), 1207–1220. https://doi.org/10.1056/NEJMOA2118691/SUPPL_FILE/NEJMOA2118691_DISCLOSURES.PDF

He, X., Lau, E. H. Y., Wu, P., Deng, X., Wang, J., Hao, X., Lau, Y. C., Wong, J. Y., Guan, Y., Tan, X., Mo, X., Chen, Y., Liao, B., Chen, W., Hu, F., Zhang, Q., Zhong, M., Wu, Y., Zhao, L., … Leung, G. M. (2020). Temporal dynamics in viral shedding and transmissibility of COVID-19. *Nature Medicine 2020 26:5*, *26*(5), 672–675. https://doi.org/10.1038/s41591-020-0869-5

Higdon, M. M., Baidya, A., Walter, K. K., Patel, M. K., Issa, H., Espié, E., Feikin, D. R., & Knoll, M. D. (2022). Duration of effectiveness of vaccination against COVID-19 caused by the omicron variant. *The Lancet Infectious Diseases*, *22*(8), 1114–1116. https://doi.org/10.1016/s1473-3099(22)00409-1

Hyman, J. M. (2006). Accurate monotonicity preserving cubic interpolation. *SIAM Journal on Scientific and Statistical Computing*, *4*(4), 645–654. https://doi.org/10.1137/0904045

Instituto Nacional de Salud. (2020a).  *Informe de detección de SARS-CoV-2 en saliva mediante el uso de proteinasa K y RT-qPCR*.

Instituto Nacional de Salud. (2020b). *Informe de verificación del estuche IAMP covid-19 detection kit (ATILA BIOSYSTEM) para la detección de SARS-CoV-2 mediante PCR isotérmica*.

Instituto Nacional de Salud. (2020c). *Casos positivos de COVID-19 en Colombia*.

Instituto Nacional de Salud. (2022a). *Antígeno procesadas de COVID-19 en Colombia (Departamental) | Datos Abiertos Colombia*.

Instituto Nacional de Salud. (2022b). *Boletines casos COVID-19 Colombia*.

Instituto Nacional de Salud. (2022c). *Pruebas PCR procesadas de COVID-19 en Colombia (Departamental) | Datos Abiertos Colombia*.

Khalili, M., Karamouzian, M., Nasiri, N., Javadi, S., Mirzazadeh, A., & Sharifi, H. (2020). Epidemiological characteristics of COVID-19: a systematic review and meta-analysis. *Epidemiology and Infection*, *148*. https://doi.org/10.1017/S0950268820001430

Laajaj, R., De Los Rios, C., Sarmiento-Barbieri, I., Aristizabal, D., Behrentz, E., Bernal, R., Buitrago, G., Cucunubá, Z., de la Hoz, F., Gaviria, A., Hernández, L. J., León, L., Moyano, D., Osorio, E., Varela, A. R., Restrepo, S., Rodriguez, R., Schady, N., Vives, M., & Webb, D. (2021). COVID-19 spread, detection, and dynamics in Bogota, Colombia. *Nature Communications 2021 12:1*, *12*(1), 1–8. https://doi.org/10.1038/s41467-021-25038-z

Laboratorio Clínico Alife Health. (2020). *Estudio para la evaluación de las especificaciones de desempeño de los reactivos de diagnóstico in vitro para la determinación cualitativa de SARS-CoV-2 para pruebas moleculares de PCR en tiempo real (RT QPCR) con el reactivo FTD^TM^ SARS-CoV-2 empleado en e*.

Laboratorio de salud pública departamental del Valle. (2020). *Informe verificación de desempeño ensayo molecular para detección de ARN del virus SARS-CoV-2 en el sistema BD MAX*.

Larsen, J., Martin, M., Martin, J., Hicks, J., & Kuhn, P. (2021). Modeling the onset of symptoms of COVID-19: Effects of SARS-CoV-2 variant. *PLOS Computational Biology*, *17*(12), e1009629. https://doi.org/10.1371/JOURNAL.PCBI.1009629

Lauer, S., Grantz, K., Bi, Q., Jones, F., Zheng, Q., Meredith, H., Azman, A., Reich, N., & Lessler, J. (2020). The incubation period of coronavirus disease 2019 (COVID-19) from publicly reported confirmed cases: estimation and application. *Annals of Internal Medicine*, *172*(9), 577–582. https://doi.org/10.7326/M20-0504

Lei, H., Xu, X., Xiao, S., Wu, X., & Shu, Y. (2020). Household transmission of COVID-19-a systematic review and meta-analysis. *Journal of Infection*, *81*(6), 979–997. https://doi.org/10.1016/j.jinf.2020.08.033

Lindsley, W. G., Blachere, F. M., Law, B. F., Beezhold, D. H., & Noti, J. D. (2021). Efficacy of face masks, neck gaiters and face shields for reducing the expulsion of simulated cough-generated aerosols. *Aerosol Science and Technology*, *55*(4), 449–457. https://doi.org/10.1080/02786826.2020.1862409/SUPPL_FILE/UAST_A_1862409_SM3229.PDF

Ministerio de Salud y Protección Social. (2021). *Efectividad de las vacunas contra el COVID-19 en Colombia*.

Moldokmatova, A., Estebesova, A., Dooronbekova, A., Zhumalieva, C., Mukambetov, A., Abdyldaev, T., Kubatova, A., Ibragimov, S., Usenbaev, N., Kutmanova, A., & White, L. J. (2020). *Mathematical modelling projections versus the actual course of the COVID-19 epidemic following the nationwide lockdown in Kyrgyzstan*.

Moré, J. (1978). *The Levenberg-Marquardt algorithm: Implementation and theory* (pp. 105–116). https://doi.org/10.1007/BFb0067700

Nash, J. (2018). *Compact Numerical Methods for Computers*. Routledge. https://doi.org/10.1201/9781315139784

Nasreen, S., Chung, H., He, S., Brown, K. A., Gubbay, J. B., Buchan, S. A., Fell, D. B., Austin, P. C., Schwartz, K. L., Sundaram, M. E., Calzavara, A., Chen, B., Tadrous, M., Wilson, K., Wilson, S. E., & Kwong, J. C. (2022). Effectiveness of COVID-19 vaccines against symptomatic SARS-CoV-2 infection and severe outcomes with variants of concern in Ontario. *Nature Microbiology 2022 7:3*, *7*(3), 379–385. https://doi.org/10.1038/s41564-021-01053-0

North, C. M., Barczak, A., Goldstein, R., Healy, B., Finkelstein, D., Ding, D., Kim, A., Boucau, J., Shaw, B., Gilbert, R., Vyas, T., Reynolds, Z., Siddle, K., Macinnis, B., Regan, J., Flynn, J., Choudhary, M., Vyas, J., Laskowski, K., … Sacks, C. (2022). Determining the incidence of asymptomatic SARS-CoV-2 among early recipients of COVID-19 vaccines (DISCOVER-COVID-19): a prospective cohort study of healthcare workers before, during and after vaccination. *Clinical Infectious Diseases*, *74*(7), 1275–1278. https://doi.org/10.1093/CID/CIAB643

Oliveros-Ramos, R., & Shin, Y.-J. (2016). Calibrar: an R package for fitting complex ecological models. *ArXiv Preprint.* https://doi.org/10.48550/arXiv.1603.03141

Pormohammad, A., Zarei, M., Ghorbani, S., Mohammadi, M., Neshin, S., Khatami, A., Turner, D., Djalalinia, S., Mousavi, S., Mardani-Fard, H., Kasaeian, A., & Turner, R. (2022). Effectiveness of covid-19 vaccines against delta (B.1.617.2) variant: a systematic review and meta-analysis of clinical studies. *Vaccines*, *10*(1), 23. https://doi.org/10.3390/VACCINES10010023/S1

Powell, M. (1994). A Direct Search Optimization Method That Models the Objective and Constraint Functions by Linear Interpolation. In *Advances in Optimization and Numerical Analysis* (pp. 51–67). Springer Netherlands. https://doi.org/10.1007/978-94-015-8330-5_4

Powell, M. (2009). The BOBYQA algorithm for bound constrained optimization without derivatives. *Cambridge NA Report NA2009/06*.

Prem, K., Cook, A. R., & Jit, M. (2017). Projecting social contact matrices in 152 countries using contact surveys and demographic data. *PLOS Computational Biology*, *13*(9), e1005697. https://doi.org/10.1371/JOURNAL.PCBI.1005697

Prem, K., Van, K., Klepac, P., Eggo, R., Davies, N., Cook, A., & Jit, M. (2021). Projecting contact matrices in 177 geographical regions: an update and comparison with empirical data for the COVID-19 era. *PLoS Computational Biology*, *17*(7), 1DUMMUY. https://doi.org/10.1371/JOURNAL.PCBI.1009098

Price, W. (1977). A controlled random search procedure for global optimisation. *The Computer Journal*, *20*(4), 367–370. https://doi.org/10.1093/comjnl/20.4.367

Rao, R. (2016). Jaya: A simple and new optimization algorithm for solving constrained and unconstrained optimization problems. *International Journal of Industrial Engineering Computations*, 19–34. https://doi.org/10.5267/j.ijiec.2015.8.004

Raslan, W. E. (2021). Fractional mathematical modeling for epidemic prediction of COVID-19 in Egypt. *Ain Shams Engineering Journal*, *12*(3), 3057–3062. https://doi.org/10.1016/J.ASEJ.2020.10.027

RECOVERY Collaborative Group. (2021). Dexamethasone in hospitalized patients with COVID-19. *N Engl J Med*, *8*, 693–704. https://doi.org/10.1056/NEJMoa2021436

Ren, X., Zhou, J., Guo, J., Hao, C., Zheng, M., Zhang, R., Huang, Q., Yao, X., Li, R., & Jin, Y. (2022). Reinfection in patients with COVID-19: a systematic review. *Global Health Research and Policy*, *7*(1), 1–20. https://doi.org/10.1186/S41256-022-00245-3/FIGURES/2

Reza, N., Ali, I., Setyo, D., Sutanto, H., Pudy, T., Dyah, C., & Mayjen, J. (2022). Effectiveness of Covid-19 vaccines against SARS-CoV-2 Omicron variant (B.1.1.529): a systematic review with meta-analysis and meta-regression. *MedRxiv*, 2022.04.29.22274454. https://doi.org/10.1101/2022.04.29.22274454

Rodriguez, D., Pinzón, Á., Rubio, C., Pinilla, D., Niño, M., Díaz, M., Molano-González, N., Ceballos, J., Arroyo, A., & Ruiz, Á. (2022). Clinical characteristics and mortality associated with COVID-19 at high altitude: a cohort of 5161 patients in Bogotá, Colombia. *International Journal of Emergency Medicine*, *15*(1). https://doi.org/10.1186/S12245-022-00426-4

SaluData. (2022a). *Casos confirmados de COVID-19*.

SaluData. (2022b). *Ocupacion-Hospitalizacion-COVID-19*.

SaluData. (2022c). *Porcentaje de ocupación de los servicios de hospitalización general, unidad de cuidado intermedio y unidad de cuidado intensivo en Bogotá D.C.*

Sheehan, M., Reddy, A., & Rothberg, M. (2021). *Reinfection rates among patients who previously tested positive for COVID-19: a retrospective cohort study*.

Smith, L. E., Potts, H. W. W., Amlôt, R., Fear, N. T., Michie, S., & Rubin, G. J. (2021). *Adherence to the test, trace, and isolate system in the UK: results from 37 nationally representative surveys*. https://doi.org/10.1136/bmj.n608

Tran, C., Bosetti, P., Paireau, J., Crépey, P., Salje, H., Lefrancq, N., Fontanet, A., Benamouzig, D., Boëlle, P. Y., Desenclos, J. C., Opatowski, L., & Cauchemez, S. (2021). SARS-CoV-2 transmission across age groups in France and implications for control. *Nature Communications 2021 12:1*, *12*(1), 1–12. https://doi.org/10.1038/s41467-021-27163-1

Universidad el Bosque. (2020). *Validación (verificación secundaria) KIT careGENE^TM^ N-CoV RT-PCR*. https://doi.org/10.5740/jaoacint.18-0320

Valko, M., Carpentier, A., & Munos, R. (2013). Stochastic simultaneous optimistic optimization. *International Conference on Machine Learning*, 19–27.

World Health Organization. (2020, July). *Transmission of SARS-CoV-2: implications for infection prevention precautions*.

Yanes-Lane, M., Winters, N., Fregonese, F., Bastos, M., Perlman-Arrow, S., Campbell, J. R., & Menzies, D. (2020). Proportion of asymptomatic infection among COVID-19 positive persons and their transmission potential: a systematic review and meta-analysis. *PLoS ONE*, *15*(11 November). https://doi.org/10.1371/JOURNAL.PONE.0241536

Ye, Y. (1988). *Interior algorithms for linear, quadratic, and linearly constrained convex programming* [Stanford University]. https://doi.org/10.5555/914114

Zalat, M., & Bolbol, S. (2022). Telework benefits and associated health problems during the long COVID-19 era. *Work (Reading, Mass.)*, *71*(2), 371–378. https://doi.org/10.3233/WOR-210691
