# Supplementary material for: Predictive modelling of the effectiveness of vaccines against COVID-19 in Bogotá: Methodological innovation involving different variants and computational optimisation efficiency
Source: Heliyon. 2024 Oct 23;10(21):e39725. doi: 10.1016/j.heliyon.2024.e39725 (PMC11570482; doi:10.1016/j.heliyon.2024.e39725)
Supplement: Multimedia component 3 [file mmc3.docx]

Supplement 3. Simulation of alternative scenarios

**Scenario 1: Low efficacy (and effectiveness) of the vaccine, relaxation of non-pharmacological measures**

**Scenario 1A**

|  | (A)  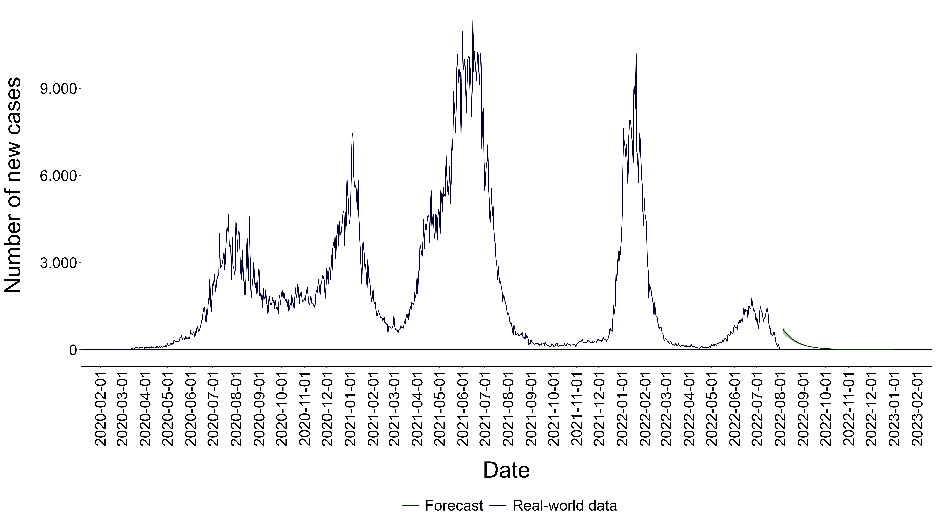 | (B)  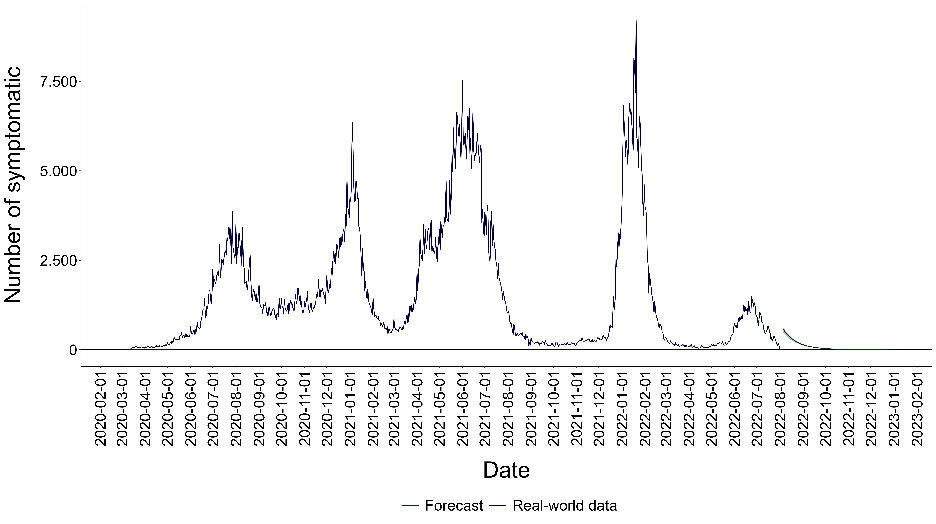 |
| --- | --- | --- |
|  | (C)  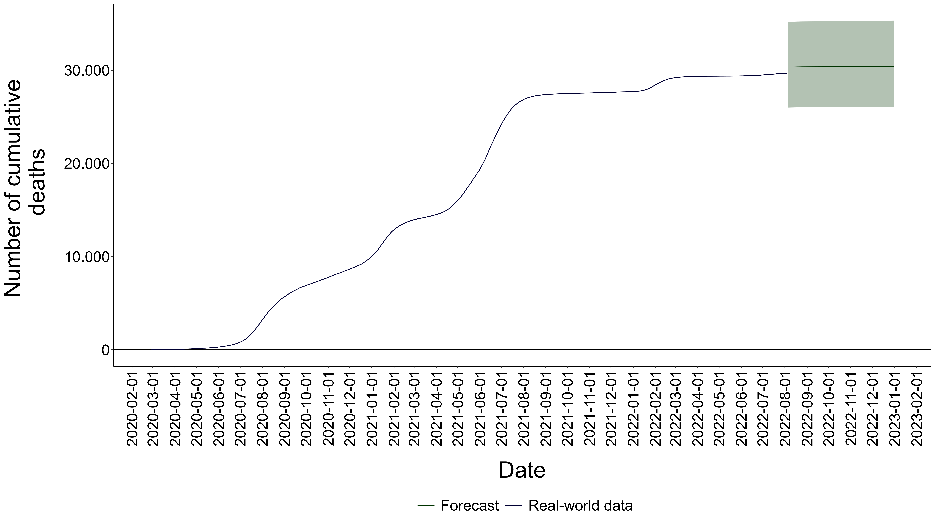 | (D)  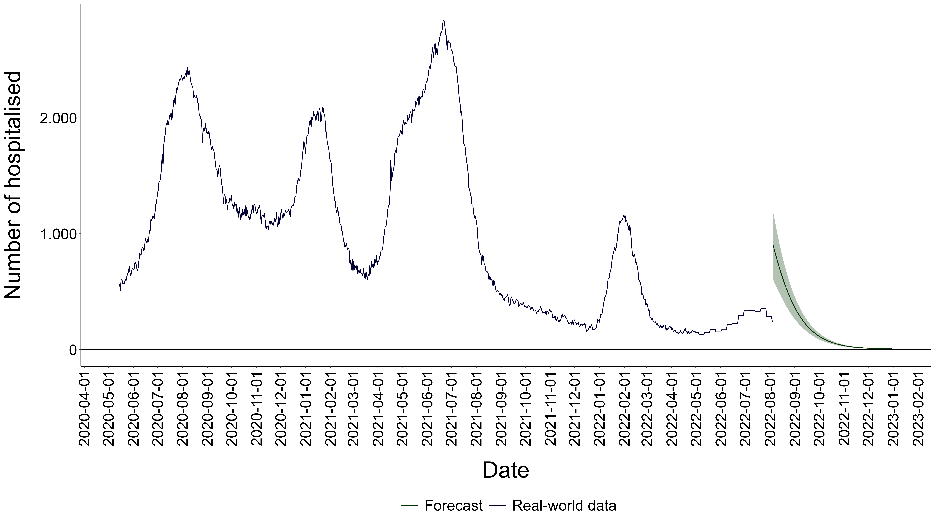 |
|  | (E)  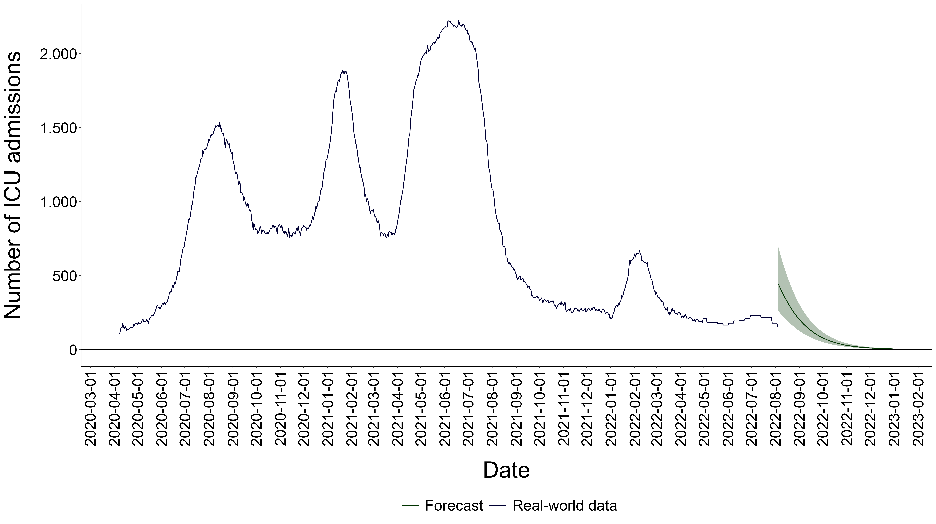 | |

**Figure 1.** Predicted health outcomes of SARS-CoV-2 in Bogotá. (A) Number of new cases, (B) Number of symptomatic, (C) Number of cumulative deaths, (D) Number of hospitalised, (E) Number of ICU admissions

Source: own elaboration.

**Scenario 1B**

|  | (A)  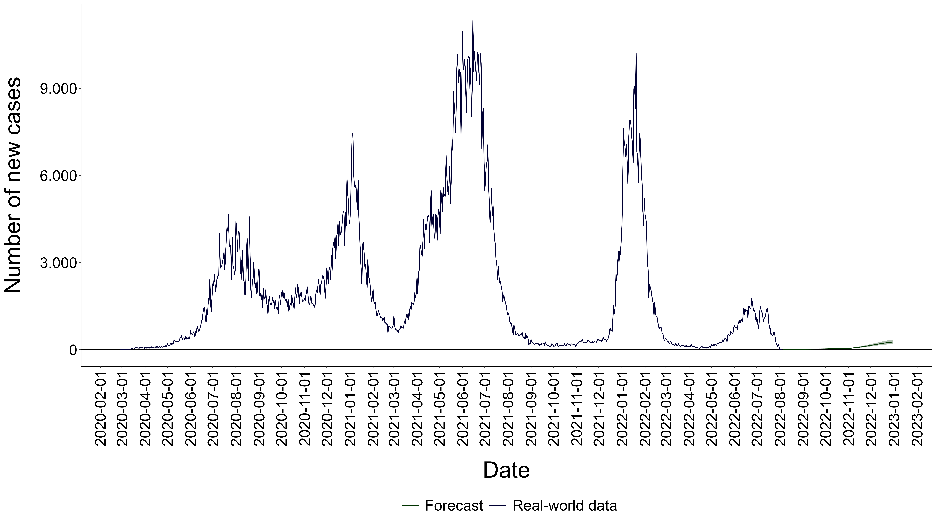 | (B)  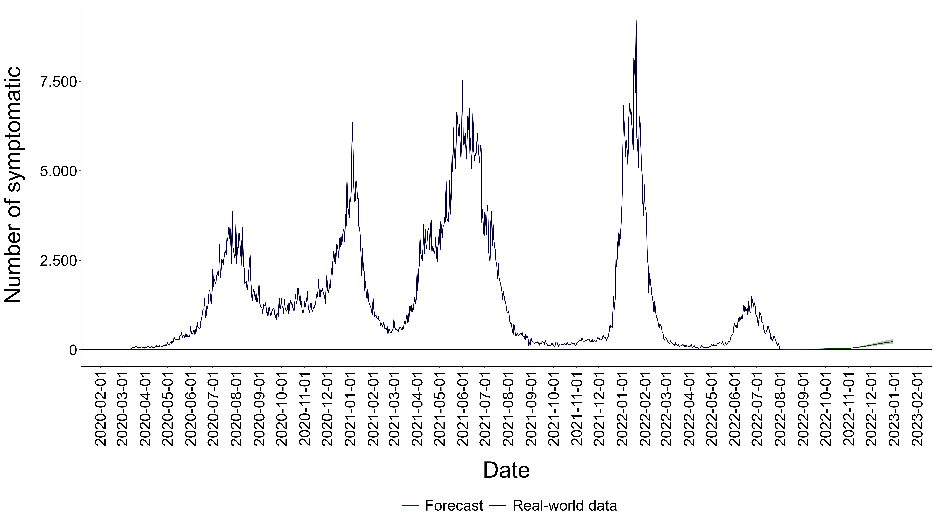 |
| --- | --- | --- |
|  | (C)  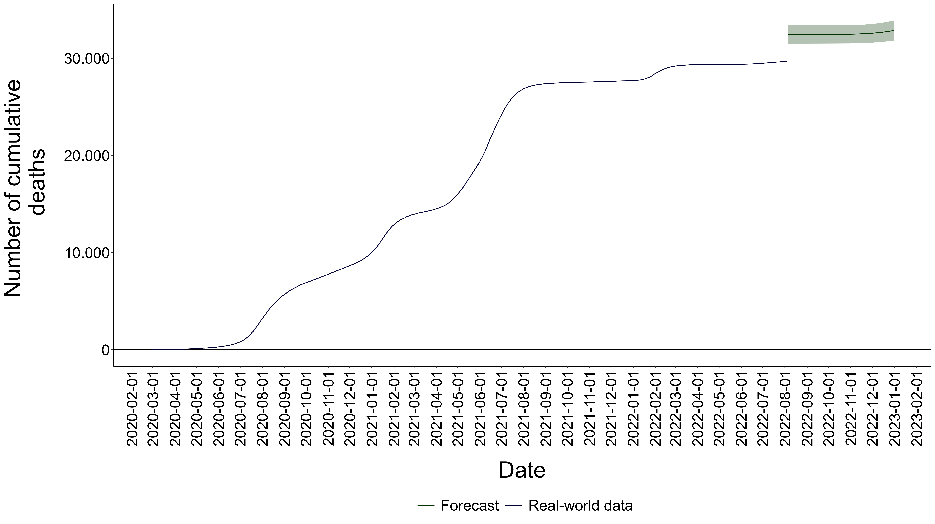 | (D)  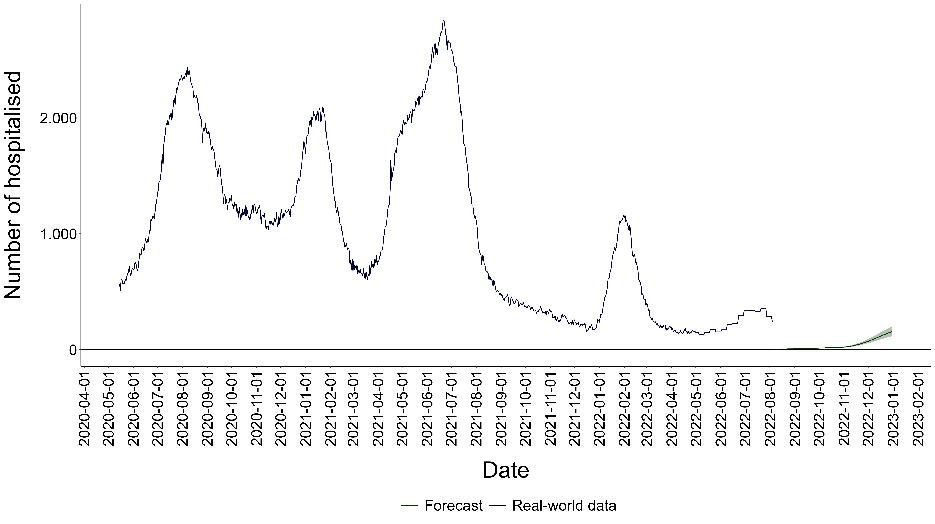 |
|  | (E)  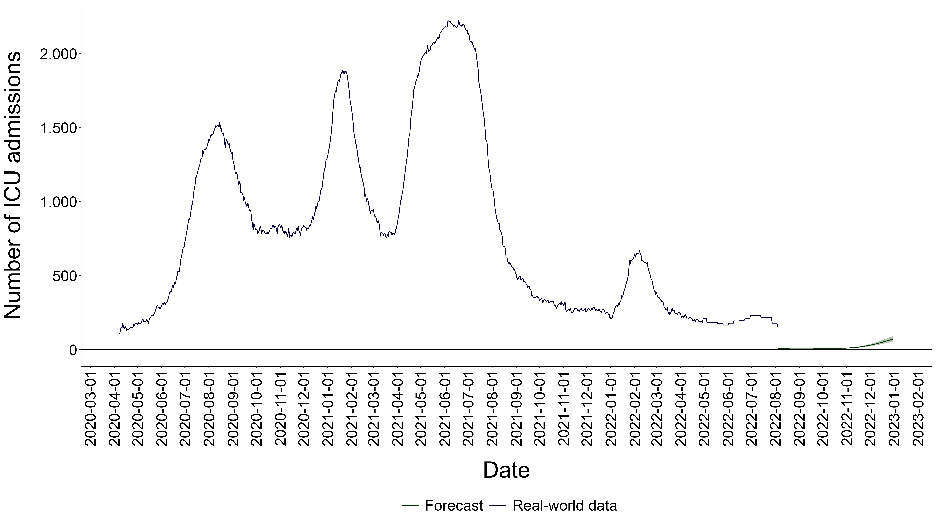 | |

**Figure 2.** Predicted health outcomes of SARS-CoV-2 in Bogotá. (A) Number of new cases, (B) Number of symptomatic, (C) Number of cumulative deaths, (D) Number of hospitalised, (E) Number of ICU admissions

Source: own elaboration.

**Scenario 1C**

|  | (A)  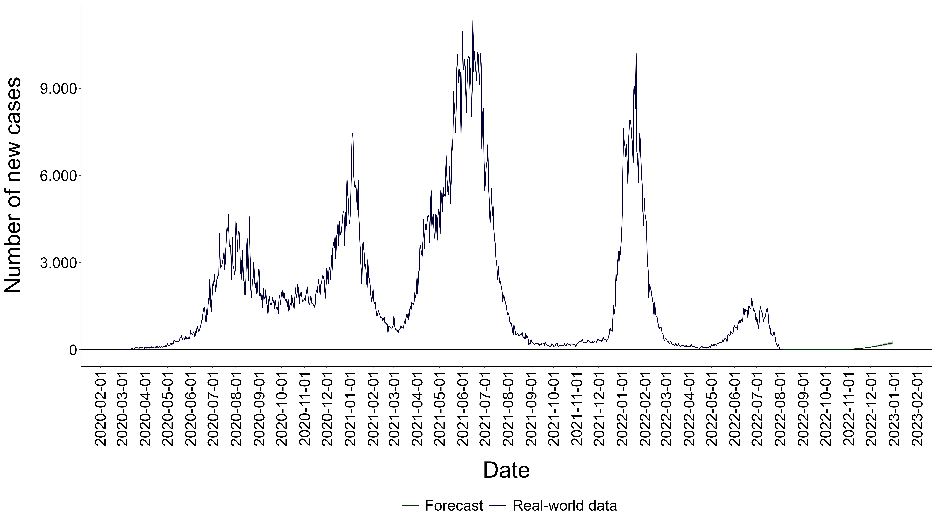 | (B)  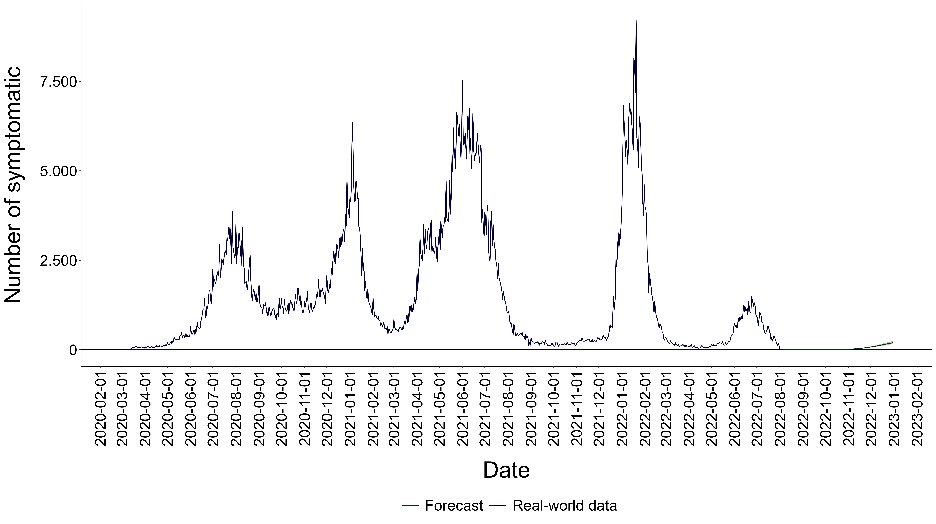 |
| --- | --- | --- |
|  | (C)  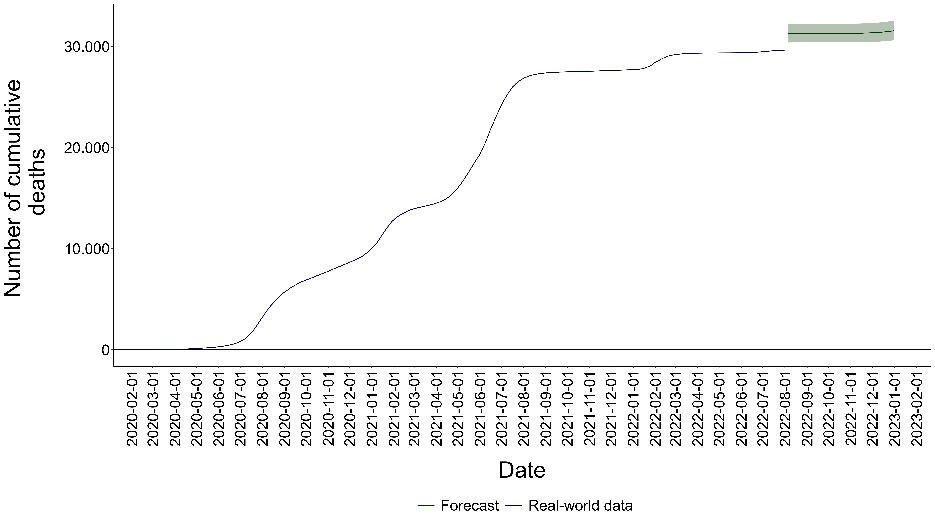 | (D)  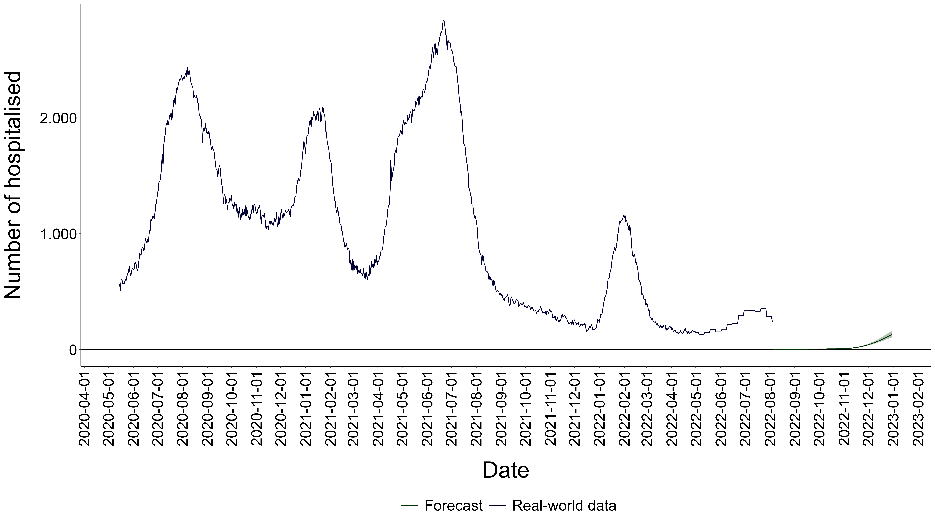 |
|  | (E)  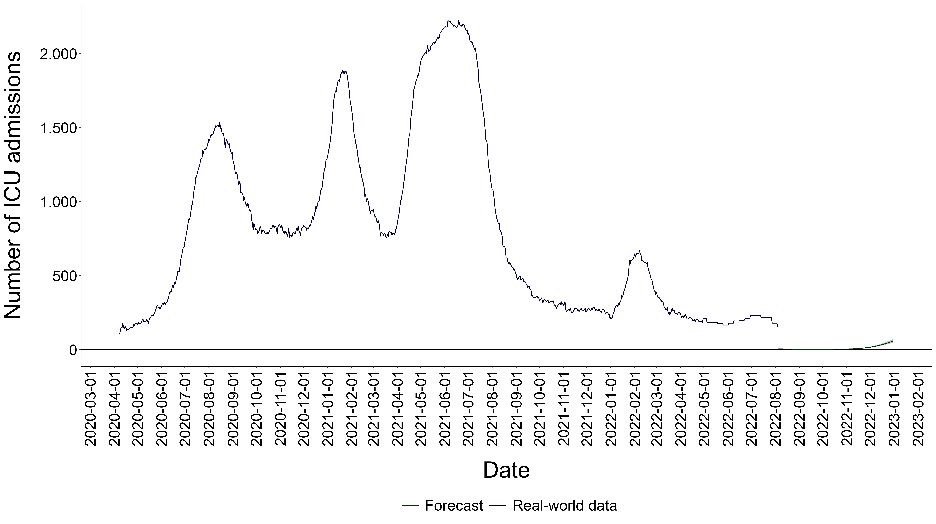 | |

**Figure 3.** Predicted health outcomes of SARS-CoV-2 in Bogotá. (A) Number of new cases, (B) Number of symptomatic, (C) Number of cumulative deaths, (D) Number of hospitalised, (E) Number of ICU admissions

Source: own elaboration.

**Scenario 1D**

|  | (A)  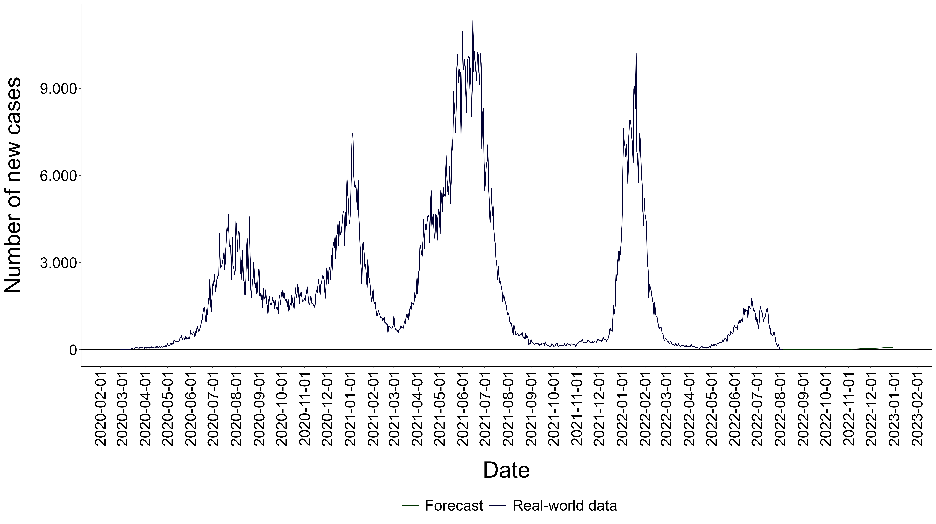 | (B)  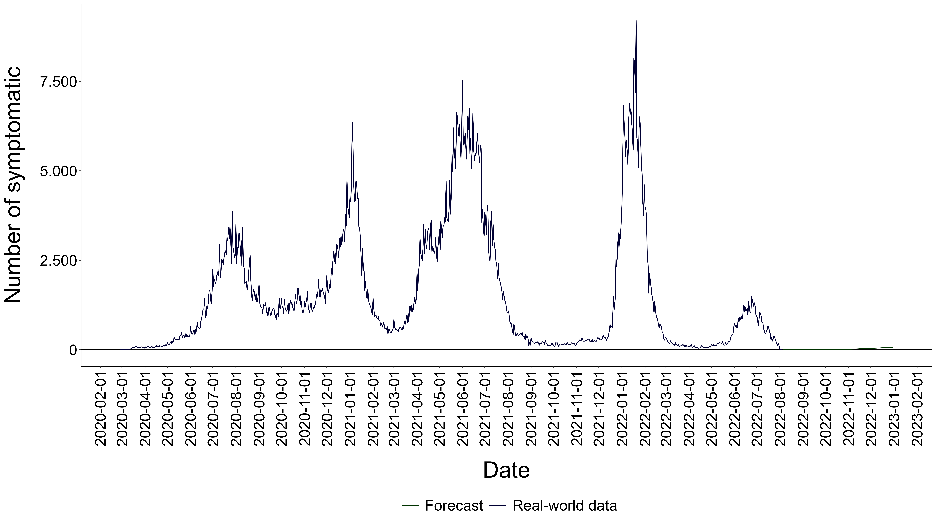 |
| --- | --- | --- |
|  | (C)  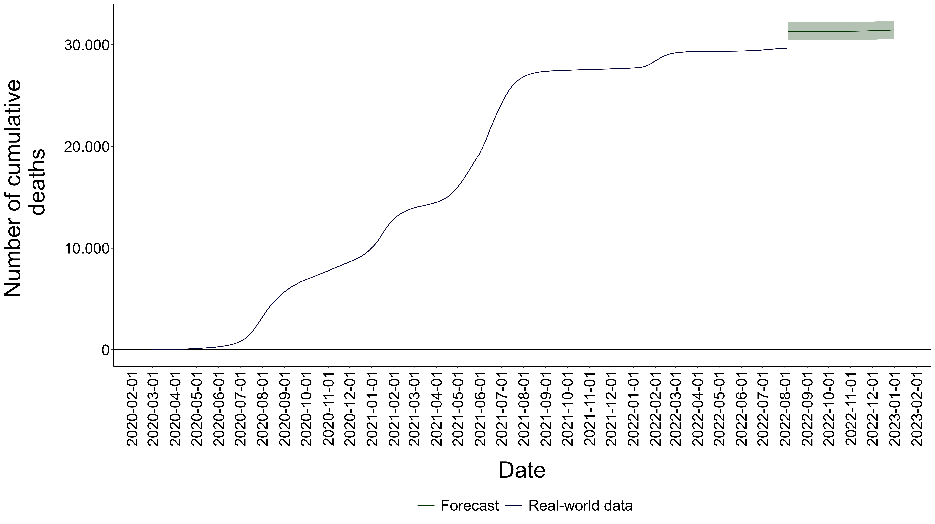 | (D)  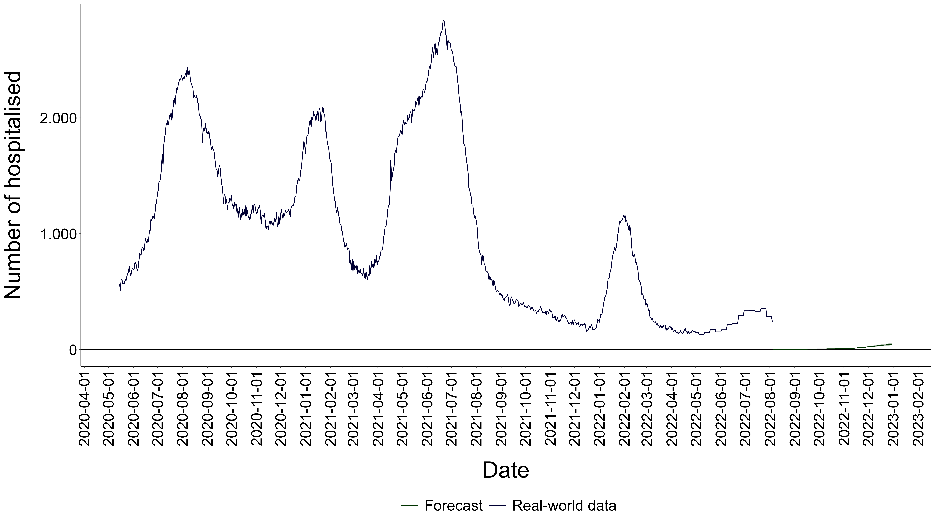 |
|  | (E)  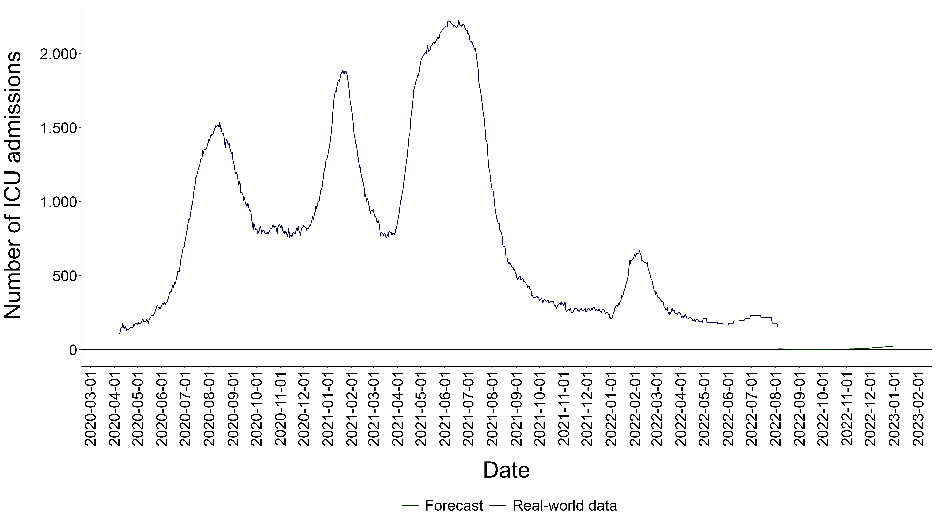 | |

**Figure 4.** Predicted health outcomes of SARS-CoV-2 in Bogotá. (A) Number of new cases, (B) Number of symptomatic, (C) Number of cumulative deaths, (D) Number of hospitalised, (E) Number of ICU admissions

Source: own elaboration.

**Scenario 2: Low efficacy (and effectiveness) of the vaccine, moderate non-pharmacological measures**

**Scenario 2A**

|  | (A)  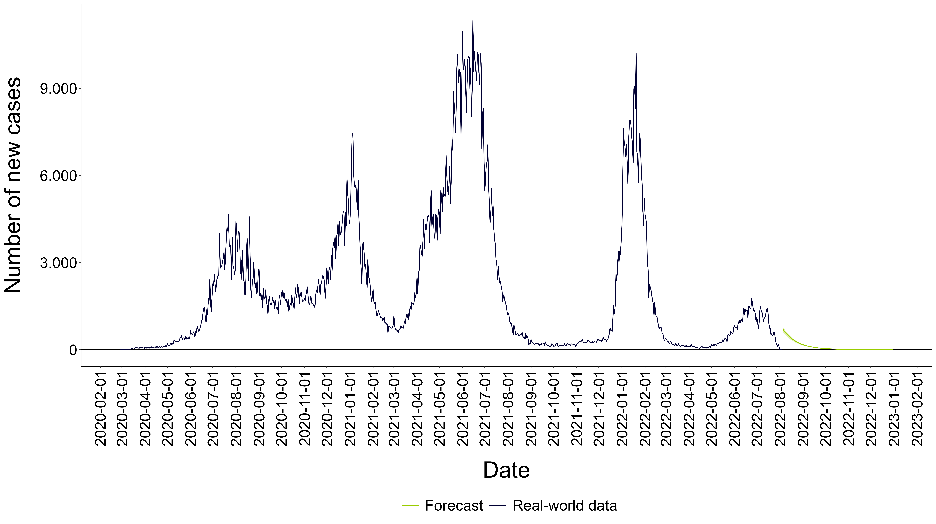 | (B)  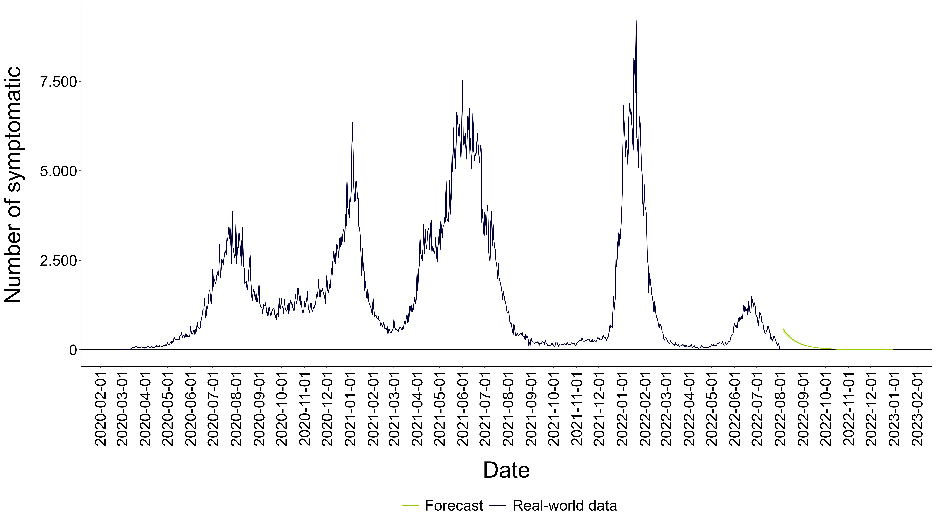 |
| --- | --- | --- |
|  | (C)  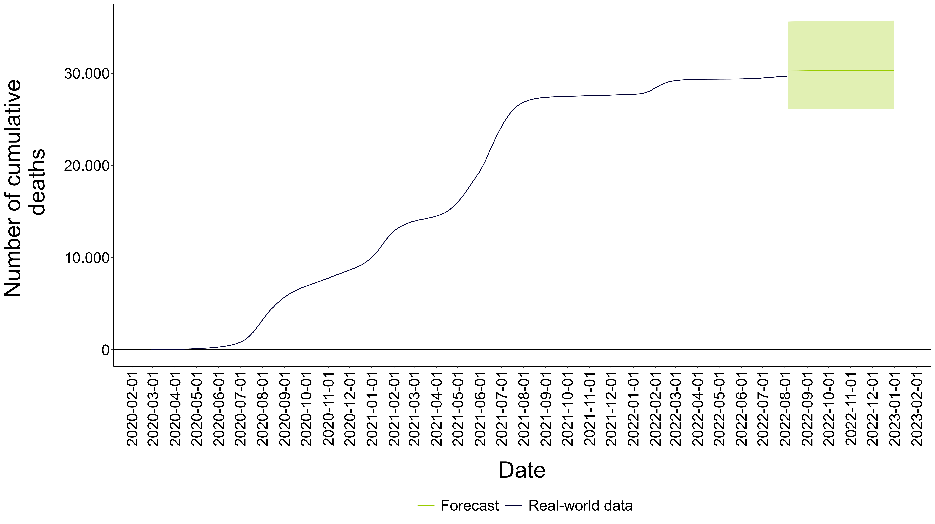 | (D)  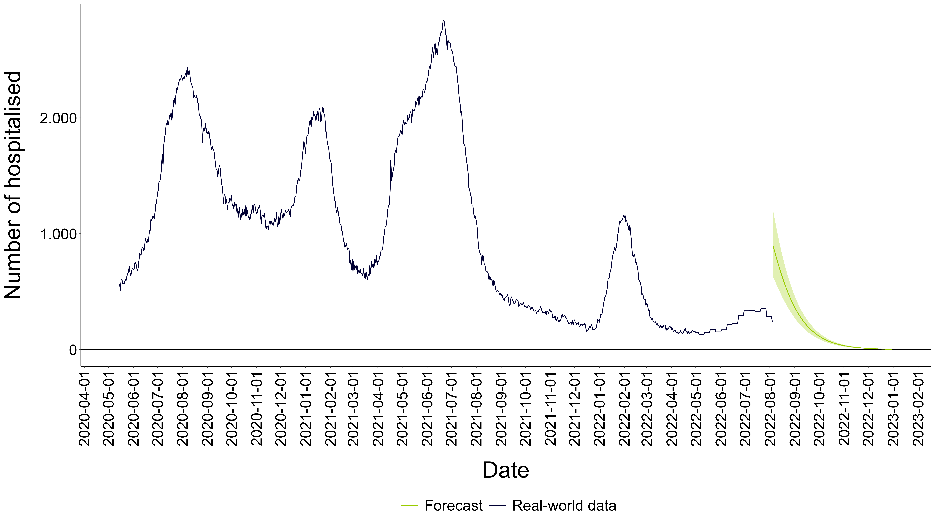 |
|  | (E)  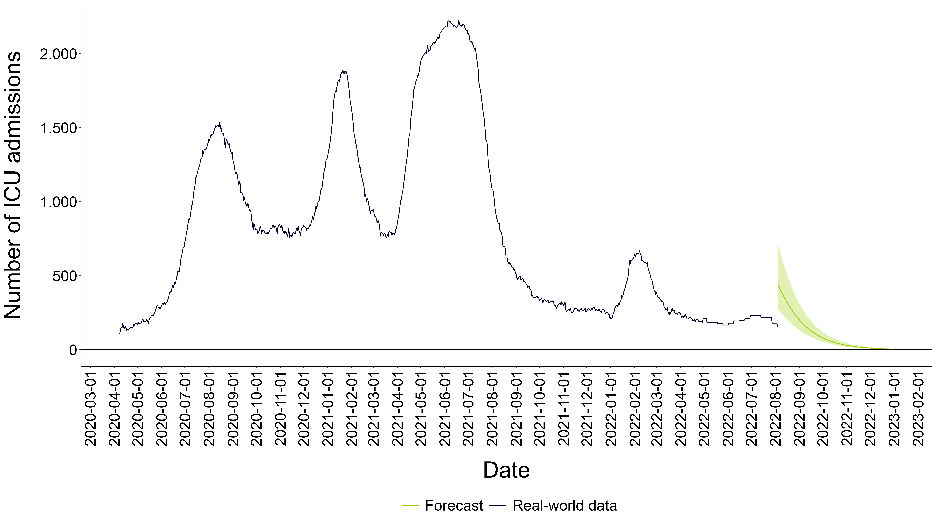 | |

**Figure 5.** Predicted health outcomes of SARS-CoV-2 in Bogotá. (A) Number of new cases, (B) Number of symptomatic, (C) Number of cumulative deaths, (D) Number of hospitalised, (E) Number of ICU admissions

Source: own elaboration.

**Scenario 2B**

|  | (A)  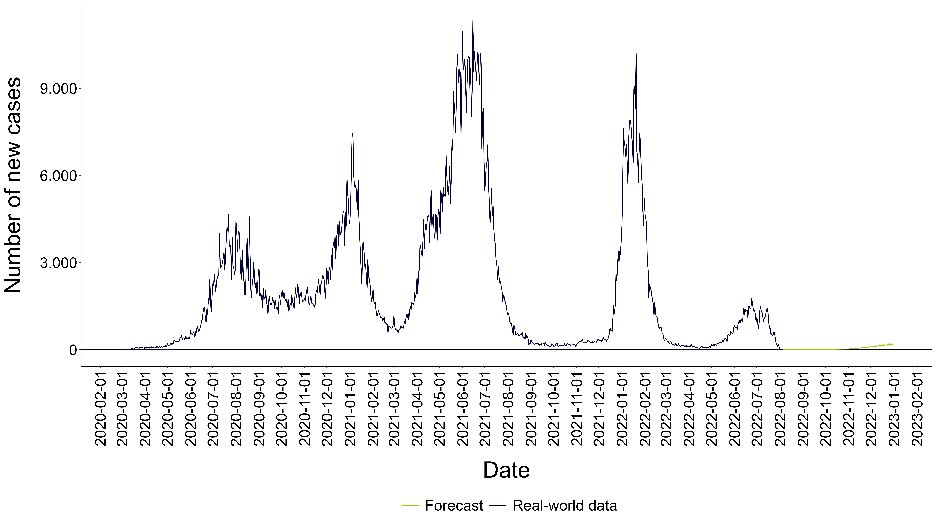 | (B)  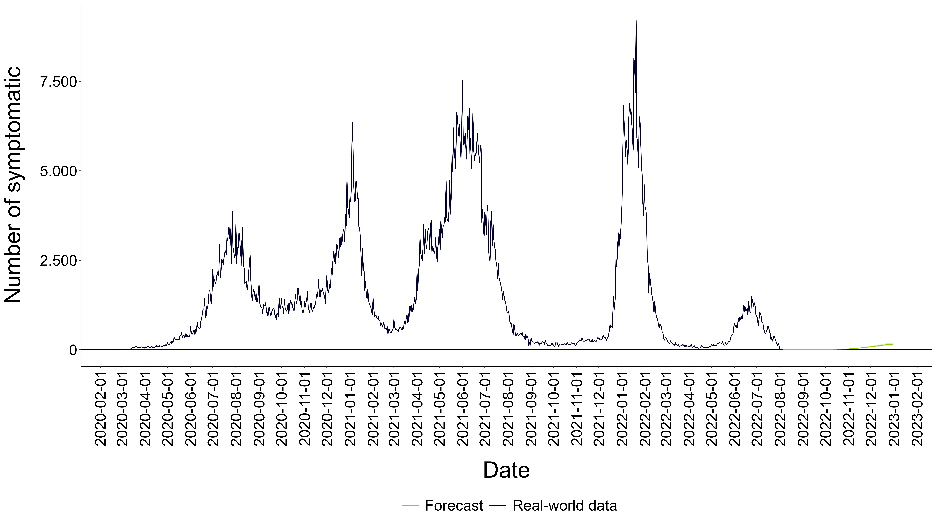 |
| --- | --- | --- |
|  | (C)  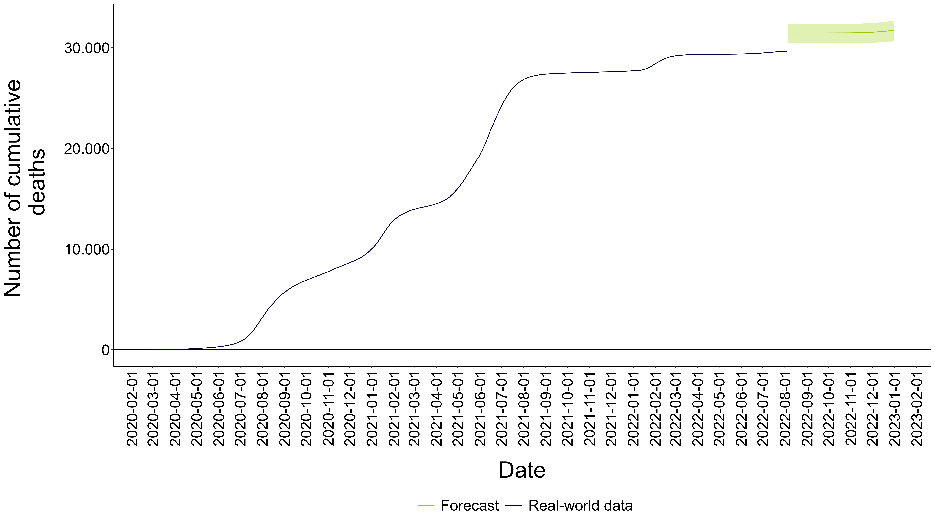 | (D)  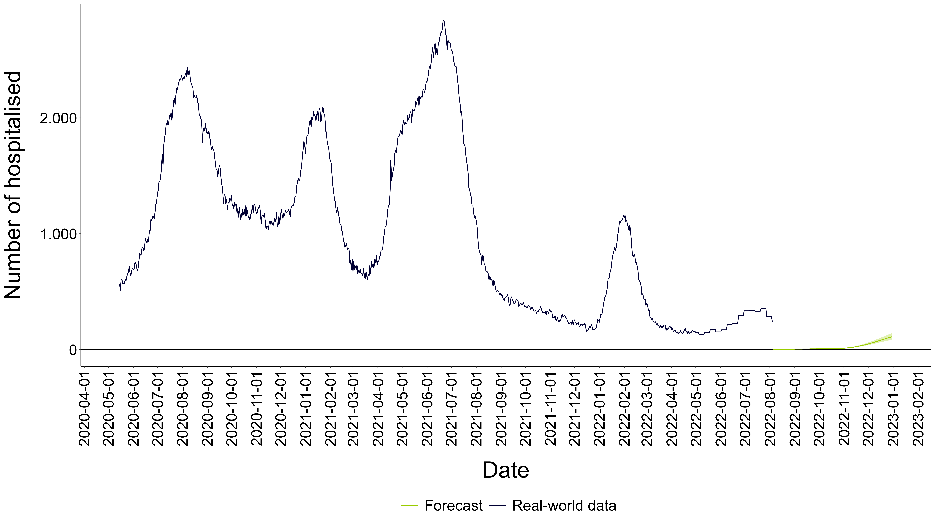 |
|  | (E)  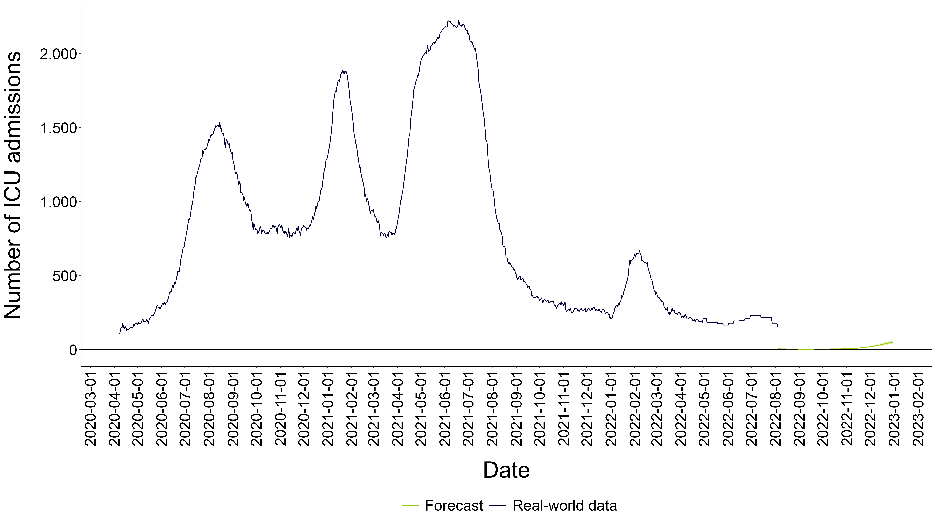 | |

**Figure 6.** Predicted health outcomes of SARS-CoV-2 in Bogotá. (A) Number of new cases, (B) Number of symptomatic, (C) Number of cumulative deaths, (D) Number of hospitalised, (E) Number of ICU admissions

Source: own elaboration.

**Scenario 2C**

|  | (A)  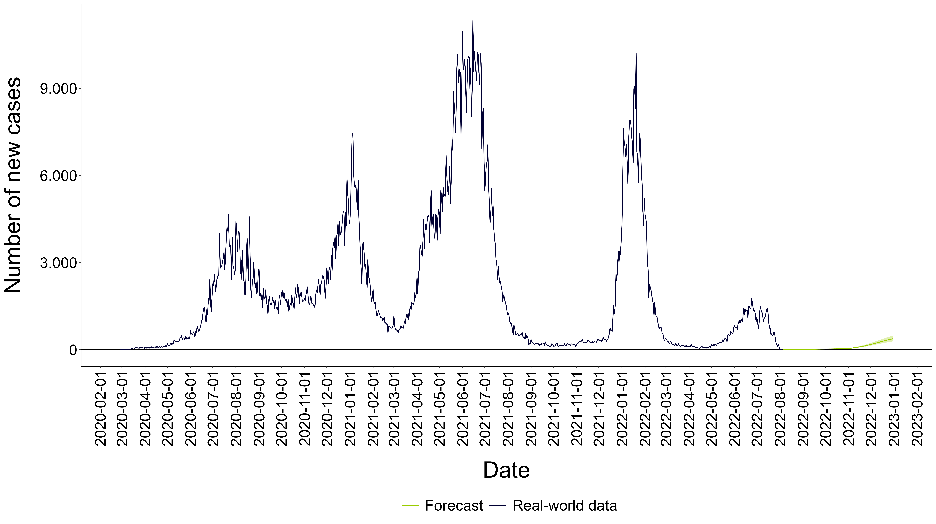 | (B)  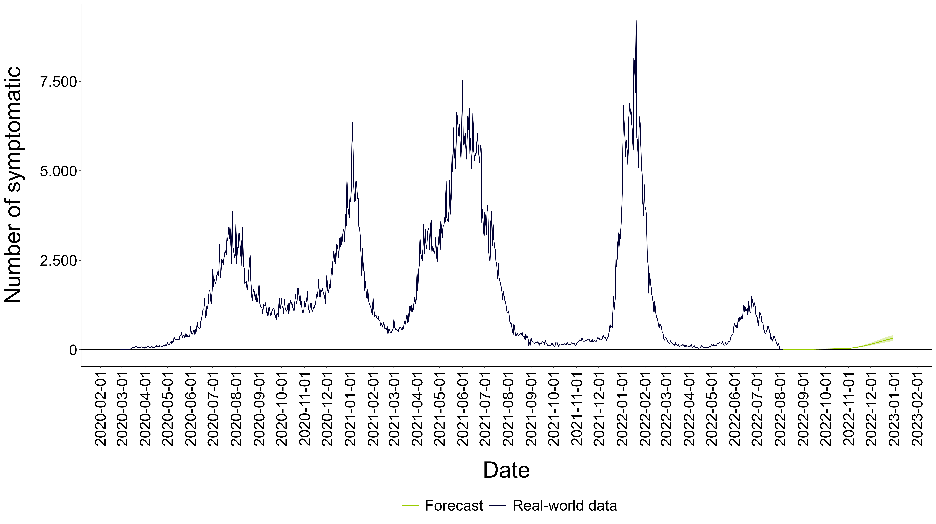 |
| --- | --- | --- |
|  | (C)  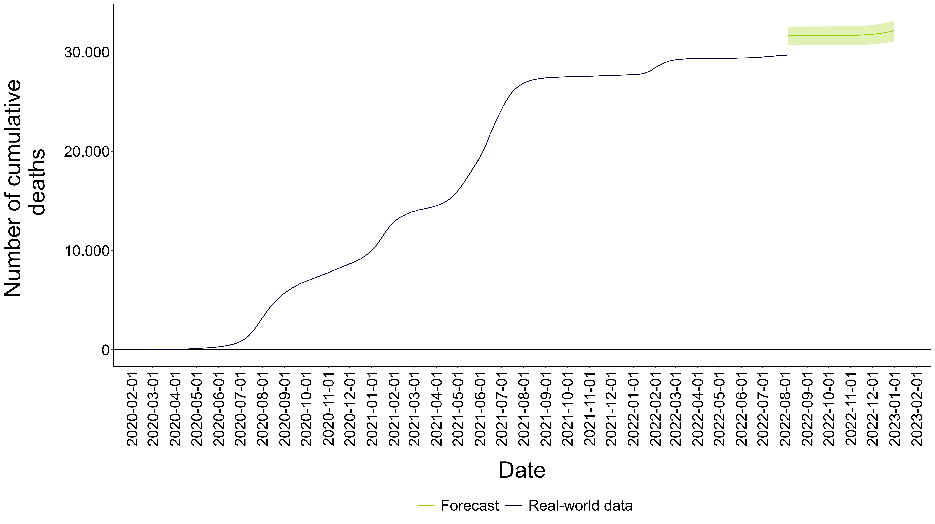 | (D)  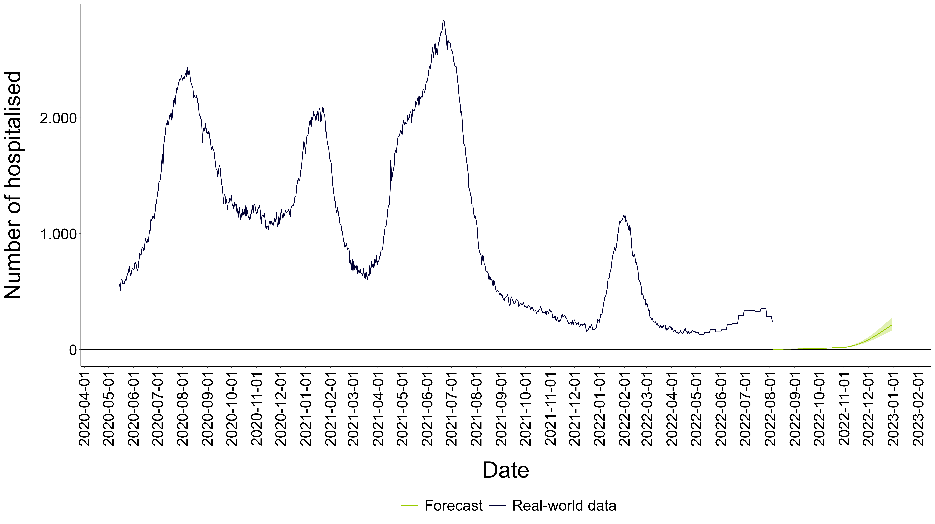 |
|  | (E)  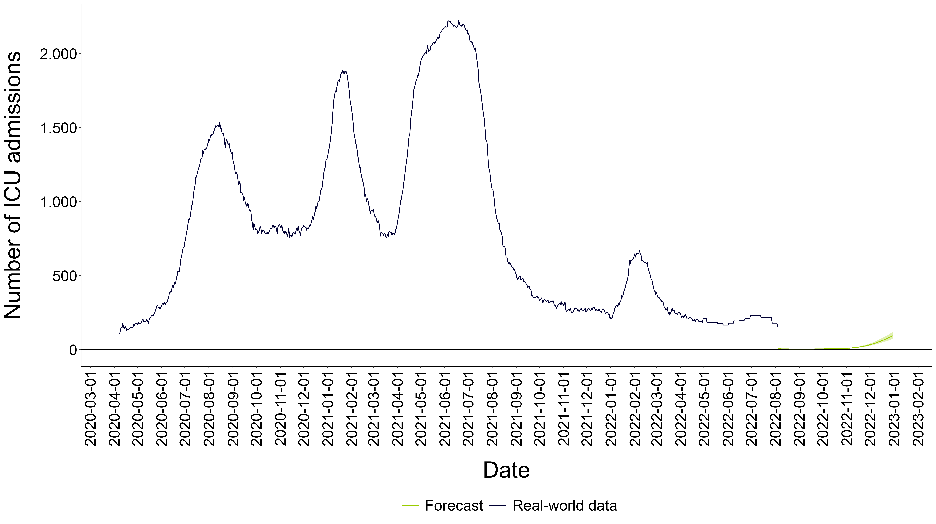 | |

**Figure 7.** Predicted health outcomes of SARS-CoV-2 in Bogotá. (A) Number of new cases, (B) Number of symptomatic, (C) Number of cumulative deaths, (D) Number of hospitalised, (E) Number of ICU admissions

Source: own elaboration.

**Scenario 2D**

|  | (A)  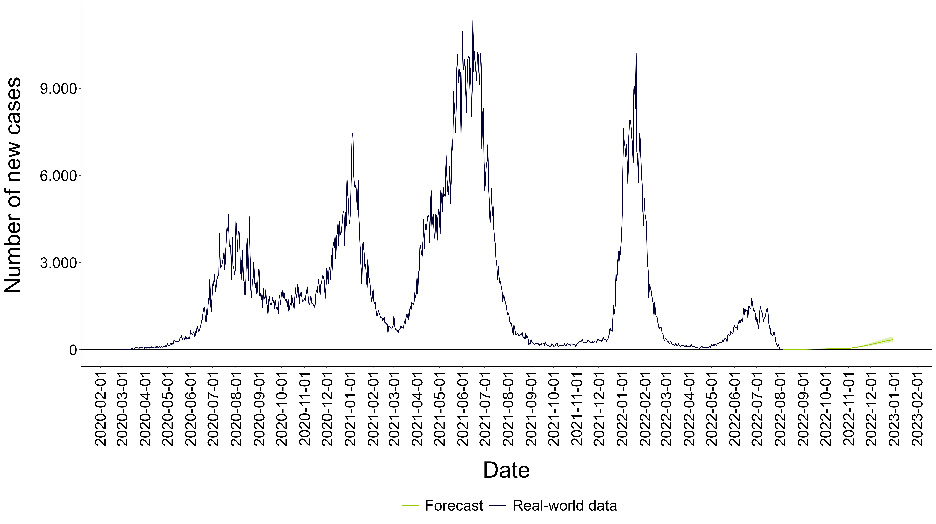 | (B)  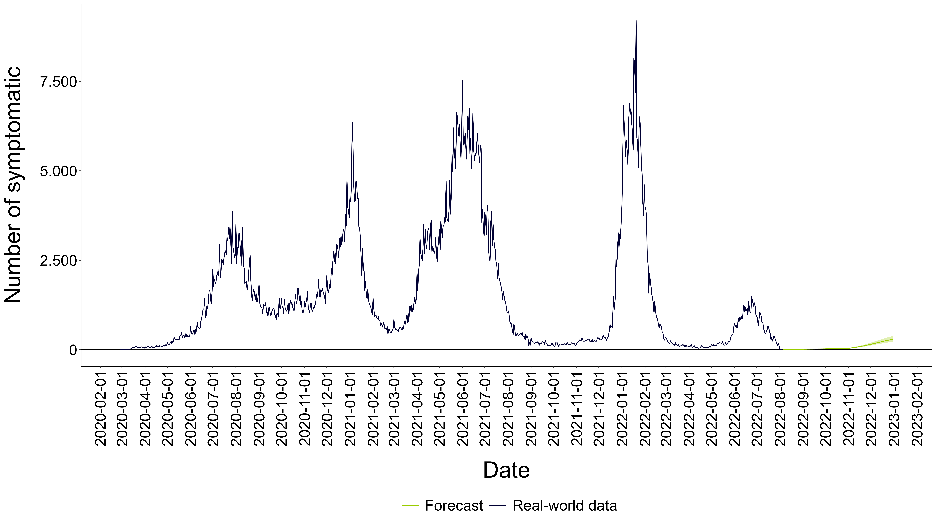 |
| --- | --- | --- |
|  | (C)  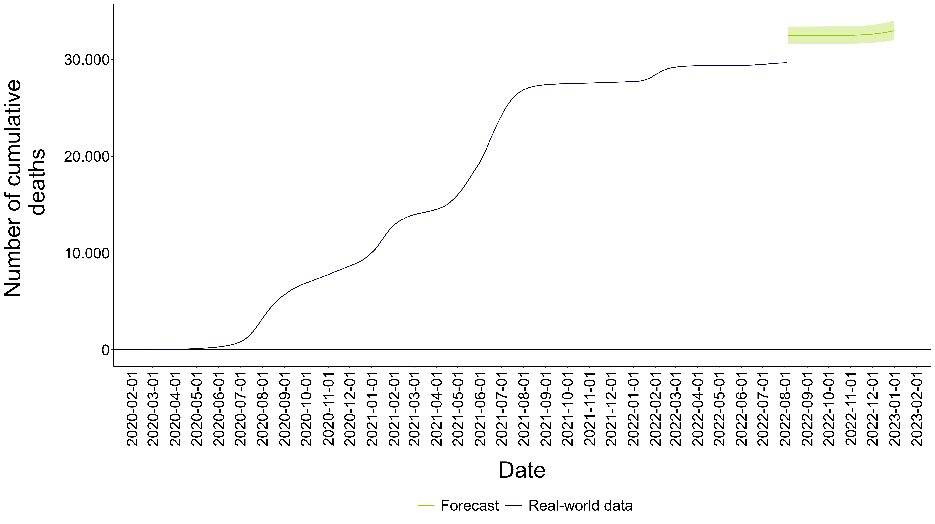 | (D)  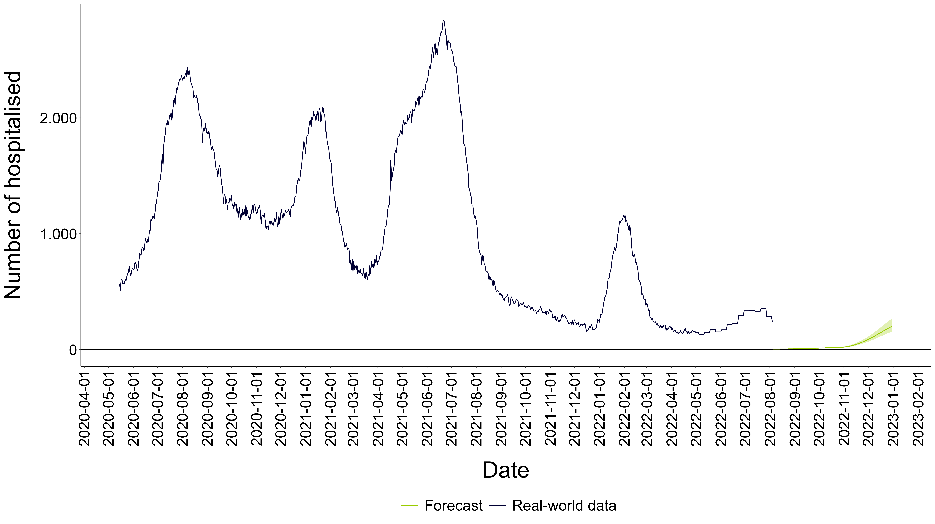 |
|  | (E)  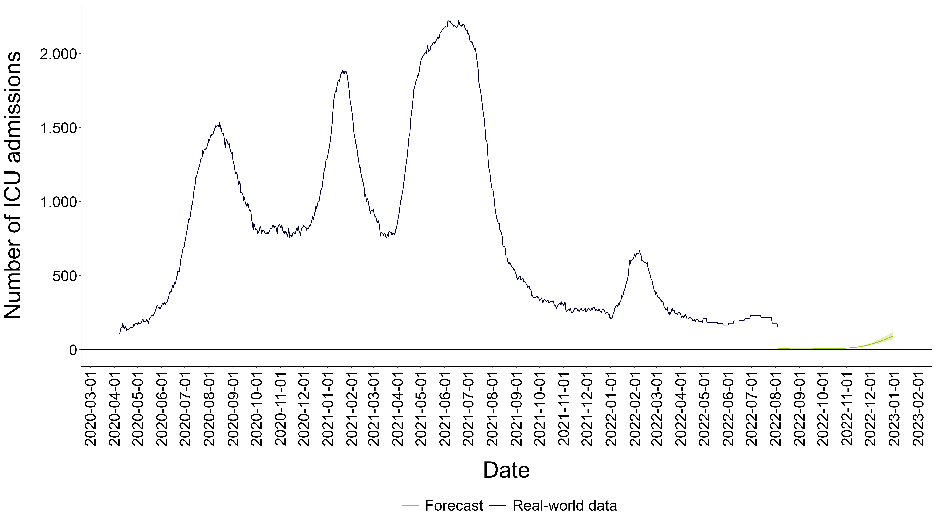 | |

**Figure 8.** Predicted health outcomes of SARS-CoV-2 in Bogotá. (A) Number of new cases, (B) Number of symptomatic, (C) Number of cumulative deaths, (D) Number of hospitalised, (E) Number of ICU admissions

Source: own elaboration.

**Scenario 3: High efficacy (and effectiveness) of the vaccine, relaxation of non-pharmacological measures**

**Scenario 3A**

|  | (A)  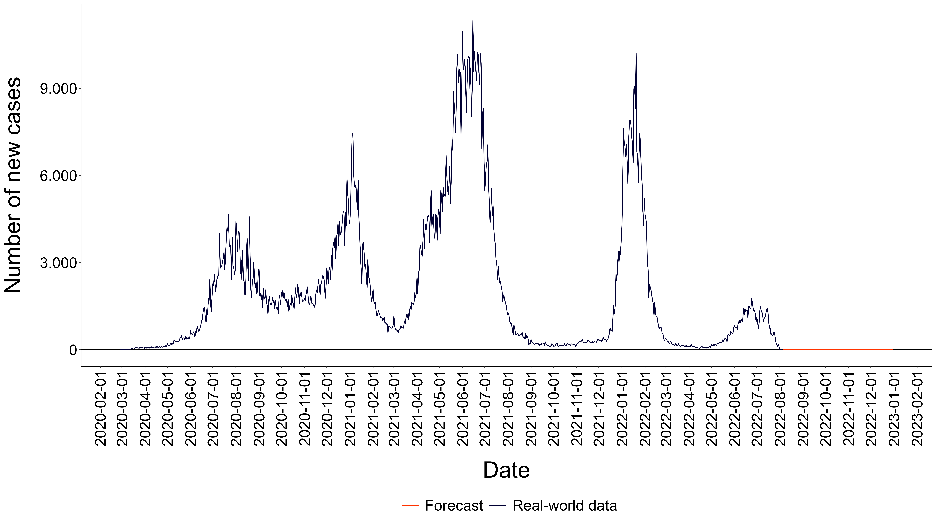 | (B)  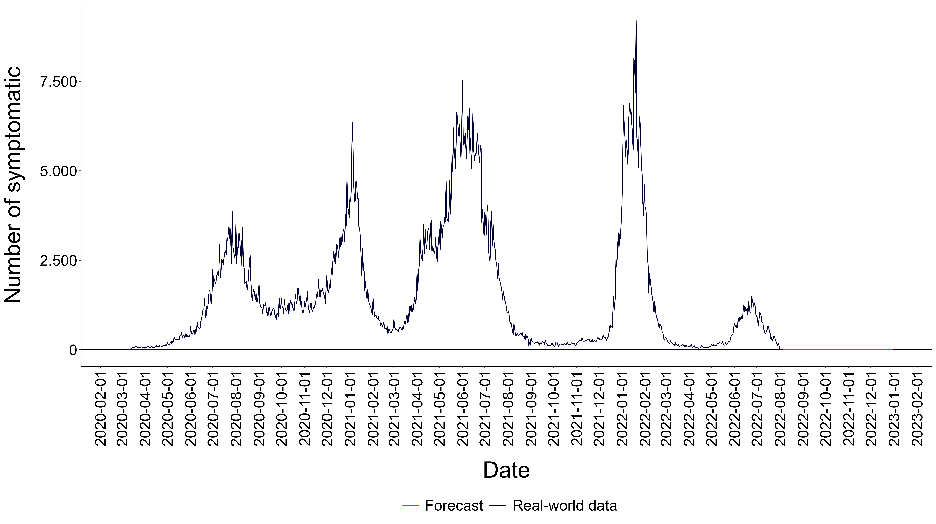 |
| --- | --- | --- |
|  | (C)  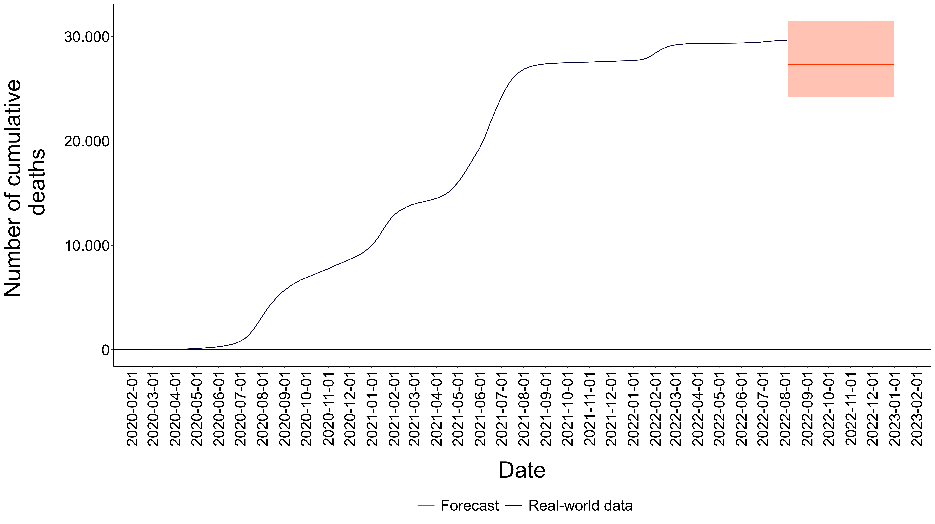 | (D)  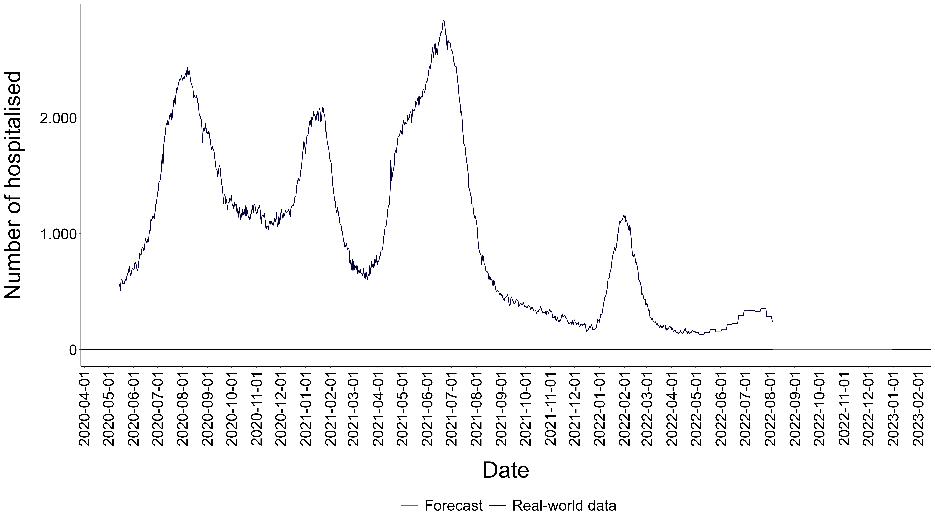 |
|  | (E)  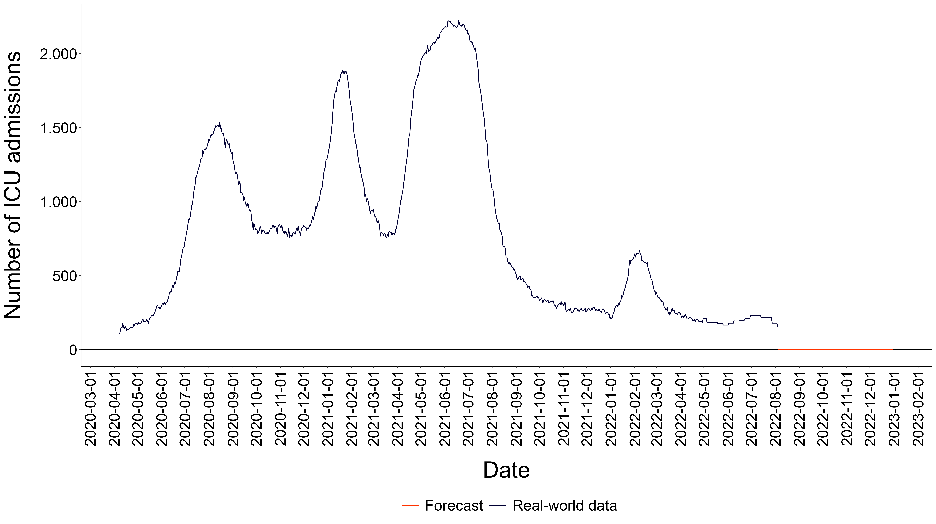 | |

**Figure 9**. Predicted health outcomes of SARS-CoV-2 in Bogotá. (A) Number of new cases, (B) Number of symptomatic, (C) Number of cumulative deaths, (D) Number of hospitalised, (E) Number of ICU admissions

Source: own elaboration.

**Scenario 3B**

|  | (A)  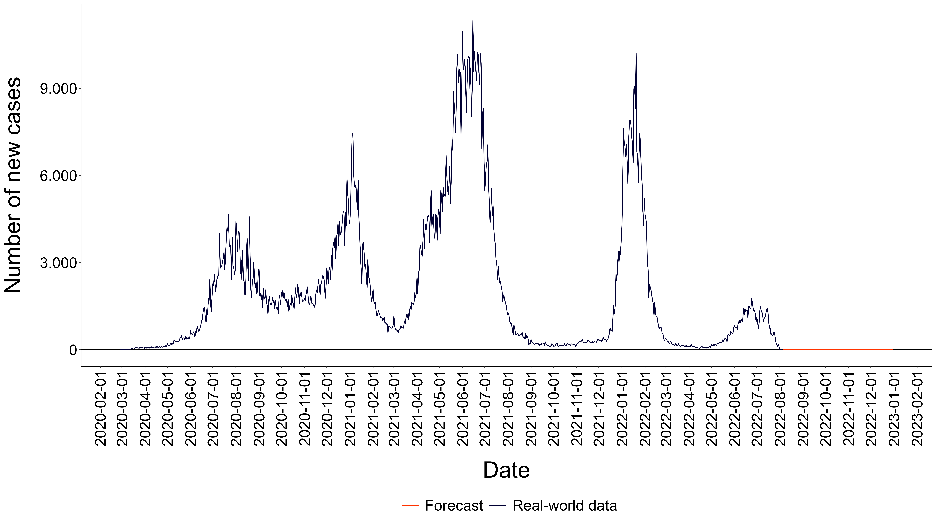 | (B)  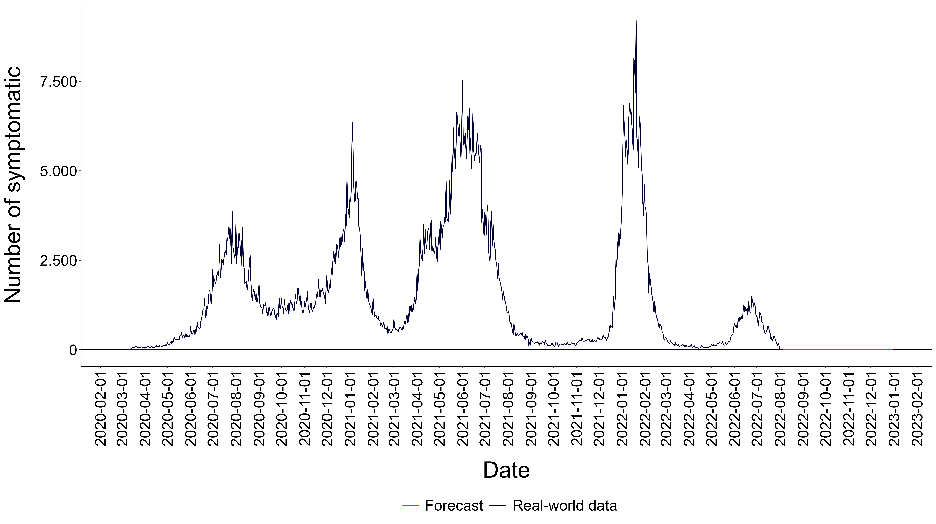 |
| --- | --- | --- |
|  | (C)  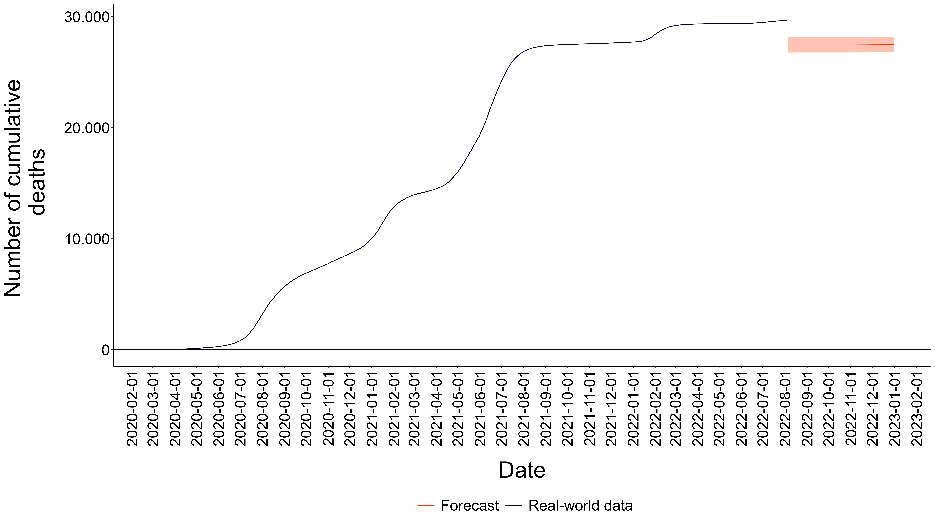 | (D)  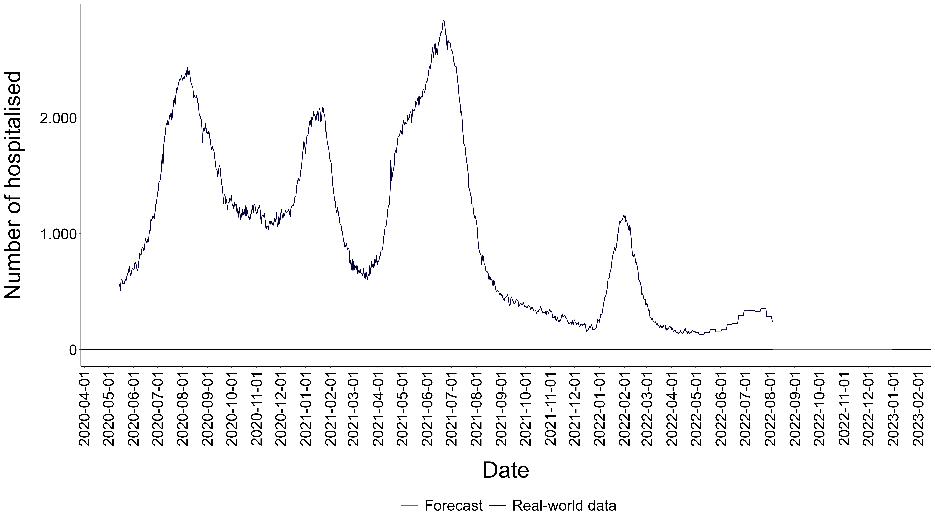 |
|  | (E)  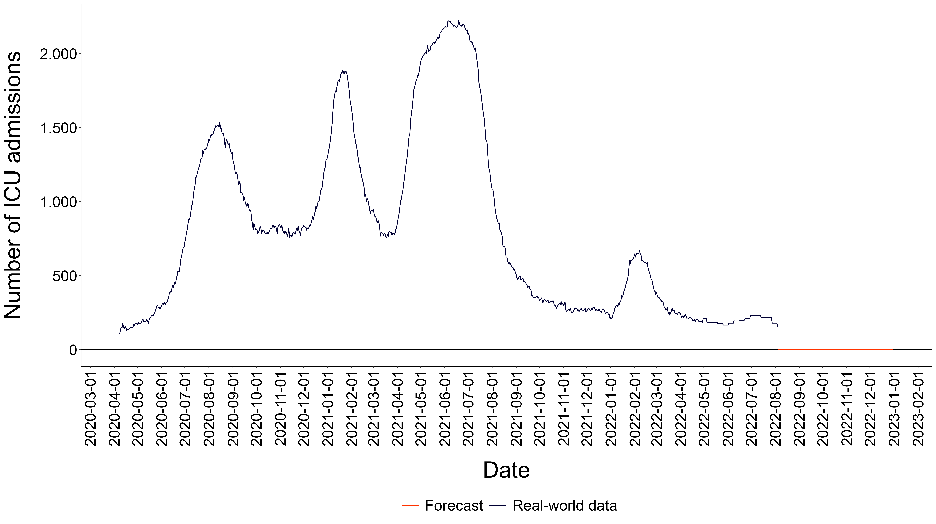 | |

**Figure 10**. Predicted health outcomes of SARS-CoV-2 in Bogotá. (A) Number of new cases, (B) Number of symptomatic, (C) Number of cumulative deaths, (D) Number of hospitalised, (E) Number of ICU admissions

Source: own elaboration.

**Scenario 3C**

|  | (A)  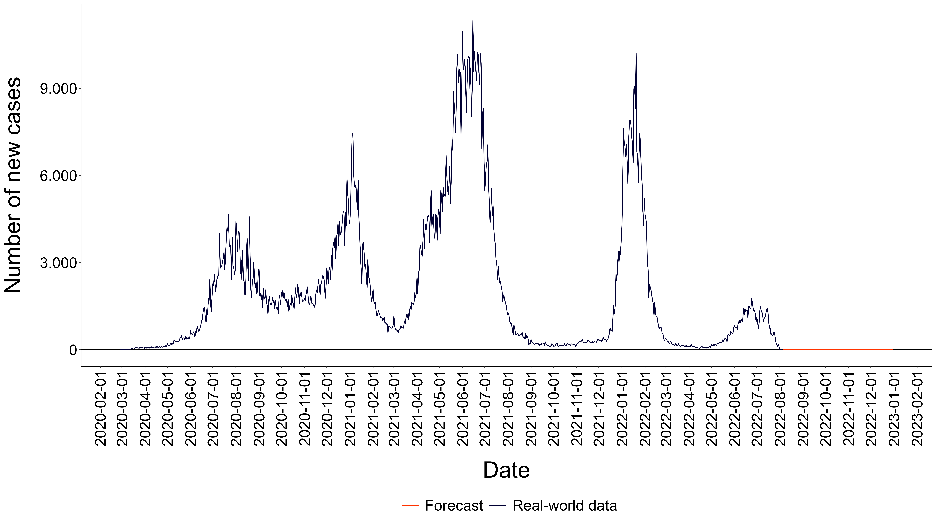 | (B)  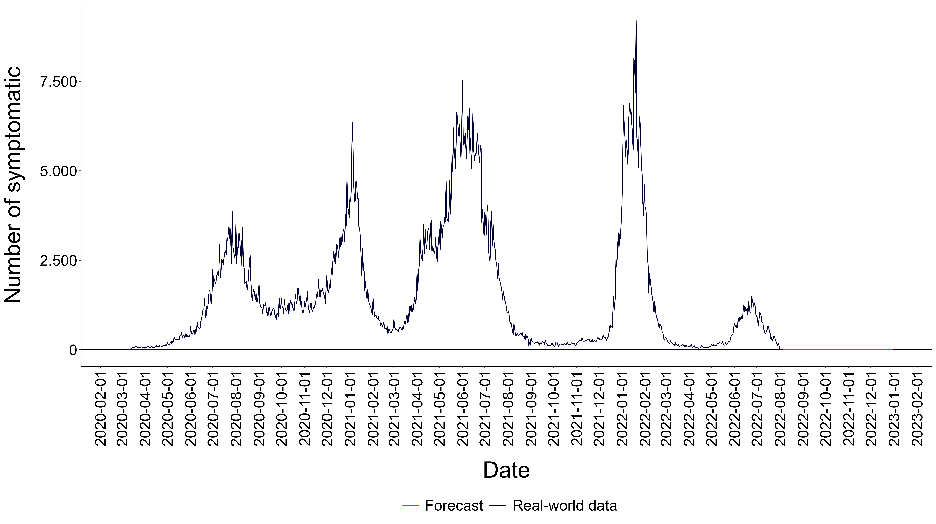 |
| --- | --- | --- |
|  | (C)  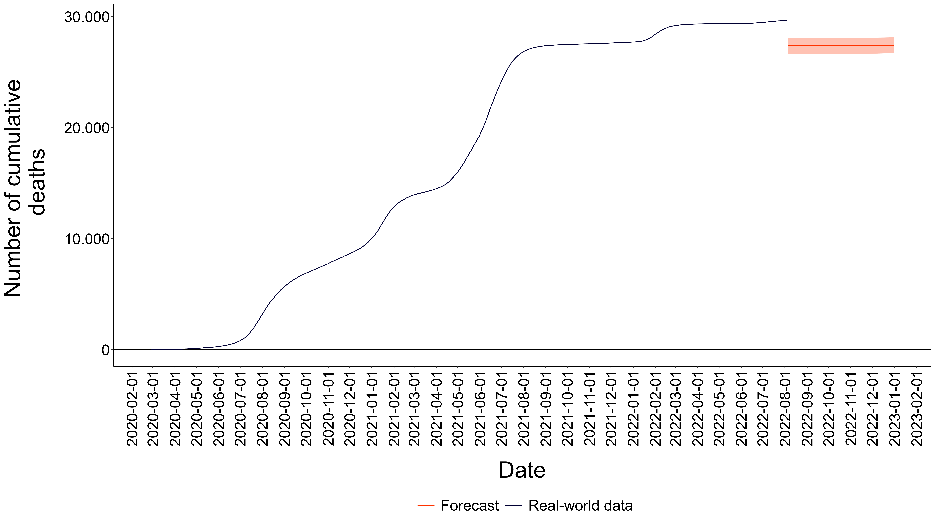 | (D)  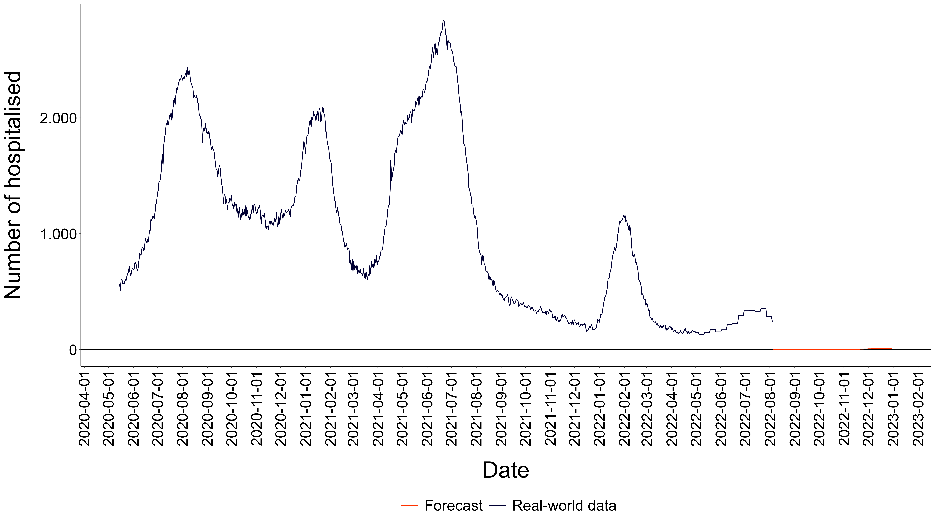 |
|  | (E)  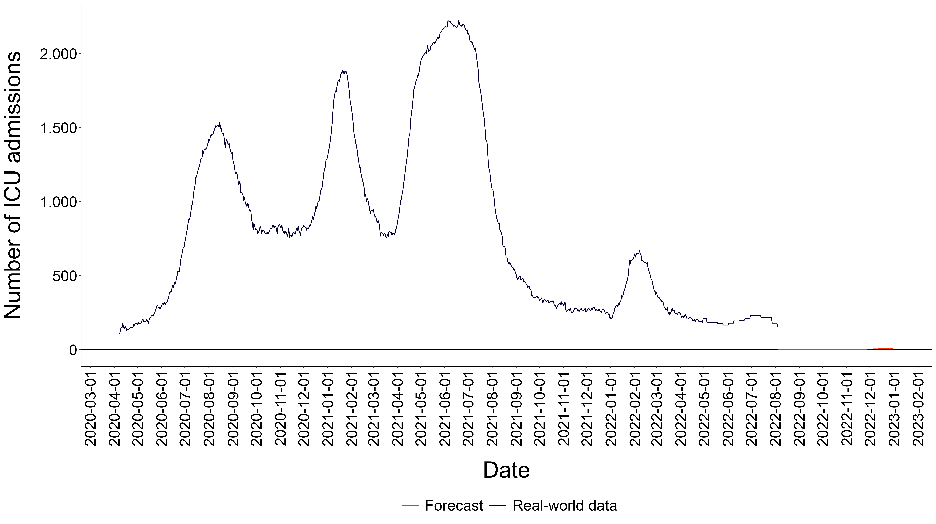 | |

**Figure 11**. Predicted health outcomes of SARS-CoV-2 in Bogotá. (A) Number of new cases, (B) Number of symptomatic, (C) Number of cumulative deaths, (D) Number of hospitalised, (E) Number of ICU admissions

Source: own elaboration.

**Scenario 3D**

|  | (A)  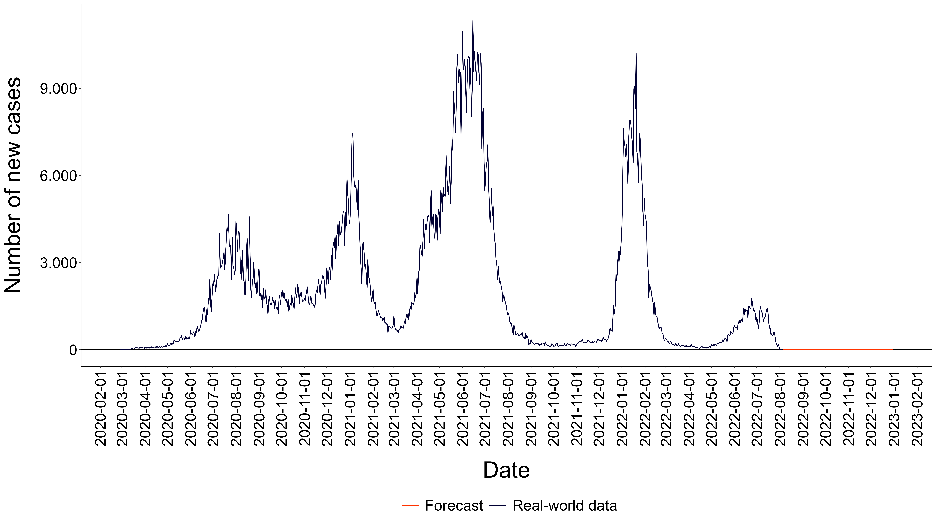 | (B)  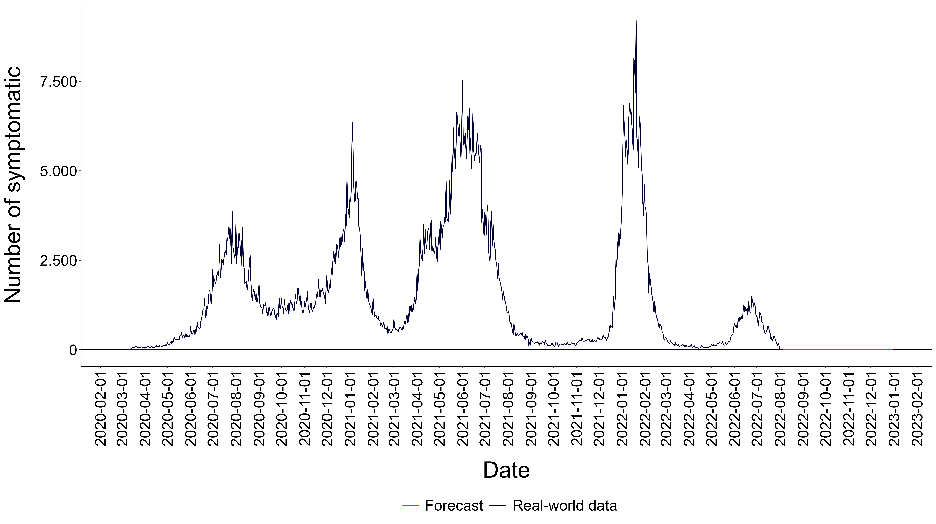 |
| --- | --- | --- |
|  | (C)  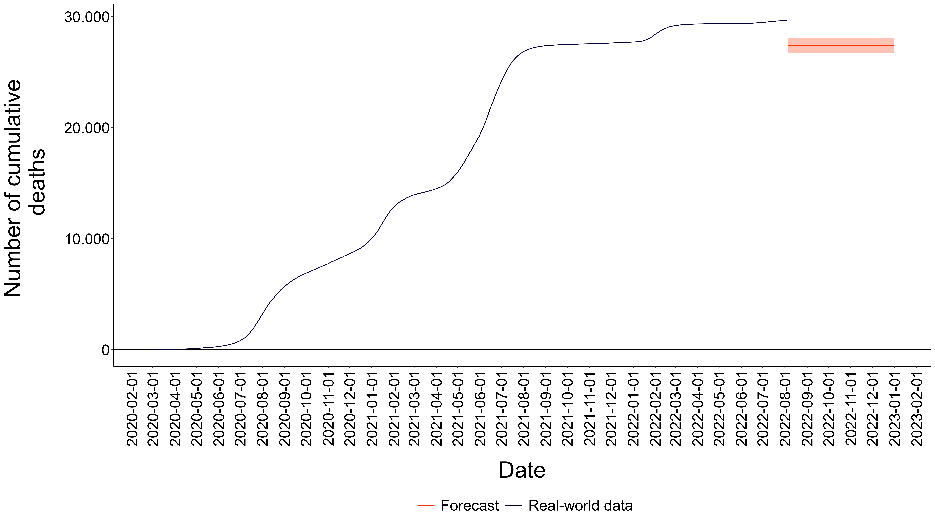 | (D)  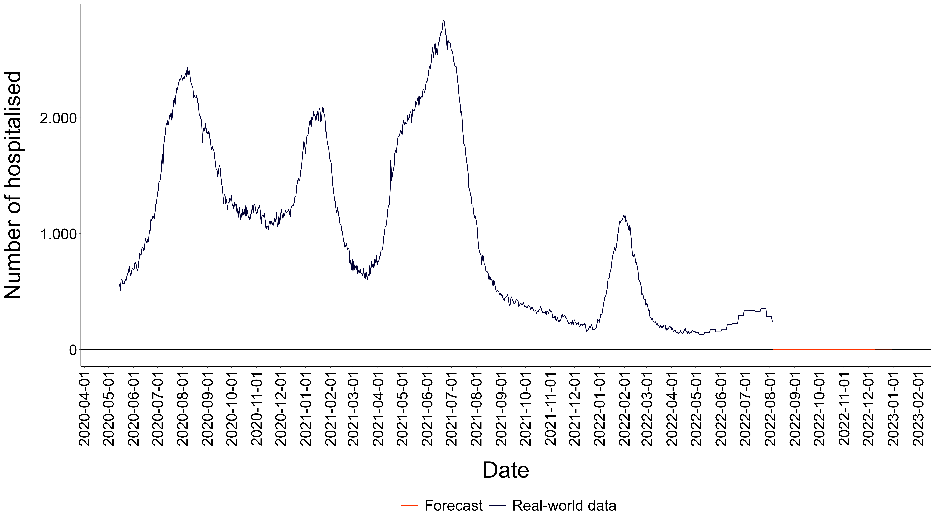 |
|  | (E)  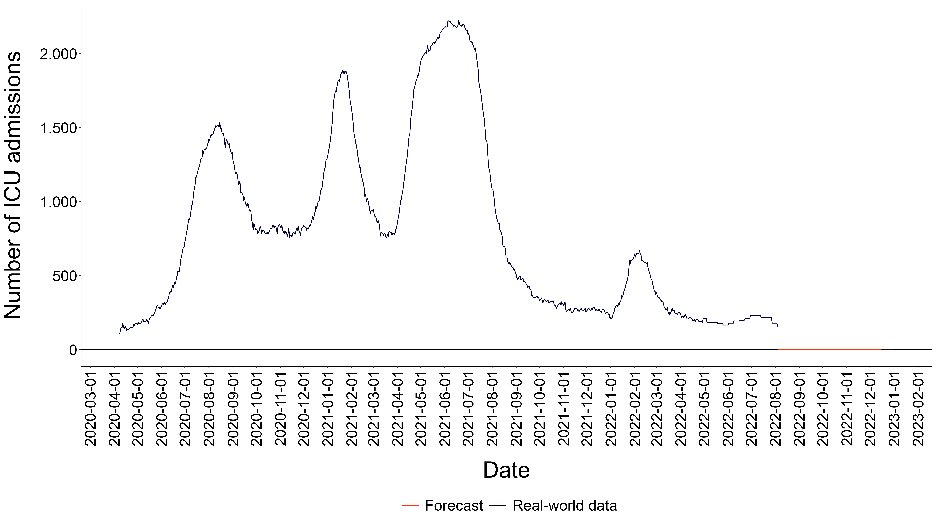 | |

**Figure 12**. Predicted health outcomes of SARS-CoV-2 in Bogotá. (A) Number of new cases, (B) Number of symptomatic, (C) Number of cumulative deaths, (D) Number of hospitalised, (E) Number of ICU admissions

Source: own elaboration.

**Scenario 4: High efficacy (and effectiveness) of the vaccine, strict non-pharmacological measures**

**Scenario 4A**

|  | (A)  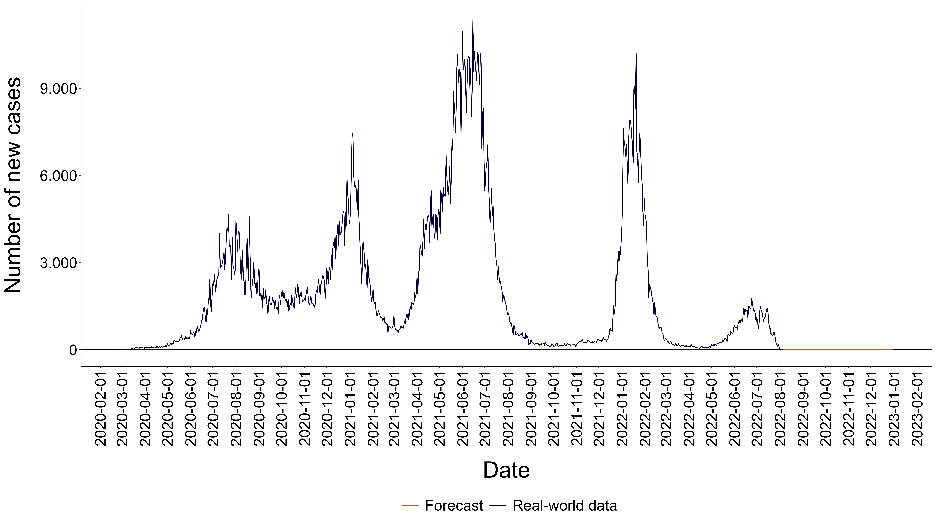 | (B)  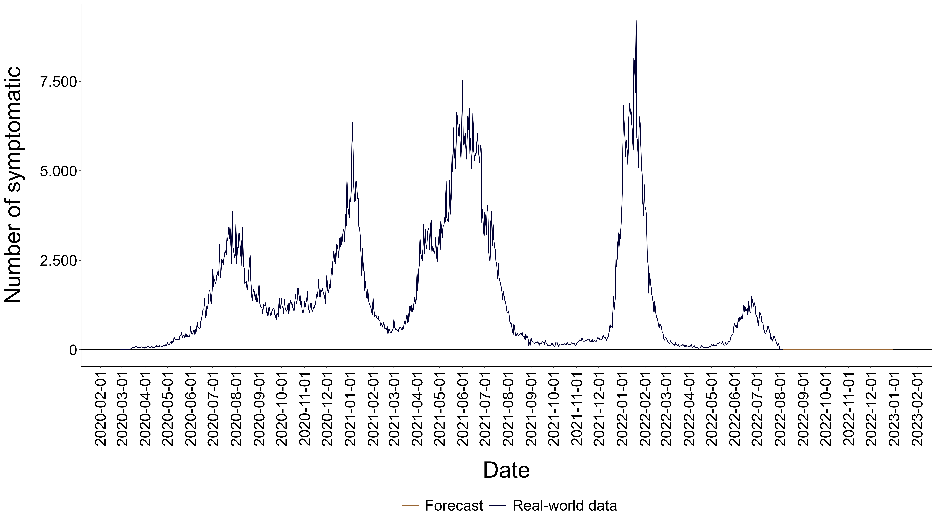 |
| --- | --- | --- |
|  | (C)  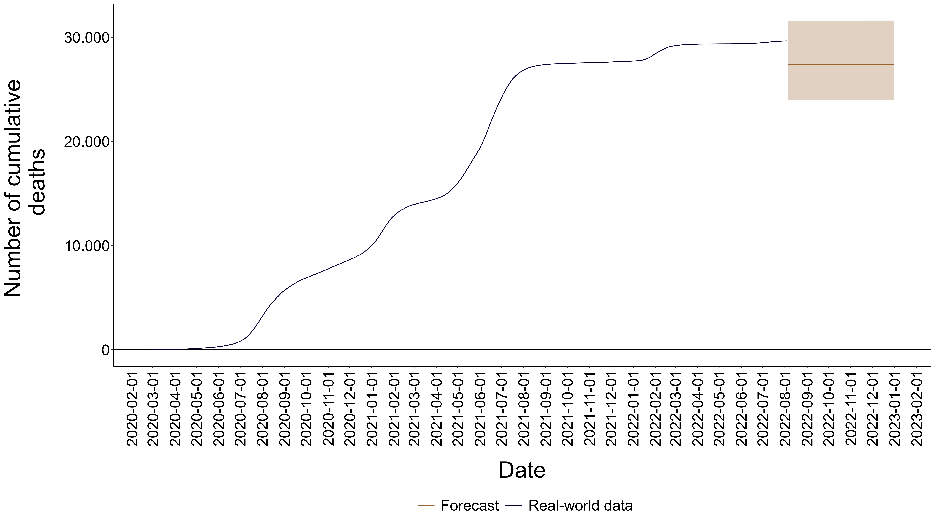 | (D)  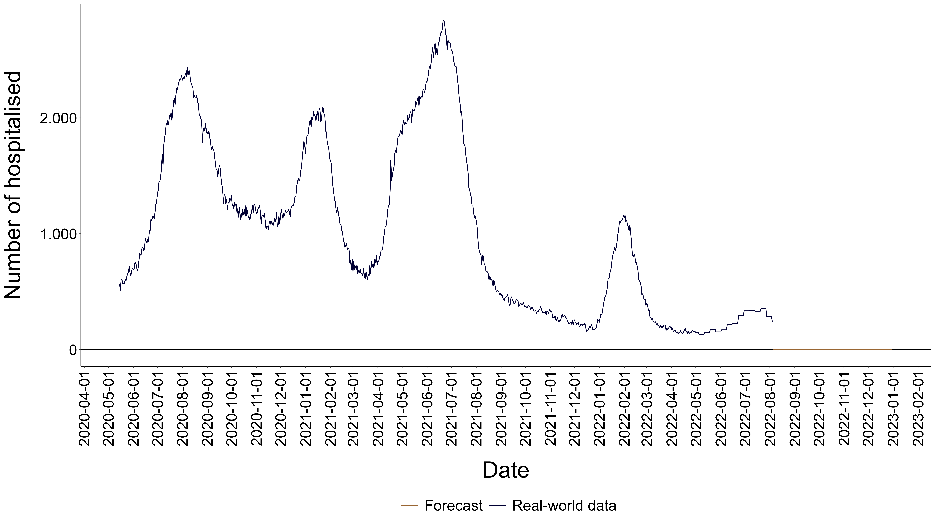 |
|  | (E)  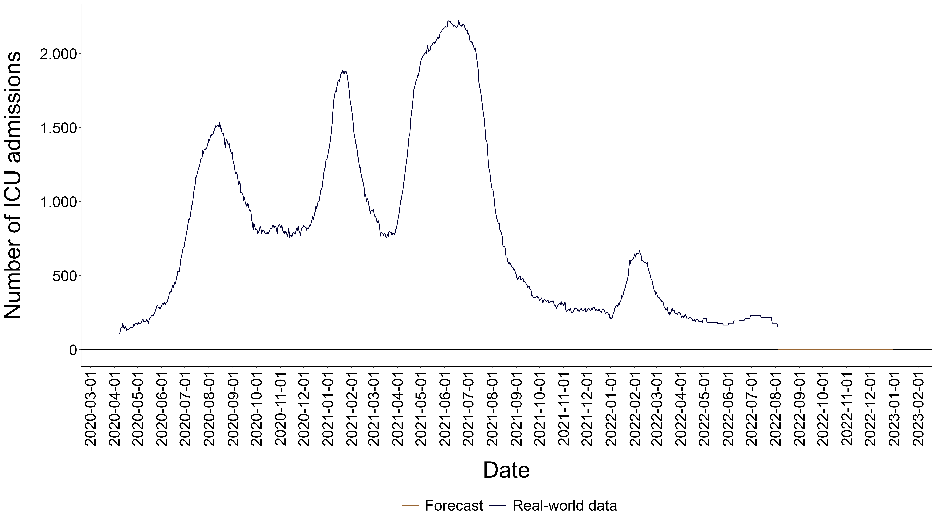 | |

**Figure 13.** Predicted health outcomes of SARS-CoV-2 in Bogotá. (A) Number of new cases, (B) Number of symptomatic, (C) Number of cumulative deaths, (D) Number of hospitalised, (E) Number of ICU admissions

Source: own elaboration.

**Scenario 4B**

|  | (A)  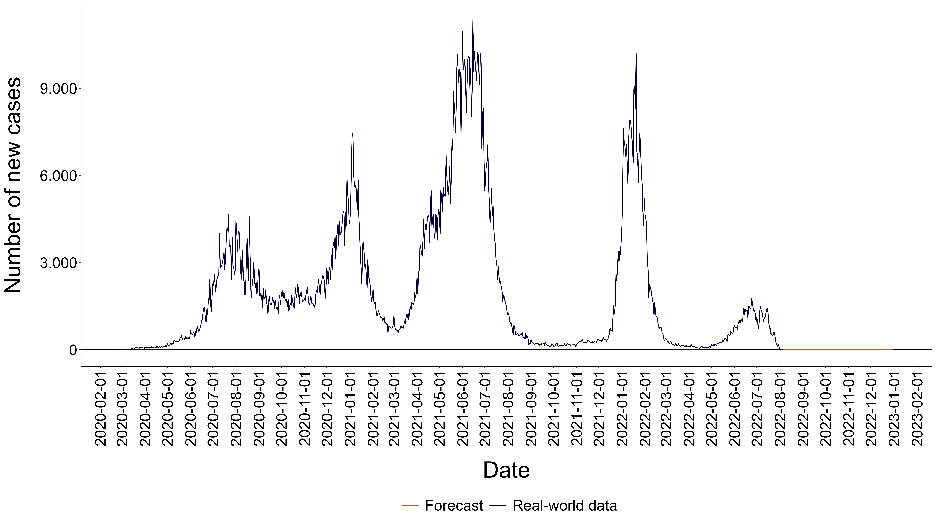 | (B)  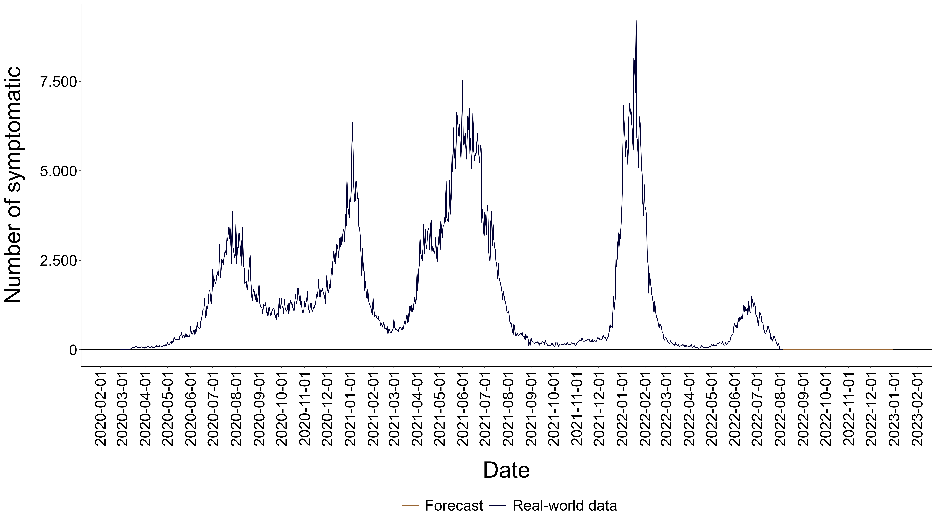 |
| --- | --- | --- |
|  | (C)  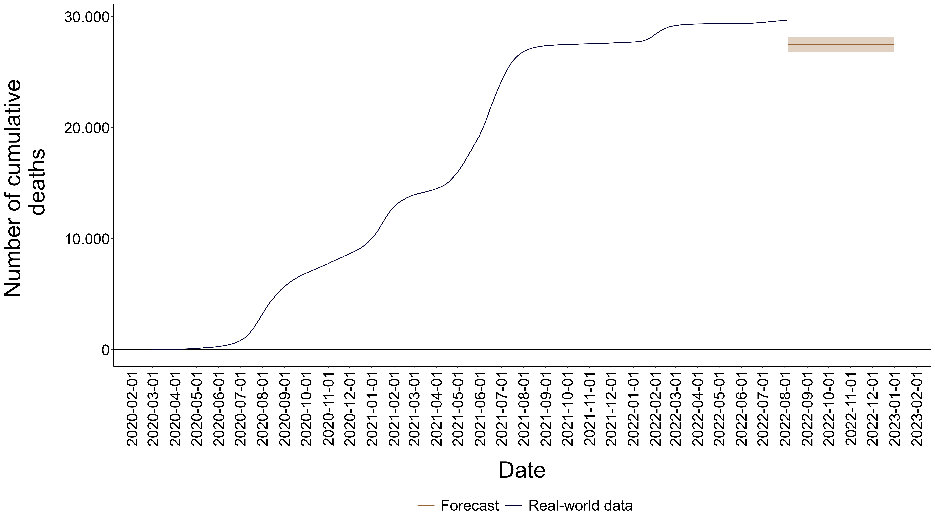 | (D)  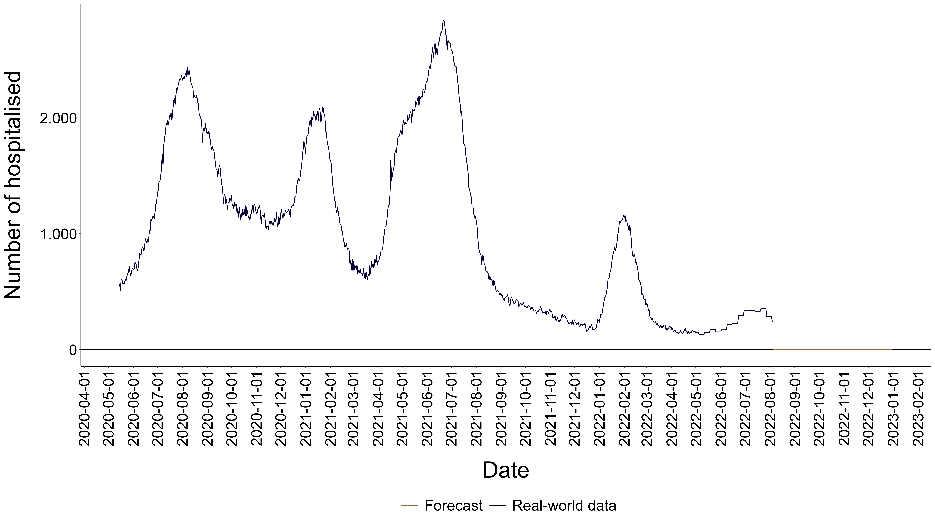 |
|  | (E)  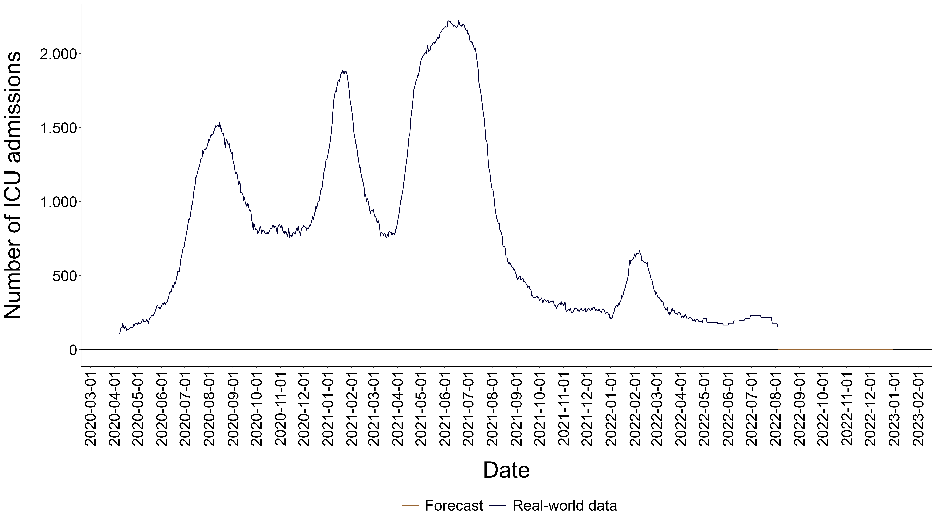 | |

**Figure 14.** Predicted health outcomes of SARS-CoV-2 in Bogotá. (A) Number of new cases, (B) Number of symptomatic, (C) Number of cumulative deaths, (D) Number of hospitalised, (E) Number of ICU admissions

Source: own elaboration.

**Scenario 4C**

|  | (A)  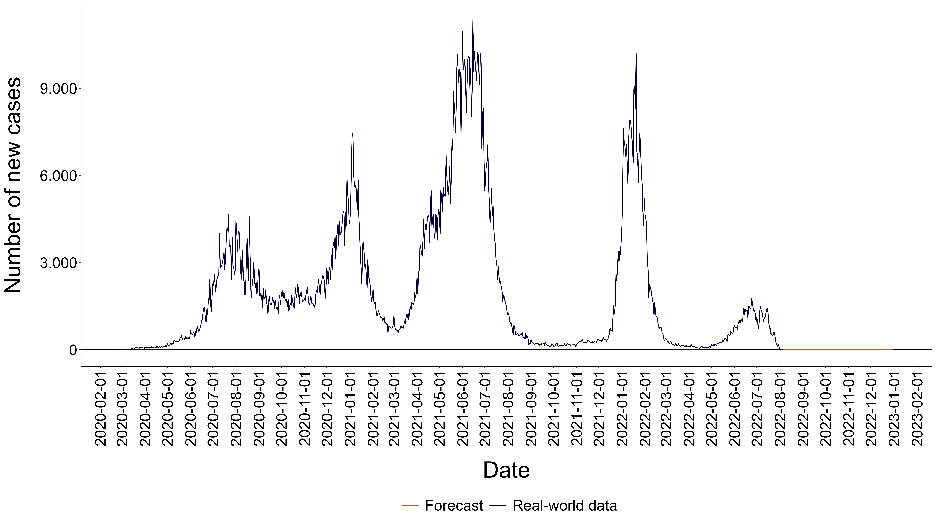 | (B)  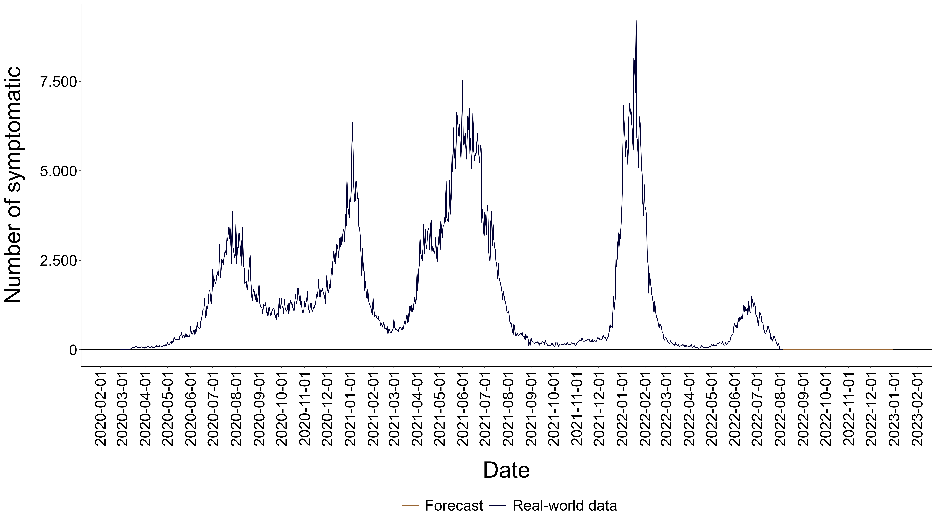 |
| --- | --- | --- |
|  | (C)  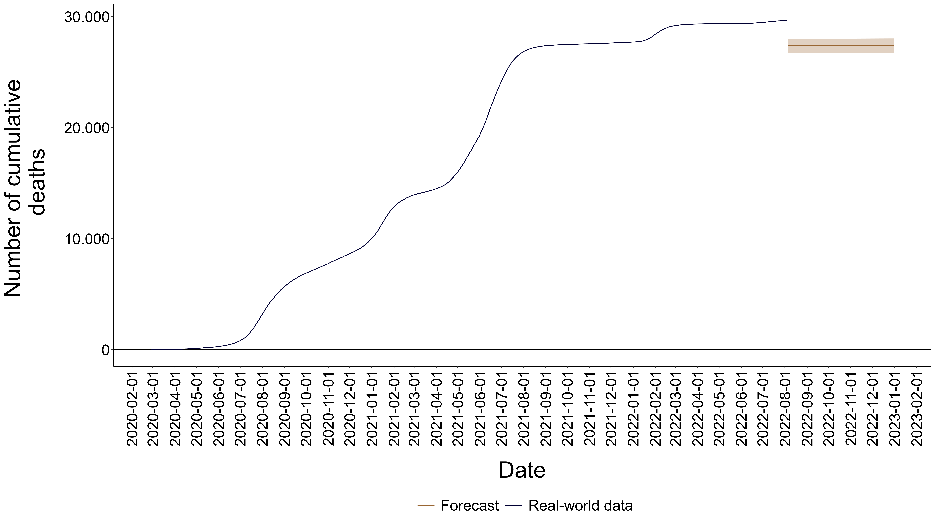 | (D)  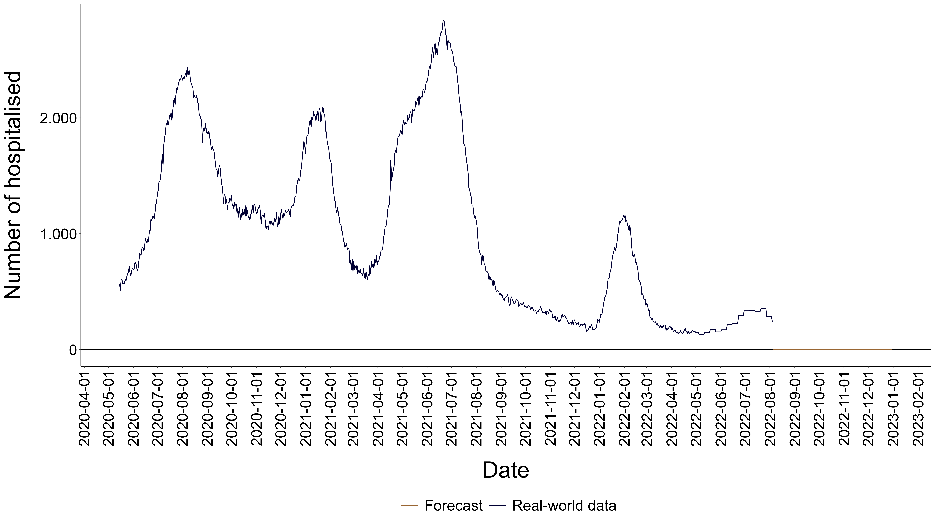 |
|  | (E)  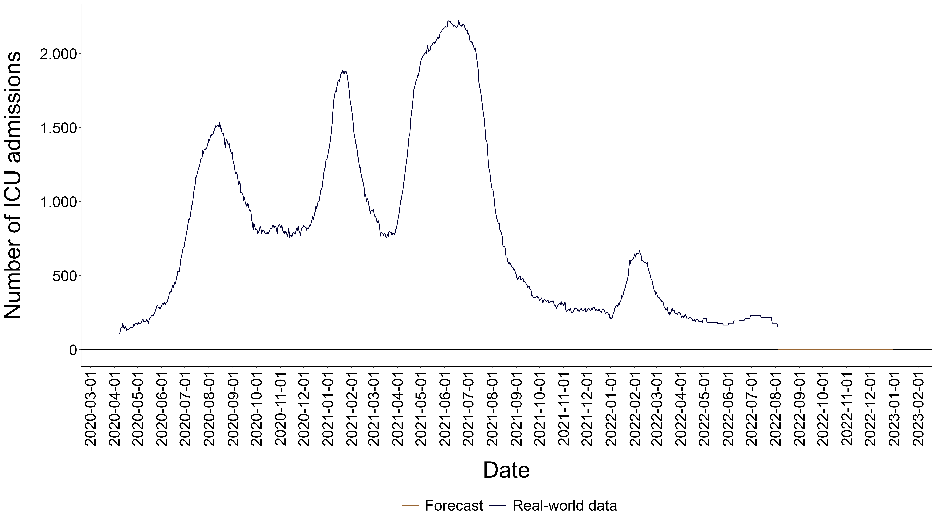 | |

**Figure 15.** Predicted health outcomes of SARS-CoV-2 in Bogotá. (A) Number of new cases, (B) Number of symptomatic, (C) Number of cumulative deaths, (D) Number of hospitalised, (E) Number of ICU admissions

Source: own elaboration.

**Scenario 4D**

|  | (A)  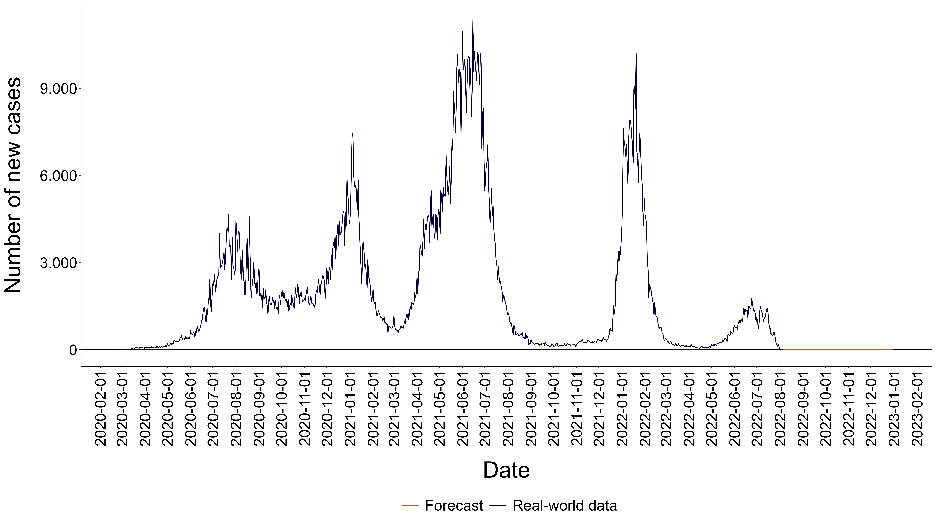 | (B)  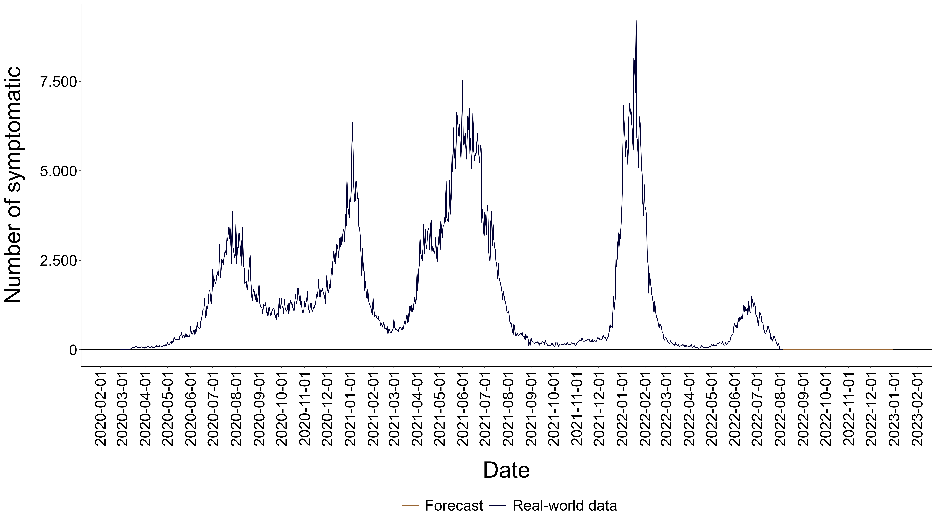 |
| --- | --- | --- |
|  | (C)  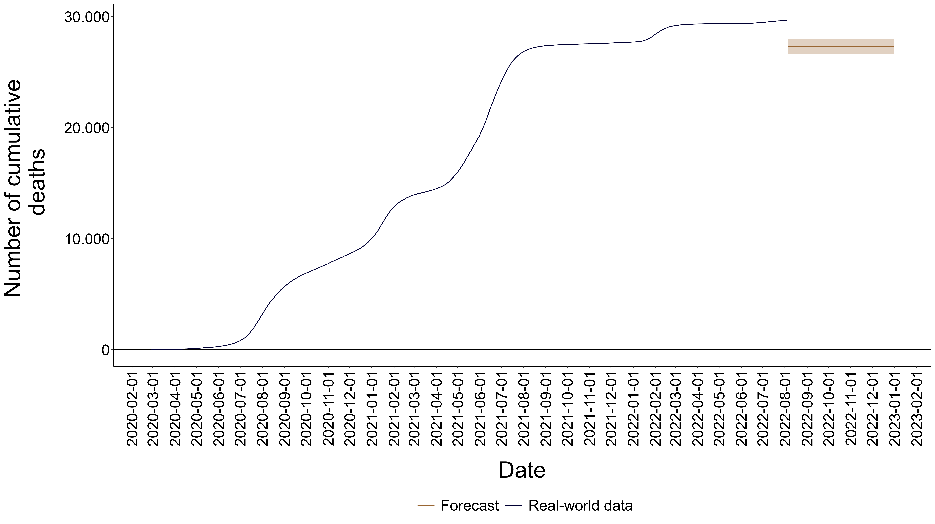 | (D)  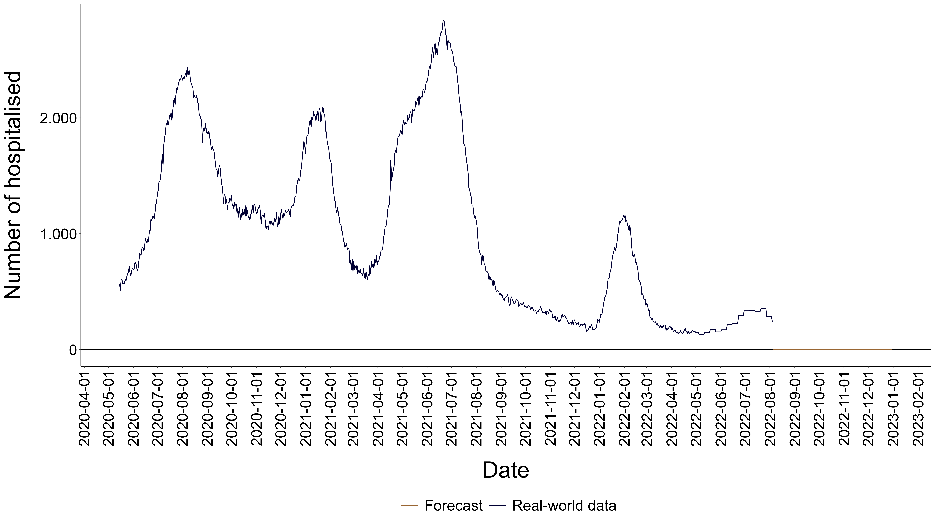 |
|  | (E)  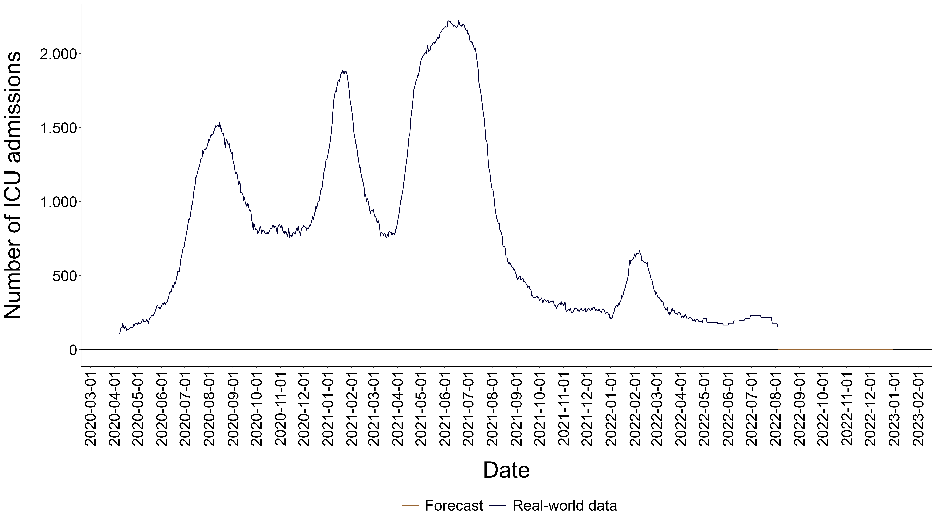 | |

**Figure 16.** Predicted health outcomes of SARS-CoV-2 in Bogotá. (A) Number of new cases, (B) Number of symptomatic, (C) Number of cumulative deaths, (D) Number of hospitalised, (E) Number of ICU admissions

Source: own elaboration.
